# Multi-omics analysis of *Helicobacter pylori*–associated gastric cancer identifies hub genes as a novel therapeutic biomarker

**DOI:** 10.1093/bib/bbaf241

**Published:** 2025-05-30

**Authors:** Sara H Mohamed, Mohamed Hamed, Hussain A Alamoudi, Zayd Jastaniah, Fadhl M Alakwaa, Asmaa Reda

**Affiliations:** Department of Microbiology, Egyptian Drug Authority (EDA), formerly National Organization for Drug Control and Research (NODCAR), Giza 14281, Egypt; Institute for Biostatistics and Informatics in Medicine and Ageing Research (IBIMA), Rostock University Medical Center, Rostock 18057, Germany; Faculty of Media Engineering and Technology, German University in Cairo, Cairo 11835, Egypt; Radiation Oncology Department, Oncology Center in East Jeddah Hospital (Jeddah First Health Cluster), Rabigh, Saudi Arabia; Center of Nanotechnology, King Abdulaziz University, Jeddah 21589, Saudi Arabia; Center of Nanotechnology, King Abdulaziz University, Jeddah 21589, Saudi Arabia; Department of Internal Medicine, Faculty of Medicine, King Abdulaziz University, Rabigh, Saudi Arabia; Department of Internal Medicine, Division of Nephrology, University of Michigan, Ann Arbor, MI, United States; Center of Nanotechnology, King Abdulaziz University, Jeddah 21589, Saudi Arabia; Zoology Department, Computational Biology and Bioinformatics Division, Faculty of Science, Benha University, Benha 12613, Egypt

**Keywords:** Transcriptome analysis, Biomarkers discovery, Hub genes, Therapeutic targets, Gastric cancer, *Helicobacter pylori*

## Abstract

*Helicobacter pylori* infection is one of the most common gastric pathogens; however, the molecular mechanisms driving its progression to gastric cancer remain poorly understood. This study aimed to identify the key transcriptomic drivers and therapeutic targets of *H. pylori*–associated gastric cancer through an integrative transcriptomic analysis. This analysis integrates microarray and RNA-seq datasets to identify significant differentially expressed genes (DEGs) involved in the progression of *H. pylori*–associated gastric cancer. In addition to independent analyses, data were integrated using ComBat to detect consistent expression patterns of hub genes. This approach revealed distinct clustering patterns and stage-specific transcriptional changes in common DEGs across disease progression, including *H. pylori* infection, gastritis, atrophy, and gastric cancer. Genes such as *TPX2*, *MKI67*, *EXO1*, and *CTHRC1* exhibited progressive upregulation from infection to cancer, highlighting involvement in cell cycle regulation, DNA repair, and extracellular matrix remodeling. These findings provide insights into molecular shifts linking inflammation-driven infection to malignancy. Furthermore, network analysis identified hub genes, including *CXCL1*, *CCL20*, *IL12B*, and *STAT4*, which are enriched in immune pathways such as chemotaxis, leukocyte migration, and cytokine signaling. This emphasizes their role in immune dysregulation and tumor development. Expression profiling demonstrated the upregulation of hub genes in gastric cancer and stage-specific changes correlating with disease progression. Finally, drug–gene interaction analysis identified therapeutic opportunities, with hub genes interacting with approved drugs like abatacept and zoledronic acid, as well as developmental drugs such as adjuvant and relapladib. These findings highlight the key role of these hub genes as biomarkers and therapeutic targets, providing a foundation for advancing precision medicine in *H. pylori*–associated gastric cancer. Overall, this study paves the way for advancing precision medicine in *H. pylori*–associated gastric cancer by providing insights into the development of early detection biomarkers, risk stratification, and targeted therapies. This supports the clinical translation of precision medicine strategies in *H. pylori*–associated gastric cancer.

## Introduction

*Helicobacter pylori* is a Gram-negative, microaerophilic bacterium first identified in the early 1980s by Barry J. Marshall and J. Robin Warren [1]. It is now recognized as one of the most prevalent gastric pathogens, colonizing the stomachs of approximately half of the global population [2–4]. Infection rates vary significantly by geographic region [3, 5]. Its biological characteristics to survive in the highly acidic environment of the human stomach highlight its clinical significance and explain its widespread prevalence.

*Helicobacter pylori* is crucial in developing various gastric disorders, including infection, gastritis, and atrophy [6]. In 2005, Marshall and Warren were awarded the Nobel Prize in Medicine for their study on the role of *H. pylori* in chronic inflammation and its correlation with peptic ulcer disease [7, 8]. Despite these findings, the precise mechanisms by which *H. pylori* generates chronic inflammation and promotes gastric cancer remain unknown. This knowledge gap highlights the need for further investigation into the cellular and molecular mechanisms underlying the development of *H. pylori*–associated gastric cancer to help in the development of effective therapy or prevention of the disease.

Identifying differentially expressed genes (DEGs) and hub genes in *H. pylori*–associated disease progression from infection to gastritis, atrophy, and, ultimately, gastric cancer is critical for enhancing our understanding of the molecular mechanisms driving this progression. DEGs provide insights into specific gene expression changes, while hub genes, identified through network analyses, highlight key regulatory nodes that possibly drive pathophysiological processes [9–11]. These findings hold significant translational value, enabling the development of targeted drug therapies and personalized medicine approaches while improving prognostic tools for gastric cancer. Integrating multi-omics approaches offers a comprehensive view of these molecular changes [12, 13]. For instance, microarray techniques enable high-throughput analysis of gene expression patterns, while RNA-seq provides high sensitivity and resolution, detecting novel transcripts. Combining these approaches using combat strategy could facilitate the discovery of therapeutic biomarkers. This integrative strategy bridges gaps in understanding the link between *H. pylori* infection and gastric cancer and supports the development of precision medicine approaches to combat this aggressive malignancy [14–16].

**Figure 1:**
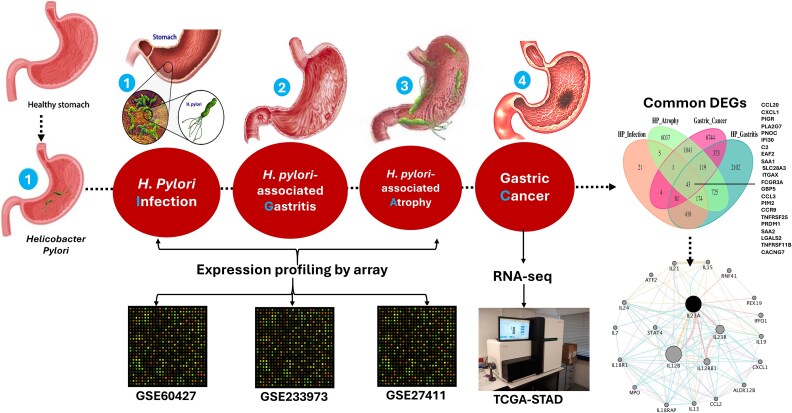
Illustration of the pipeline for identifying molecular signatures related to *H. pylori* disease progression, showing the transition from infection in a healthy stomach to gastritis, atrophy, and ultimately gastric cancer. The workflow integrates transcriptomic analyses, including microarray data from datasets (GSE60427, GSE233973, GSE27411) for earlier disease stages and RNA-seq data from TCGA-STAD for gastric cancer. A Venn diagram highlights common DEGs across stages, followed by network analysis that illustrates their interactions and roles in gastric cancer pathways.

This study aims to investigate the molecular mechanisms linking *H. pylori* infection and gastric cancer, focusing on identifying key DEGs involved in disease progression through integrative transcriptomic data analysis (Fig. 1). Additionally, it explores their role in protein–protein interactions (PPIs), which may serve as potential therapeutic biomarkers for gastric cancer progression, as well as the examination of drug–gene interactions and survival outcomes.

## Materials and methods

### Data selection and preprocessing

**Figure 2:**
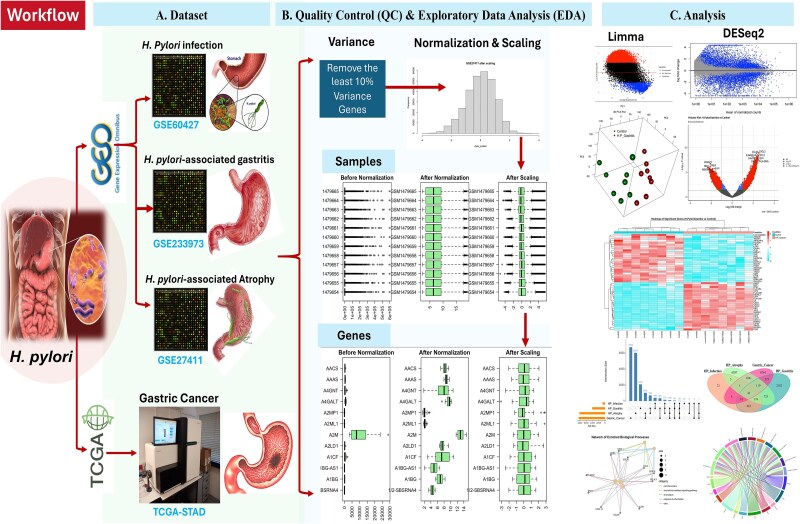
Workflow for data processing and analysis of *H. pylori*–associated gastric diseases. The workflow integrates datasets from GEO (GSE60427, GSE233973, GSE27411) and TCGA-STAD to analyze the progression from *H. pylori* infection to gastric cancer. (A) Datasets include gene expression profiles representing stages of *H. pylori* infection, gastritis, atrophy, and gastric cancer. (B) Quality control (QC) and exploratory data analysis (EDA) involve removing the least variable 10% of genes, normalization, and scaling to ensure data consistency. Sample and gene distributions before and after normalization and scaling are shown. (C) Differential expression analysis was performed using limma for microarray data and DESeq2 for RNA-seq data, identifying significant DEGs for downstream functional analysis, including Venn analysis, heatmaps, pathway enrichment, and network analysis.

To investigate datasets related to gastric cancer, RNA-seq raw expression data and corresponding clinical information for TCGA-Stomach Adenocarcinoma (STAD) patients were obtained from the TCGA database (https://portal.gdc.cancer.gov/). Additionally, three gene expression datasets associated with *H. pylori* were retrieved from the GEO database: GSE27411 (GPL6255 Illumina humanRef-8 v2.0 expression beadchip), GSE60427 (GPL17077 Agilent-039494 SurePrint G3 Human GE v2 8x60K Microarray 039381), and GSE233973 (GPL21185 Agilent-072363 SurePrint G3 Human GE v3 8x60K Microarray 039494) (Fig. 2A) [12, 13]. Herein, three selected GEO datasets are based on their relevance to *H. pylori*–associated disease signature stages, high data quality, and the availability of annotated sample groups (case versus control). Clinical metadata and sample distribution are summarized in Supplementary Table S1.

### Quality control and exploratory data analysis

Quality control (QC) and exploratory data analysis (EDA) pipelines were implemented to prepare the data for downstream analyses. Initially, all missing values (NAs) and duplicate entries were identified and removed to maintain data integrity. Genes with low variability, defined as the lowest 10% based on variance across samples, were filtered out to reduce noise and retain biologically meaningful signals. The data were then normalized using log2 transformation to adjust for differences in expression levels and ensure consistency across samples. Following normalization, scaling was applied to standardize feature distributions, ensuring uniformity. Visual inspections using boxplots and histograms before and after normalization and scaling verified the effectiveness of these preprocessing steps. The resulting clean and quality-controlled datasets were subsequently utilized for downstream analyses, including differential expression and functional enrichment analysis, forming a robust foundation for high-confidence biological interpretation (Fig. 2B).

### Identification of differentially expressed genes

DEGs were identified using limma package within the R software for microarray data and the DESeq2 package for RNA-seq data. The limma package was employed to analyze DEGs between *H. pylori* disease and control samples in GSE27411, GSE60427, and GSE233973 datasets. For the TCGA-STAD RNA-seq dataset, the DESeq2 package was utilized to identify DEGs between tumor and normal samples. DEGs were filtered based on a threshold of adjusted *P*-value <.05 and |log2 fold change| > 1, as per the criteria described [17]. A Venn diagram and an upset plot were generated to identify common DEGs across the datasets to extract overlapping DEGs from the four DEG lists (TCGA-STAD, GSE27411, GSE60427, and GSE233973). These overlapping DEGs were considered key targets and subjected to subsequent analyses (Fig. 2C).

### Functional enrichment analysis

Functional enrichment analysis was conducted using the clusterProfiler package in R software to explore Gene Ontology (GO) and Over-Representation Analysis for the common DEGs. The enrichGO function was applied to identify enriched biological processes, molecular functions, and cellular components, using a significance threshold of *P* <.05 with Benjamini–Hochberg correction for multiple testing. Gene sets from the Molecular Signatures Database (MSigDB) were accessed through the msigdbr package. Data visualization was performed using the ggplot2 package in R software, ensuring a clear representation of the results [18].

### Pathway analysis

Pathway analyses were performed to identify biological pathways associated with the differentially expressed genes. KEGG pathway enrichment analysis and reactome pathway analysis were performed using the clusterProfiler package in R software. The significance level was set at *P* <.05. Finally, the results were visualized using the “ggplot2” package in R software [17].

### Gene–gene interactions

Investigation of the gene–gene interaction network for the common genes was performed using GeneMANIA (http://www.genemania.org) to explore interconnections between output proteins. The analysis included physical interactions, co-expression, predicted interactions, co-localization, shared pathways, genetic interactions, and common protein domains [11, 19].

### Protein–protein interaction analysis

The PPI network was constructed using the STRING database (https://string-db.org/) and analyzed in Cytoscape software. The initial network was generated in the STRING database and then imported into Cytoscape for further exploration. Hub genes were identified using the CytoHubba plugin in Cytoscape. Multiple algorithms, including Density of Maximum Neighborhood Component (DMNC), Degree Centrality (Degree), Maximal Clique Centrality (MCC), Edge Percolated Component (EPC), and Closeness Centrality (Closeness), were applied to rank and select the top hub genes. This multi-algorithm approach ensured robust identification of key genes involved in the network [9].

### Drug–gene interaction

Drug–gene interactions were analyzed using the Drug-Gene Interaction Database (DGIdb) (https://dgidb.org/) to identify potential drugs targeting the hub genes. U.S. Food and Drug Administration (FDA)-approved drugs were selected and visualized using the ggplot2 package in R software. The dplyr package was employed to rank and extract the top 10 drugs based on interaction scores for further analysis. Pairwise interactions were visualized by preparing the data to include relevant columns for drugs, genes, and interaction scores, ensuring clarity in the presentation of the results [9].

### *In silico* pharmacokinetic analysis of FDA-approved and developmental drugs

To assess the pharmacokinetic properties and drug-likeness of FDA-approved and developmental drugs identified through drug–gene interaction analysis, an *in silico* analysis has been performed using the SwissADME web tool (http://www.swissadme.ch/) [20]. The drugs analyzed included FDA-approved drugs (adenosine, mercaptopurine, mitomycin, naproxen sodium, pamidronate, toremifene, zoledronic acid, uridine) and developmental drugs (ABX-1431, GSK-2879454, KT-109, Picibanil, WWL-123, WWL-70), which were selected based on their high interaction scores with upregulated hub genes. The chemical structures of these compounds were retrieved in SMILES (Simplified Molecular Input Line Entry System) format from the PubChem database (https://pubchem.ncbi.nlm.nih.gov/) and uploaded to SwissADME for analysis. SwissADME computed key physicochemical properties, including lipophilicity (WLOGP), molecular weight (SIZE), polarity (Topological Polar Surface Area, TPSA), solubility (INSOLU), saturation (INSATU), and flexibility (FLEX), which were visualized in radar plots to evaluate drug-likeness based on Lipinski’s Rule of Five and other pharmacokinetic criteria. Additionally, SwissADME predicted pharmacokinetic parameters such as blood–brain barrier (BBB) permeability, human intestinal absorption (HIA), and P-glycoprotein (PGP) substrate status (PGP+ or PGP−). The TPSA and WLOGP values were analyzed to assess oral bioavailability, with compounds falling within the optimal range (TPSA <140 Å^2^ and WLOGP between −0.4 and 5.6) considered favorable for drug development. Results were visualized using radar plots for physicochemical properties and scatter plots for TPSA versus WLOGP, with color-coding to indicate predicted pharmacokinetic actions (BBB, HIA, PGP+/−).

### Expression of hub genes across gastric cancer stages

GEPIA (http://gepia.cancer-pku.cn/detail.php?clicktag=matrix###) was used to analyze the expression patterns of hub genes across the four stages of gastric cancer [21], also used to compare the expression levels of these hub genes in gastric cancer samples and normal samples. Normal expression data for the hub genes were obtained from the GTEx Portal (https://gtexportal.org/home/), providing a baseline for comparison [22]. This analysis facilitated the evaluation of hub gene expression dynamics in relation to disease progression and normal tissue.

### Cross-validation and survival analysis of the hub genes

To assess the prognostic significance of the identified hub genes in *H. pylori*–associated gastric cancer, survival analysis was conducted using RNA-seq and clinical data from the TCGA-STAD cohort. Patients were stratified into high-risk and low-risk groups based on the combined expression of selected hub genes. The risk stratification was performed using the SurvExpress web platform (http://victortrevino.bioinformatics.mx:8080/Biomatec/SurvivaX.jsp), where a multivariate Cox proportional hazards model was used to calculate a prognostic index (risk score) for each patient. Observations with scores above the median were classified as high risk, and those below the median were classified as low risk. The primary clinical endpoint was overall survival, with a maximum follow-up period of 10 years (3650 days). Survival data were right-censored and included overall survival time and vital status (alive or deceased). Additionally, Kaplan–Meier survival curves were generated to assess differences in survival distributions among risk groups, and statistical significance was evaluated using the log-rank test (*P* < .05). Hazard ratios (HRs) were calculated using the Cox model to quantify the impact of gene expression on survival outcomes. The analysis was designed to evaluate the independent prognostic value of gene expression, and therefore no additional clinical covariates (e.g. age, sex, tumor stage) were included in the model. To validate gene expression patterns between risk groups, box plots and heatmaps were generated, and differences were tested using Student’s *t*-test. In addition to the combined risk score analysis, survival analysis of individual hub genes was conducted using the GEPIA platform (http://gepia.cancer-pku.cn). Patients were divided into high- and low-expression groups based on the median expression value for each gene. Kaplan–Meier plots and log-rank tests were used to determine the prognostic relevance of each gene showing significant associations with poor overall survival [23].

### ComBat integration and analysis

The ComBat function from the sva package in R software was utilized to integrate gene expression data from three microarray datasets (GSE27411, GSE60427, and GSE233973) and RNA-seq data from TCGA-STAD. This integration was essential for harmonizing datasets and correcting platform-specific batch effects. Prior to integration, data were normalized and scaled to ensure consistency across platforms. The integrated expression matrix was subsequently used to analyze the expression patterns of common DEGs across disease stages, including *H. pylori* infection, gastritis, atrophy, and gastric cancer. Expression patterns were visualized using heatmaps and line plots based on continuous gene expression values without applying binary thresholds to capture dynamic transcriptional changes throughout disease progression. For downstream analyses, including survival analysis, patients were stratified into high- and low-expression groups using the median gene expression value as the threshold. This approach preserved the granularity of expression profiles during integration while enabling the application of a biologically meaningful cutoff for clinical outcome associations [24, 25].

## Results

### Mapping the progression of *H. pylori* disease through differential expression analysis

**Figure 3:**
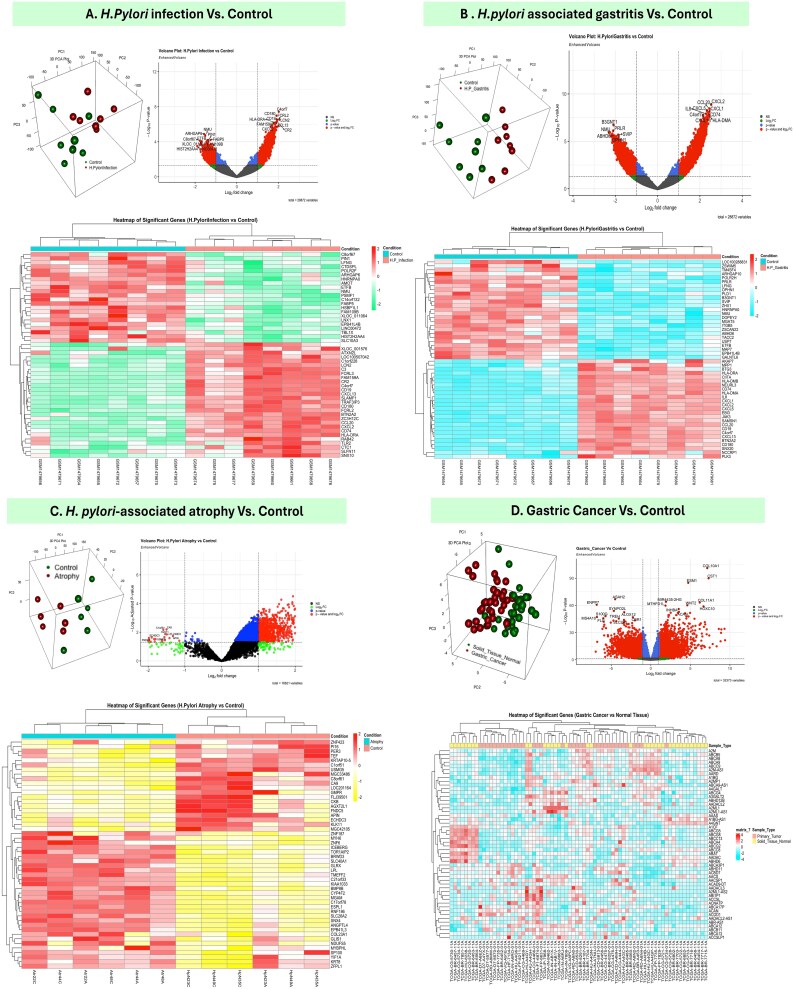
Illustration of the transcriptional changes across *H. pylori*–associated stages, from infection to gastric cancer. (A) In *H. pylori* infection versus control, PCA shows distinct clustering, volcano plots identify DEGs involved in immune responses and inflammation, and heatmaps highlight upregulated immune-related genes. (B) In *H. pylori*–associated gastritis, transcriptional shifts are evident, with PCA showing clear separation from controls, volcano plots identifying DEGs linked to cytokine signaling and epithelial repair, and heatmaps emphasizing upregulated inflammation-associated genes. (C) In *H. pylori*–associated atrophy, DEGs related to extracellular matrix remodeling and epithelial degradation are prominent, with PCA indicating significant transcriptional changes and heatmaps displaying unique expression profiles distinguishing atrophy from earlier stages. (D) In gastric cancer versus control, PCA shows the most pronounced separation, volcano plots identify DEGs associated with cell cycle regulation and oncogenic pathways, and heatmaps reveal distinct gene expression patterns characteristic of cancer progression. These findings collectively demonstrate the progressive molecular changes underlying *H. pylori*–associated disease.

To investigate the progression of *H. pylori* infection from a healthy stomach to gastritis, atrophy, and ultimately gastric cancer, differential expression analysis was conducted across four datasets revealing the molecular changes associated with each stage of disease progression. In the *H. pylori* infection stage (Fig. 3A), principal component analysis (PCA) demonstrated a clear separation between infected and control samples, highlighting distinct transcriptional profiles. Volcano plot analysis identified key DEGs involved in immune responses and inflammation, including cytokines and chemokines. Heatmap visualization reinforced these findings, showing consistent upregulation of immune-related genes in infected samples compared to controls. As the infection progressed to *H. pylori*–associated gastritis (Fig. 3B), PCA clustering continued to show distinct segregation of samples, reflecting further transcriptional shifts. Volcano plot revealed DEGs associated with cytokine signaling and epithelial repair, suggesting an inflammatory response and tissue remodeling. Heatmaps provided a detailed representation of these changes, with inflammation-related genes prominently upregulated in gastritis samples.

At the atrophy stage (Fig. 3C), PCA revealed clear segregation between atrophic and control samples, indicating significant transcriptional alterations. Volcano plot identified DEGs linked to extracellular matrix remodeling and epithelial degradation, central to tissue atrophy. Heatmaps highlighted a unique expression profile, distinguishing atrophic samples from earlier stages and controls. In the final stage of gastric cancer (Fig. 3D), PCA showed the most pronounced separation between tumor and normal tissues, indicating extensive molecular reprogramming. Volcano plot identified numerous DEGs associated with cell cycle regulation, angiogenesis, and oncogenic pathways, while heatmaps revealed distinct gene expression patterns that separated cancer samples from controls. This progression of transcriptional changes provides a comprehensive understanding of the molecular landscape underlying *H. pylori*–associated disease, offering valuable insights for identifying biomarkers and therapeutic targets (Fig. 3).

### Common DEGs in *H. pylori*–associated gastric cancer for therapeutic biomarker discovery

**Figure 4:**
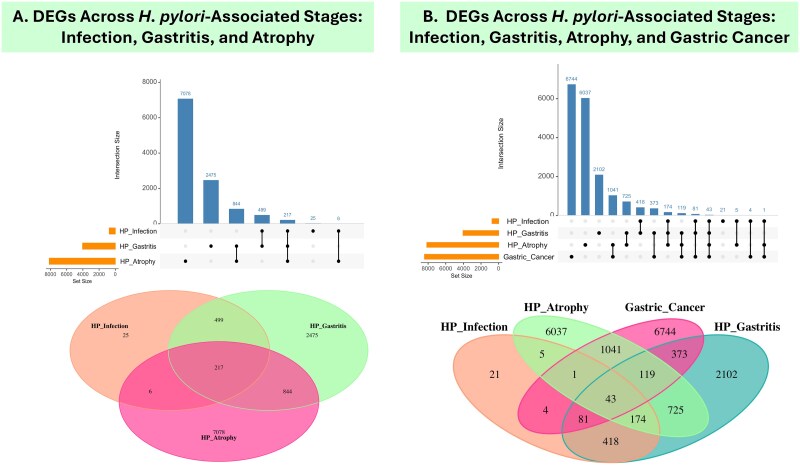
Illustration highlighting DEGs across *H. pylori*–associated stages. (A) The UpSet plot and Venn diagram illustrate 217 DEGs shared among infection, gastritis, and atrophy, with stage-specific subsets identified. (B) Including gastric cancer, 43 DEGs overlap across all stages, with the largest intersection between atrophy and cancer, highlighting a molecular link between precancerous and malignant states.

To address the question, what are the common DEGs in disease progression that can guide therapeutic biomarker discovery? An analysis was conducted using UpSet plots and Venn diagrams to identify shared and unique DEGs across the datasets. The UpSet plot highlighted the intersections of DEGs across datasets representing *H. pylori* infection, gastritis, and atrophy, revealing 217 DEGs shared among the three *H. pylori* stages (Fig. 4A). Furthermore, an overlap of 43 DEGs was identified across the TCGA-STAD dataset and the three *H. pylori* stages, representing genes consistently involved in disease progression (Fig. 4B). The Venn diagram complemented this analysis by illustrating the overlap and uniqueness of DEGs across the stages. Notably, the most significant overlap was observed between atrophy and gastric cancer, suggesting a strong molecular link between these stages. This integrative analysis provides valuable insights into the progression of *H. pylori*–associated disease. It identifies common DEGs as potential therapeutic targets, offering a foundation for biomarker discovery and personalized treatment strategies.

### Network analysis of GO enrichment reveals key biological pathways

**Figure 5:**
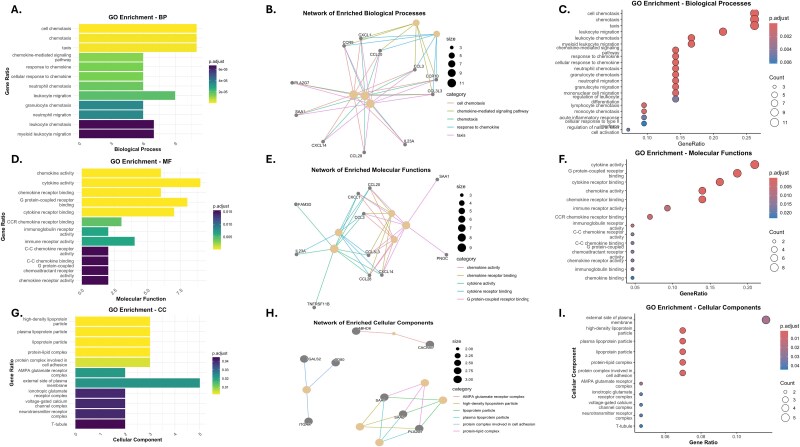
Depiction of the Gene Ontology (GO) enrichment analysis of differentially expressed genes (DEGs) across biological processes (A–C), molecular functions (D–F), and cellular components (G–I). A–C: Enriched biological processes include chemotaxis, leukocyte migration, and immune response, with a gene network highlighting interactions among key chemokines (*CCL20*, *CXCL1*, *CXCL2*, *CXCL8*). D–F: Molecular functions enriched include chemokine activity, receptor binding, and cytokine signaling, with networks emphasizing immune and inflammatory pathways mediated by genes such as *CXCR2* and *CCR1*. G–I: Cellular component enrichment identified extracellular regions and lipoprotein particles, with networks showcasing interactions among genes like APOA1 and LBP, highlighting their roles in immune regulation and extracellular signaling. These results reveal the central pathways and molecular interactions driving *H. pylori*–associated disease progression.

GO enrichment analysis revealed critical biological pathways and gene networks associated with *H. pylori*–associated gastric cancer progression, categorized into biological processes (BP), molecular functions (MF), and cellular components (CC). BP enrichment analysis (Fig. 5A–C) identified pathways such as chemotaxis, cell chemotaxis, and leukocyte migration, which are pivotal in immune responses to infection. The network visualization (Fig. 5B) highlighted key genes, including *CCL20, CXCL1, CXCL2*, and *CXCL8*, forming highly connected hubs in pathways related to chemokine-mediated signaling and myeloid leukocyte activation, suggested to play a significant role in mediating inflammation and immune cell recruitment, marking the early response to *H. pylori* infection and the transition to chronic inflammation in gastritis.

MF analysis (Fig. 5D–F) revealed significant enrichment in chemokine activity, cytokine receptor binding, and G-protein-coupled receptor signaling. The network (Fig. 5E) showed strong interconnections among genes such as *CXCR2, CCR1*, and *CXCL8*, which are crucial in immune signaling and recruitment of inflammatory cells. These enriched pathways indicate the persistence of inflammation and immune dysregulation, contributing to epithelial damage and disease progression from gastritis to atrophy. Cellular component enrichment (Fig. 5G–I) identified the extracellular region, plasma lipoprotein particles, and high-density lipoprotein particles as key locations for functional activity. The network of enriched cellular components (Fig. 5H) highlighted genes such as *ABHD6*, *CACNG7*, *ITGAX*, *CD80*, *GAL2S*, *SAA1*, *SAA2*, and *PLA2G7*, suggesting a role in lipid metabolism and immune modulation within the extracellular environment. These pathways reflect metabolic reprogramming and extracellular matrix interactions characteristic of the transition to gastric atrophy and cancer.

### Pathway enrichment analysis highlights key signaling mechanisms in *H. pylori*–associated disease progression

Pathway enrichment analysis revealed critical molecular pathways driving *H. pylori*–associated disease progression, as shown in Supplementary Fig. S1. KEGG pathway analysis (A–B) identified pathways central to immune and inflammatory responses, including cytokine–cytokine receptor interaction, chemokine signaling, and Toll-like receptor signaling, all of which are pivotal in mediating the host’s defense mechanisms against *H. pylori* infection. The activation of these pathways plays a role in triggering inflammation and immune cell recruitment during the early stages of infection and gastritis.

Reactome pathway enrichment (C–D) further emphasized key signaling cascades, such as chemokine receptor binding, GPCR signaling, and interleukin-10 signaling, which are crucial for immune modulation and inflammatory signaling. These pathways highlight persistent immune dysregulation and tissue remodeling as the disease progresses to atrophy. MSigDB hallmark analysis (E–F) identified pathways like *KRAS* signaling up, *mTORC1* signaling, and *TNFA* signaling via *NFκB*, which are critical in promoting cell proliferation, survival, and chronic inflammation during the transition to gastric cancer. Collectively, these pathways reflect a continuum of molecular events driving *H. pylori*–associated gastric cancer progression, offering insights into potential therapeutic targets.

### Network analysis of DEGs and identification of hub gene interactions

**Figure 6:**
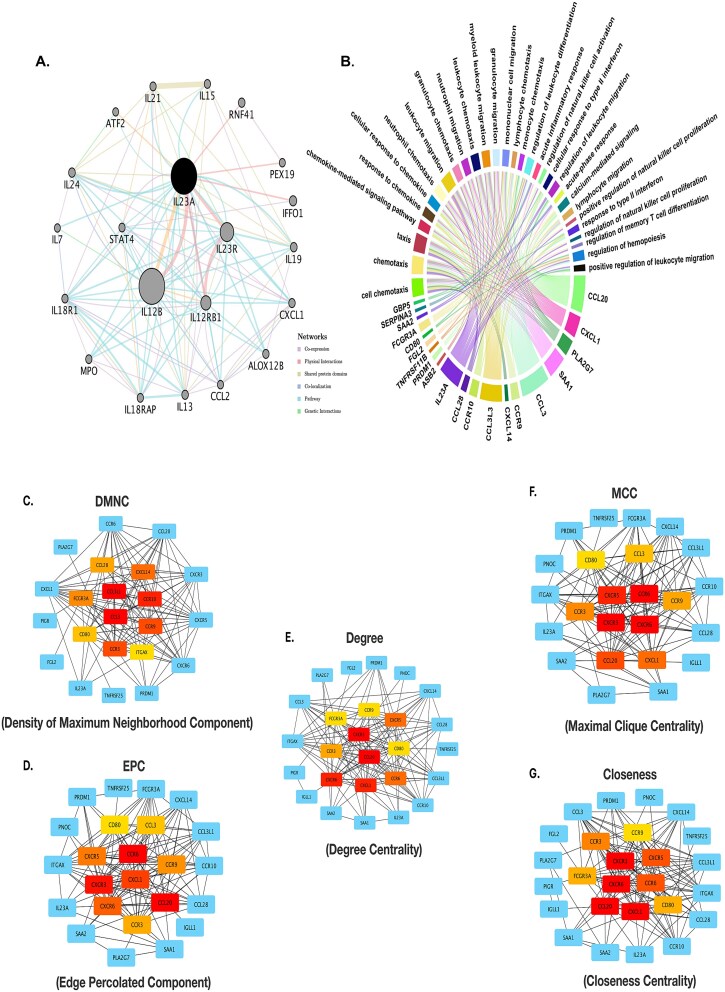
Network and centrality analyses of DEGs in *H. pylori*–associated gastric cancer progression. (A) The network highlights hub genes such as *IL12B, IL12A, CXCL1, IL23R,* and *STAT4,* which are central to immune and inflammatory pathways, interconnected through co-expression, physical interactions, and shared pathways. (B) The chord diagram integrates GO terms and gene associations, emphasizing critical processes like chemotaxis, leukocyte migration, and cytokine-mediated signaling. (C–G) Centrality analyses using DMNC, EPC, degree, MCC, and closeness consistently identify hub genes, particularly *CCL20, CXCL1*, and *IL12B,* as key regulators in the network.

To address the question, “What are the key molecular interactions and hub genes driving the progression of *H. pylori*–associated gastric cancer?”, a comprehensive network analysis was performed to discover critical pathways and gene interactions. The network of DEGs (Fig. 6A) revealed highly interconnected hubs, with genes such as *IL12A, IL12B, IL23R, CXCL1, STAT4*, and *CCL20* forming central nodes. These genes were enriched in immune-related pathways, including chemotaxis, leukocyte migration, and cytokine-mediated signaling, which are essential for immune cell recruitment and inflammatory responses. The chord diagram (Fig. 6B) further demonstrated the integration of GO terms and gene associations, highlighting significant processes such as positive regulation of leukocyte migration, immune cell activation, and chemokine signaling pathways.

To identify key hub genes within the network, centrality analyses were conducted using multiple algorithms, including Density of Maximum Neighborhood Component (DMNC, Fig. 6C), Edge Percolated Component (EPC, Fig. 6D), Degree Centrality (Fig. 6E), Maximal Clique Centrality (MCC, Fig. 6F), and Closeness Centrality (Fig. 6G). These analyses consistently identified genes such as *CCL20, CXCL1, IL12B*, and *STAT4* as central players, indicating their pivotal roles in maintaining network connectivity and influencing disease-related pathways. Notably, these hub genes are involved in immune signaling, cytokine production, and inflammatory responses, suggesting their potential as biomarkers or therapeutic targets.

The expression patterns of these hub genes across normal tissues, gastric cancer versus normal tissues, and different stages of gastric cancer further corroborate their significance. As shown in Supplementary Fig. S2 available online at http://bib.oxfordjournals.org/, the heatmap of transcript per million (TPM) values (Supplementary Fig. S2A available online at http://bib.oxfordjournals.org/) revealed that hub genes, including *CXCL1, CCL20, CCR9, CCR3, CD80, CXCR5, CXCR3,* and *CXCR6*, were predominantly downregulated in normal tissues, particularly in immune and gastrointestinal tissues such as the spleen, stomach, and small intestine. However, in gastric cancer samples (Supplementary Fig. S2B available online at http://bib.oxfordjournals.org/), these hub genes exhibited significant upregulation, suggesting their involvement in tumor-promoting pathways such as inflammation, chemotaxis, and immune cell recruitment. Moreover, the analysis of hub gene expression across different stages of gastric cancer (Supplementary Fig. S2C available online at http://bib.oxfordjournals.org/) demonstrated stage-specific increases in expression, with notable changes observed for *CXCR3* and *CCR3* as the disease progressed from early to advanced stages.

Survival analysis of hub genes in *H. pylori*–associated gastric cancer revealed their prognostic significance, as shown in Supplementary Fig. S3. Supplementary Fig. S3A demonstrates Kaplan–Meier survival analysis, stratifying patients into high-risk and low-risk groups based on the median expression of the combined hub gene signature (*CCR3*, *CCR9*, *CD80*, *CXCR5*, *CXCR6*, *CXCR3*, *CXCL1*, *CCL20*), with high-risk patients showing significantly worse overall survival (log-rank *P* = .032, hazard ratio = 1.46, 95% CI: 1.03–2.07), indicating a 46% higher risk of death compared to the low-risk group. Individual survival curves (Supplementary Fig. S3B) highlighted varied prognostic impacts of high expression levels, with *CCR3* showing a significant association with poorer survival (log-rank *P* = .035, HR = 1.4, 95% CI: 0.98–2.00), while other genes such as *CD80* (*P* = .39, HR = 1.31, 95% CI: 0.92–1.87), CCR9 (*P* = .21, HR = 1.42, 95% CI: 0.99–2.03), *CCL20* (*P* = .33, HR = 0.82, 95% CI: 0.58–1.17), *CXCL1* (*P* = .064, HR = 0.77, 95% CI: 0.54–1.10), CXCR6 (*P* = .23, HR = 0.82, 95% CI: 0.58–1.16), CXCR3 (*P* = .96, HR = 0.82, 95% CI: 0.58–1.16), and CXCR5 (*P* = .77, HR = 1.07, 95% CI: 0.75–1.52) did not reach statistical significance. Heatmap analysis (Supplementary Fig. S3C) illustrated consistently elevated expression of these hub genes in the high-risk group, while boxplots (Supplementary Fig. S3D) confirmed significant expression differences between risk groups, with *CXCL1* (*P* = 3.46e-06, Student’s *t*-test) and *CCL20* (*P* = 4.54e-06, Student’s *t*-test) showing highly significant upregulation in the high-risk group, further validating their prognostic relevance. Together, these findings, supported by the significant survival difference for the combined signature, the significant prognostic impact of *CCR3*, and consistent expression patterns, highlight the dynamic regulation and central roles of hub genes in *H. pylori*–associated gastric cancer progression. Their progressive upregulation during disease stages shows their importance in linking immune dysregulation to tumor development, offering valuable insights into molecular mechanisms and potential avenues for targeted therapies.

### Drug–gene interactions highlight therapeutic opportunities in gastric cancer

To address the question, “Which upregulated genes in *H. pylori*–associated gastric cancer serve as potential therapeutic targets for approved and developmental drugs?”, drug–gene interaction analysis was conducted, as shown in Supplementary Fig. S4 available online at http://bib.oxfordjournals.org/. Interactions between upregulated genes and approved drugs (Supplementary Fig. S4A available online at http://bib.oxfordjournals.org/) identified key targets such as *CD80, CCL3, IL23A,* and *SAA1*, which exhibited high interaction scores with drugs like abatacept, mercaptopurine, and zoledronic acid, suggesting the involvement of these genes in immune modulation and inflammatory processes, highlighting their relevance as therapeutic targets. The top interacting genes and their respective drugs (Supplementary Fig. S4B available online at http://bib.oxfordjournals.org/) further emphasized *SAA1, CD80*, and *TNFRSF11B* as the most promising targets for approved therapies. In addition to approved drugs, interactions with drugs in developmental stages (Supplementary Fig. S4C available online at http://bib.oxfordjournals.org/) revealed promising therapeutic candidates. Genes such as *CCL20, CXCL1*, and *CD80* demonstrated high interaction scores with developmental drugs, including adjuvant, antineoplastic vaccine, and relapladib. The detailed interactions in Supplementary Fig. S4D highlight *CCL20* and *TNFRSF25* as critical players linked to immune/inflammatory pathways, underscore the therapeutic potential of upregulated genes in *H. pylori*–associated gastric cancer, and provide a basis for both drug repurposing and novel drug development, advancing strategies for targeted treatment.

### *In silico* validation of pharmacokinetics of FDA-approved and developmental drugs for *H. pylori*–associated gastric cancer

To validate the therapeutic potential of FDA-approved and developmental drugs identified through drug–gene interaction analysis for targeting hub genes in *H. pylori*–associated gastric cancer, we conducted an *in silico* pharmacokinetic analysis using SwissADME (Supplementary Fig. S5). Supplementary Fig. S5A presents radar plots of physicochemical properties for a subset of drugs that interact with upregulated hub genes, including FDA-approved drugs (e.g. adenosine, mercaptopurine, mitomycin, naproxen sodium, pamidronate, toremifene, zoledronic acid, uridine) and developmental drugs (e.g. ABX-1431, GSK-2879454, KT-109, Picibanil, WWL-123, WWL-70). These plots illustrate key parameters such as lipophilicity (LIPO), size (SIZE), polarity (POLAR), solubility (INSOLU), saturation (INSATU), and flexibility (FLEX), with red shaded areas indicating the optimal range for drug-likeness based on Lipinski’s Rule of Five and other pharmacokinetic criteria. Most drugs, including naproxen sodium, mitomycin, and GSK-2879454, fell within the optimal range across multiple parameters, suggesting favorable drug-likeness for therapeutic application.

Supplementary Fig. S5B illustrates a scatter plot comparing the topological polar surface area (TPSA) and WLOGP (lipophilicity) values of these drugs, with the yellow shaded region representing the optimal range for good oral bioavailability (TPSA <140 Å^2^ and WLOGP between −0.4 and 5.6). The majority of the drugs, including naproxen sodium, mercaptopurine, GSK-2879454, and KT-109, were positioned within this optimal range, indicating their potential for effective oral absorption, a critical factor for clinical applicability in gastric cancer treatment. Drugs were color coded based on predicted pharmacokinetic actions: yellow for blood–brain barrier (BBB) permeability, white for human intestinal absorption (HIA), red for P-glycoprotein substrate (PGP+), and blue for non-P-glycoprotein substrate (PGP−). Notably, FDA-approved drugs like naproxen sodium and mitomycin exhibited favorable HIA and PGP− profiles, while developmental drugs like GSK-2879454 and KT-109 also showed promising bioavailability. However, drugs such as zoledronic acid and pamidronate fell outside the optimal range, suggesting potential limitations in oral bioavailability. These pharmacokinetic profiles validate the feasibility of repurposing these drugs for *H. pylori*–associated gastric cancer, as their favorable drug-likeness and bioavailability support their potential to effectively target hub genes like *CXCL1, CCL20,* and *IL12B*, which drive inflammation and immune dysregulation.

### Transcriptomic data integration highlights common DEGs and their role in disease progression

**Figure 7:**
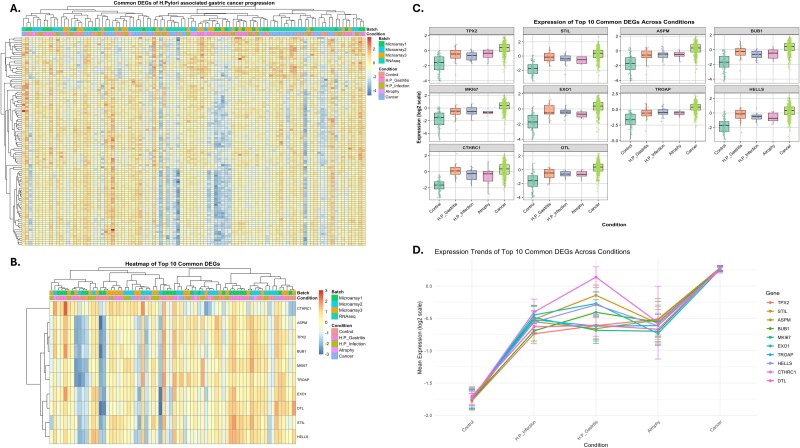
Integration of microarray and RNA-seq datasets using ComBat to analyze common DEGs across *H. pylori*–associated gastric cancer progression. (A) The heatmap of all common DEGs highlights distinct expression patterns across conditions, from infection to cancer. (B) Focused heatmap of the top 10 common DEGs (e.g. *TPX2*, *MKI67*, and *EXO1*) shows progressive changes in expression. (C) Boxplots illustrate significant shifts in DEG expression across conditions. (D) Line plots depict consistent trends of upregulated expression from infection to cancer, reflecting the roles of these genes in immune response, cell proliferation, and tumor progression.

To address the question, how do common DEGs reflect the progression of *H. pylori*–associated gastric cancer from infection to malignancy? Our gene expression data from microarray datasets and RNA-seq data were integrated using ComBat to correct batch effects. This integration enabled the identification of consistent expression patterns of common DEGs across stages of disease progression, providing insights into their roles in linking inflammation-driven infection to cancer development. The heatmap of all common DEGs (Fig. 7A) revealed distinct clustering patterns, reflecting transcriptional changes corresponding to the stages of *H. pylori* infection, gastritis, atrophy, and gastric cancer. Hierarchical clustering displayed clear separation between conditions, emphasizing the stage-specific molecular shifts that occur as the disease progresses. The focused heatmap of the top 10 DEGs (Fig. 7B) highlighted genes such as *TPX2*, *MKI67*, *EXO1*, and *CTHRC1*, which showed progressive upregulation from infection to cancer. These genes are involved in critical processes, including cell cycle regulation (*TPX2*, *MKI67*), DNA repair (*EXO1*), and extracellular matrix remodeling (*CTHRC1*), highlighting their importance in the shift from chronic inflammation to malignancy. Moreover, boxplots (Fig. 7C) further confirmed the dynamic changes in the expression levels of these DEGs across conditions, demonstrating significant transcriptional shifts that align with disease progression. The line plot of expression trends (Fig. 7D) revealed a consistent increase in expression from control to cancer, with notable peaks during gastritis and atrophy stages.

To further validate the effectiveness of the ComBat integration in harmonizing the transcriptomic datasets while preserving biologically relevant signals, we conducted PCA clustering both before and after batch correction (Supplementary Fig. S6). As shown in Supplementary Fig. S6A, the 3D-PCA clustering prior to ComBat integration showed a distinct separation of samples from the four datasets GSE27411 (atrophy versus control, *H. pylori* infection), GSE60427 (control versus *H. pylori* gastritis), GSE233973 (control versus *H. pylori* gastritis), and TCGA-STAD (primary tumor versus solid tissue normal) primarily driven by batch effects, with each dataset forming separate clusters despite shared biological conditions. After applying ComBat integration, the 3D-PCA clustering (Supplementary Fig. S6B) demonstrated a significant reduction in batch-driven separation, with samples clustering more closely based on biological conditions (atrophy, infection, gastritis, cancer) than their original datasets. This integration maintained the stage-specific molecular signatures crucial for understanding disease progression, as indicated by the consistent clustering of conditions across datasets. The 2D-PCA plot (Supplementary Fig. S6C) further confirmed these findings, with PC1 and PC2 explaining 27.0% and 12.7% of the variance, respectively, and demonstrating improved overlap of samples from different batches while preserving the separation based on biological conditions. These PCA results illustrate the robustness of the ComBat integration in enabling the identification of common DEGs and their expression patterns across the stages of *H. pylori*–associated gastric cancer progression, as further explored in the heatmap and expression analyses (Fig. 7).

## Discussion

Gastric cancer is a malignant tumor with elevated mortality rates, but due to the lack of therapeutic biomarkers, it is diagnosed in a late stage and has a poor prognosis; therefore, it is considered a fatal cancer with a high mortality rate [26]. It is known that infection with *H. pylori* causes chronic active gastritis, which causes a chronic inflammatory response considered a risk factor for gastric cancer [27] by an inflammatory process which was defined by Correa [28, 29]. In this study, we highlighted the intersections of DEGs across datasets representing *H. pylori* infection, gastritis, and atrophy, revealing 217 DEGs shared among the three *H. pylori* stages. Furthermore, an overlap of 43 DEGs was identified across the TCGA-STAD dataset and the three *H. pylori* stages, representing genes consistently involved in disease progression, suggesting that these genes may contribute to both *H. pylori* infection and the development or progression of gastric cancer, identifying them as potential candidates for further research as key biomarkers in the connection between *H. pylori* infection and gastric cancer.

Our integrative transcriptomic analysis of *H. pylori*–associated gastric cancer has identified hub genes such as *CXCL1*, *CCL20*, *IL12B*, and *TPX2* as promising biomarkers and therapeutic targets, providing valuable insights into the molecular mechanisms linking *H. pylori* infection to gastric carcinogenesis. While these molecular markers play a central role in immune dysregulation, cell cycle regulation, and tumor development, we recognize that gastric cancer is a multifactorial disease influenced by clinical factors (e.g. tumor stage, patient age), environmental factors (e.g. diet, smoking), and microbial factors (e.g. co-infections, microbial dysbiosis). Therefore, the identified hub genes should be considered alongside these other factors to enhance disease identification, risk stratification, and personalized treatment strategies. Future studies integrating these molecular markers with clinical, environmental, and microbial data will be crucial to fully elucidate their role in *H. pylori*–associated gastric cancer and to develop comprehensive diagnostic and therapeutic approaches, paving the way for precision medicine in this disease.

The integration of GO enrichment and network analysis highlighted key genes, forming highly connected hubs in pathways related to chemokine-mediated signaling and myeloid leukocyte activation as well as *KRAS* signaling up, *mTORC1* signaling, and *TNFA* signaling via *NFκB*, which play a significant role in mediating inflammation and immune cell recruitment, marking the early response to *H. pylori* infection and the transition to chronic inflammation in gastritis, reflecting metabolic reprogramming and extracellular matrix interactions characteristic of the transition to gastric atrophy and cancer [30–32]. Moreover, it can provide insights into the molecular mechanisms of *H. pylori*–associated disease progression and highlight potential therapeutic targets within the enriched pathways and gene networks.

It is clear that *H. pylori* uses its virulence factors to trigger neutrophil influx and activation, which is central to its ability to cause disease [33]. In keeping with its ability to thrive in a neutrophil-rich environment, several bacterial and host factors act in concert for robust recruitment of neutrophils to the infected human gastric mucosa including the neutrophil-activating protein [34], which is one of the factors resulting in *H. pylori–*induced neutrophil migration [35]. Also, it triggers a strong immune response through its lipopolysaccharide (*LPS*), a key component of its outer membrane, which binds to toll-like receptors (*TLR4*) on gastric epithelial and immune cells [36, 37]. This interaction activates the *NF-κB* signaling pathway, leading to the production of pro-inflammatory cytokines, particularly *TNFα* which is a carcinogenic and inflammatory factor related to gastric carcinogenesis and *IL-1β*, that amplify the inflammatory response [32, 38, 39]. *TNF-α*-inducing protein (*tipα*) gene family, comprising *H. pylori* membrane protein 1 and tipα, has been identified as a tumor promoter, contributing to *H. pylori* carcinogenicity. *Tipα* secreted from *H. pylori* stimulates gastric cancer development by inducing *TNF-α*, an endogenous tumor promoter. *TNFα* further enhances *NF-κB* activity in a positive feedback loop, promoting chronic inflammation. This enhancement activation induces the production of reactive oxygen species, causing cellular damage and contributing to the development of gastritis. Over time, chronic inflammation leads to cellular dysregulation, including the promotion of cell survival, proliferation, and resistance to apoptosis [40]. These effects, along with oxidative stress and disruptions in signaling pathways like *PI3K/AKT* and *JAK/STAT*, increase the risk of gastric epithelial dysplasia and the progression to gastric cancer [37, 41]. During chronic infection, chemotaxis helps maintain bacterial populations and modulates the host immune response. Johnson and Ottemann [42] suggested that according to its importance, it is an attractive target for future treatments against *H. pylori* infections. It was found that the type of signals found for *H. pylori* chemotaxis implies that this microbe can use them to localize relative to the gastric epithelium and potentially away from other bacteria, thus leading to finding the optimal place for stable long-term colonization [43], which is followed by targeting gastric injury sites, based on quantifying *H. pylori* near sites of acetic acid damage, a model system for creating ulcers [44]. This illustrates a comprehensive view of disease progression, beginning with the onset of inflammation during the early stages of *H. pylori* infection and gastritis, advancing to immune dysregulation and epithelial damage during the development of atrophic gastritis, and ultimately culminating in metabolic reprogramming and enhanced extracellular matrix interactions characteristic of gastric cancer; moreover, it can provide insights into the molecular mechanisms of *H. pylori*–associated disease progression and highlight potential therapeutic targets within the enriched pathways and gene networks.

PPI network analysis highlighted eight key hub genes (*CD80*, *CCR9*, *CXCR3*, *CXCR5*, *CXCR6*, *CCR3*, *CCL20*, and *CXCL1*), revealing their pivotal roles in gastric cancer and *H. pylori* infection. Muhammad *et al.* [45] reported *CXCL1*’s overexpression in both gastric cancer tissues and *H. pylori*–infected cells, attributing this to NF-κB activation and interleukin-32 upregulation, which drives neutrophil infiltration into the gastric mucosa. Similarly, the *CCL20/CCR6* axis plays a crucial role in regulatory T-cell migration to infected gastric mucosa [46]. *Helicobacter pylori* further promotes chemokine dysregulation by secreting *TNF-α*-inducing protein (*Tip-α*), which elevates *CXCL1* and other chemokines like *CCL20* and *CXCL10*, fostering chronic inflammation and immune cell recruitment [47, 48]. These chemokine-mediated pathways not only sustain inflammation but also contribute to tumor-promoting environments in the gastric mucosa, underscoring the intricate interplay between infection and cancer progression.

Also, *CXCL13* and *CXCR5* are implicated in tumor growth and metastasis, with *CXCL13* serving as a predictive marker for chemotherapy response in advanced gastric cancer patients [49]. *CXCR3* exhibits a dual role, either inhibiting tumor growth by blocking angiogenesis or promoting metastasis through chemotaxis [50]. *CXCR6* has been linked to poor prognosis in gastric cancer and facilitates metastasis via epithelial–mesenchymal transition, with *H. pylori* infection exacerbating this process by increasing gastric cancer risk up to six-fold [51, 52]. However, blocking *CXCR6* has demonstrated potential in reducing gastric cancer cell migration and invasion, suggesting its viability as a therapeutic target. Together, these findings illuminate the role of chemokine signaling in *H. pylori*–induced inflammation and gastric cancer progression, offering avenues for targeted interventions.

Our integrative transcriptome analysis, supported by Supplementary Fig. S2, demonstrates that hub genes such as *CXCL1*, *CCL20*, and *CXCR3* are consistently dysregulated across *H. pylori* infection, gastritis, atrophy, and gastric cancer stages, including early stages (I and II), supporting their potential as biomarkers for disease progression and early detection. However, the overrepresentation of late-stage tumors in TCGA-STAD needs further validation. Future studies should prioritize independent early-stage gastric cancer cohorts and prospective patient samples to evaluate the diagnostic and prognostic utility of these biomarkers for early detection.

We have identified hub genes such as *CXCL1*, *CCL20*, and *IL12B*, which are enriched in inflammatory pathways like chemotaxis and cytokine signaling. This raises the possibility of overlap with general inflammatory responses due to *H. pylori*’s role in chronic inflammation. However, the stage-specific expression patterns of these genes across *H. pylori* infection, gastritis, atrophy, and gastric cancer, along with their progressive upregulation in cancer-specific processes like immune dysregulation and tumor development, suggest that they are linked to the molecular progression of *H. pylori*–associated gastric cancer rather than being only markers of inflammation. Identifying hub genes such as *TPX2*, *MKI67*, and *EXO1*, which are involved in cell cycle regulation and DNA repair, further supports the cancer-specific relevance of our biomarkers. Their interaction with cancer-relevant drugs like abatacept and zoledronic acid also highlights their potential as therapeutic targets. To confirm the specificity of these biomarkers, future studies should compare their expression profiles in *H. pylori*–associated gastric cancer with other inflammatory conditions, such as inflammatory gastric disease or non–*H. pylori*-related gastritis to establish their unique contributions to gastric carcinogenesis and enhance their clinical utility. While biomarkers like *CXCL1* and *CCL20* are linked to *H. pylori*–driven inflammation, their elevation in *H. pylori* gastric cancer samples indicates broader relevance to gastric cancer pathology. Future studies should compare these biomarkers in *H. pylori*–positive versus negative gastric cancer cohorts and other cancer types to determine their specificity and translational potential.

Drug–gene interaction analysis was conducted, at which interactions between upregulated genes and approved drugs identified key targets that exhibited high interaction scores with drugs like abatacept, mercaptopurine, and zoledronic acid. These interactions suggest the involvement of these genes in immune modulation and inflammatory processes, highlighting their relevance as therapeutic targets. In addition, interactions with drugs in developmental stages revealed promising therapeutic candidates. Genes demonstrated high interaction scores with developmental drugs, including adjuvant, antineoplastic vaccine, and relapladib, suggesting that these genes play significant roles in immune modulation and inflammatory pathways, confirming their value as therapeutic targets, and highlighting the potential for repurposing these approved drugs to address dysregulated pathways in gastric cancer [53, 54]. Furthermore, interactions with drugs in developmental stages emphasize the opportunity to explore novel therapeutic candidates. These developmental drugs, targeting key genes, present a promising avenue for innovative treatments tailored to the molecular landscape of *H. pylori*–induced gastric cancer, making our results lay a strong foundation for advancing personalized treatment strategies through both drug repurposing and the development of novel therapies, ultimately improving outcomes for patients with this disease.

Several identified hub genes, including *CXCL1*, *CCL20*, *IL12B*, *STAT4*, and *CD80*, are involved in chemokine signaling, immune modulation, and inflammation, which can promote *H. pylori*–mediated gastric cancer. Notably, abatacept (a CTLA-4-Ig fusion protein that binds *CD80/CD86*) has demonstrated the ability to modulate T-cell activation and has shown antitumor activity in immunologically active cancers. Its relevance to gastric cancer is supported by evidence that immune checkpoint modulation may be beneficial in inflammation-associated tumors such as those driven by *H. pylori*. A compelling case study by Giulia Angelino *et al.* [55] described the successful use of abatacept in a 17-year-old patient with *CTLA-4* deficiency, who presented with gastric cancer along with severe immune dysregulation, including early-onset inflammatory bowel disease, type 1 diabetes, and polyautoimmunity. Genetic testing confirmed *CTLA-4* deficiency, and following subtotal gastrectomy, abatacept treatment led to sustained disease control, with the patient remaining stable and symptom-free for 18 months. This case highlights the potential of abatacept to therapeutically modulate immune-driven pathways involved in gastric cancer, especially in patients with underlying immune dysregulation. It further highlights the promise of immune checkpoint-based therapies in managing *H. pylori*–associated gastric cancer.

In addition to abatacept, zoledronic acid, a bisphosphonate commonly used to manage bone metastases, has shown promising therapeutic potential in gastric cancer due to its anti-angiogenic and immunomodulatory properties. Evidence supporting its relevance comes from a clinical study by Fatma Paksoy Turkoz *et al*. [56], which analyzed 176 gastric cancer patients with bone metastases. The study found that treatment with zoledronic acid was significantly associated with prolonged survival, identifying it as an independent prognostic factor in multivariate analysis (*P* < .001). Patients who received zoledronic acid had improved outcomes, particularly those with isolated bone metastases or well-differentiated tumors. These findings suggest that beyond its established role in bone health, zoledronic acid may exert antitumor effects that could be beneficial in gastric cancer, especially in cases where the disease progression involves inflammation, metastasis, and immune modulation. Given that some of the upregulated hub genes in our study, such as *SAA1* and *TNFRSF11B* (Fig. 6B), are involved in inflammatory and immune-related pathways, targeting them with zoledronic acid may provide a repurposing opportunity to disrupt tumor-supportive signaling in *H. pylori*–associated gastric cancer.

In addition to approved drugs like abatacept and zoledronic acid, our study identified some developmental drugs, including adjuvant therapies, antineoplastic vaccines, and relapladib, that target upregulated hub genes involved in immune modulation and chronic inflammation, key mechanisms in *H. pylori*–associated gastric carcinogenesis. The relevance of developmental therapies in gastric cancer is supported by clinical evidence from the CLASSIC trial, a landmark phase III controlled study (NCT00411229), which demonstrated that adjuvant chemotherapy with capecitabine plus oxaliplatin significantly improved 3-year disease-free survival in patients with stage II–IIIB gastric cancer who underwent curative D2 gastrectomy [53, 57]. The addition of adjuvant treatment reduced the risk of recurrence by 44% compared to surgery alone (HR: 0.56; *P* < .0001), establishing it as a standard option in advanced gastric cancer management. These findings confirm the value of developmental and adjuvant agents in enhancing clinical outcomes, particularly in inflammation-driven subtypes such as *H. pylori*–induced malignancies. Moreover, several genes identified in our analysis, including *IL23A* and *CCL20*, are targets of investigational immunomodulatory drugs such as antineoplastic vaccines and relapladib, providing promising avenues for personalized treatment. Thus, integrating transcriptomic insights with evidence-based adjuvant therapies highlights a rationale for repurposing and further evaluating these developmental drugs in the context of *H. pylori*–associated gastric cancer.

In recent years, several studies have utilized the integrative approach to discover unknown genes associated with *H. pylori*–related gastric cancer, identify novel gene signatures, and explore potential therapeutic targets for *H. pylori*–induced gastric disease [12, 13, 25, 58, 59]. By employing ComBat to correct for batch effects in our study and focusing on the top DEGs, we highlighted genes such as *TPX2*, *MKI67*, *EXO1*, and *CTHRC* 1, which exhibited progressive upregulation from infection to cancer. These genes are involved in cell cycle regulation, DNA repair, and extracellular matrix remodeling, highlighting their significance in the transition from chronic inflammation to malignancy. *TPX2* and *MKI67* have been recognized as prominent genes in gastric cancer [10, 60], with elevated *TPX2* expression linked to tumor progression and poor survival outcomes, suggesting its potential role as a biomarker for adverse prognosis or a target for future therapeutic strategies in gastric cancer [60, 61]. Other integrated analyses have identified *EXO 1*, *DTL*, *KIF 14*, and *TRIP13* as significant genes that could serve as potential diagnostic biomarkers related to gastric cancer [62]. *CTHRC1* may assist tumor cells in evading detection and destruction by the immune system, potentially through mechanisms such as suppressing immune responses, reducing immune cell infiltration into the tumor, or modifying the tumor microenvironment to promote tumor growth [63]. This highlights the functions of these genes in immune activation, epithelial cell proliferation, and stromal remodeling, demonstrating how common DEGs act as molecular markers of disease progression and emphasizing their roles in the biological processes driving *H. pylori*–associated gastric cancer. The consistent upregulation of key genes across various stages suggests their potential as therapeutic targets and biomarkers, providing a framework for understanding the molecular continuum of infection-induced malignancy. While our study attributes the identified hub genes (e.g. *CXCL 1*, *CCL 20*, *IL 12 B*) to *H. pylori*–associated gastric cancer based on their progressive dysregulation across *H. pylori*–related disease stages, we acknowledge that other Gram-negative bacteria, such as *Fusobacterium nucleatum*, may also contribute to similar inflammatory and oncogenic pathways. Future studies should incorporate comprehensive microbial profiling and comparative analyses with other Gram-negative bacteria to validate the specificity of these hub genes to *H. pylori*–driven gastric carcinogenesis.

Herein, our analysis identifies gene expression changes across *H. pylori*–associated disease progression, with hub genes like *CXCL1* and *TPX2* showing early bacterial effects and later tumor-driven changes. The elevated expression of these markers in gastric cancer indicates their potential as biomarkers of *H. pylori*–driven gastric cancer progression. Future studies should include larger cohorts of *H. pylori*–positive and *H. pylori*–negative gastric cancer samples with detailed annotations to further delineate the specific contributions of *H. pylori* infection versus tumor progression. However, validation in independent *H. pylori*–associated gastric cancer cohorts or through experimental methods, such as qPCR or immunohistochemistry, is needed to confirm their clinical utility.

In summary, these findings show the power of the integration approach to identify key hub genes driving the progression from *H. pylori* infection to gastric cancer. The highlighted genes, including *TPX2*, *MKI67*, *EXO1*, and *CTHRC1*, not only shed light on biological processes such as cell cycle regulation, DNA repair, and immune evasion but also hold promise as potential biomarkers for early diagnosis, prognostic evaluation, and therapeutic targeting in gastric cancer. These insights pave the way for future research to validate and explore the clinical applications of these gene signatures in managing *H. pylori*–induced gastric malignancies.

## Conclusion

Our study successfully identified key hub genes, functional modules, and pathways involved in *H. pylori*–associated gastric cancer, enhancing our understanding of the molecular mechanisms underlying this disease. Our network analysis revealed eight hub genes showing significant associations with survival outcomes based on Cox proportional hazards analysis. We highlighted genes, including *TPX2*, *MKI67*, *EXO1*, and *CTHRC1*, as potential biomarkers for early diagnosis, prognostic evaluation, and therapeutic targeting in gastric cancer. These insights could potentially pave the way for future research to validate and explore the clinical applications of these gene signatures in managing *H. pylori*–induced gastric malignancies.

**Key Points**

- The integration of RNA-seq and microarray data for transcriptome analysis revealed key differentially expressed genes (DEGs) associated with *H. pylori*–related gastric cancer progression.
- Distinct clustering patterns and stage-specific transcriptional changes were identified across *H. pylori* infection, gastritis, atrophy, and gastric cancer.
- Hub genes such as *TPX2, MKI67, EXO1*, and *CTHRC1* showed progressive upregulation, implicating roles in cell cycle regulation, DNA repair, and extracellular matrix remodeling.
- Network analysis highlighted immune-related hub genes (*CXCL1, CCL20, IL12B*, and *STAT4*) involved in chemotaxis, leukocyte migration, and cytokine signaling, linking immune dysregulation to tumor development.
- Drug–gene interaction analysis identified potential therapeutic targets, with hub genes interacting with approved and developmental drugs, suggesting precision medicine opportunities for *H. pylori*–driven gastric cancer.

## Supplementary Material

sup_fig_1_bbaf241

sup_fig_2_bbaf241

sup_fig_3_bbaf241

sup_fig_4_bbaf241

sup_fig_5_bbaf241

sup_fig_6_bbaf241

Supplementary_information_H_bbaf241_Pylori_bbaf241

FinalHPyloriGraphs_bbaf241

## Data Availability

The code and processed data used in this study are publicly available on GitHub at the following repository: https://github.com/asmaamohamedreda/HPyloriGC.

## Author Biographies

**Dr Sara Hussein Mohamed** holds a Ph.D. in Microbiology. Her research focuses on applied microbiology, infectious diseases, and bioinformatics. She publishes in high-impact journals and serves as a scientific reviewer in the field of microbiology.

**Dr Mohamed Hamed** is a professor of biomedical informatics at the GUC and his research interests include precision oncology, biological data integration, drug combination predictions, radiogenomics, and computational immunology.

**Dr Fadhl Alakwaa** is a computational biologist at the University of Michigan. His research focuses on multi-omics integration, machine learning, and precision medicine in kidney disease and cancer.

**Dr Asmaa Reda** is a computational biologist and bioinformatician at the Faculty of Science, Benha University. She specializes in multi-omics data integration, drug design, and single-cell genomics, with a focus on precision medicine and therapeutic biomarker discovery.
