# Supplementary figures and images for: Multi-omics analysis of Helicobacter pylori–associated gastric cancer identifies hub genes as a novel therapeutic biomarker
Source: Brief Bioinform. 2025 May 30;26(3):bbaf241. doi: 10.1093/bib/bbaf241 (PMC12123523; doi:10.1093/bib/bbaf241)

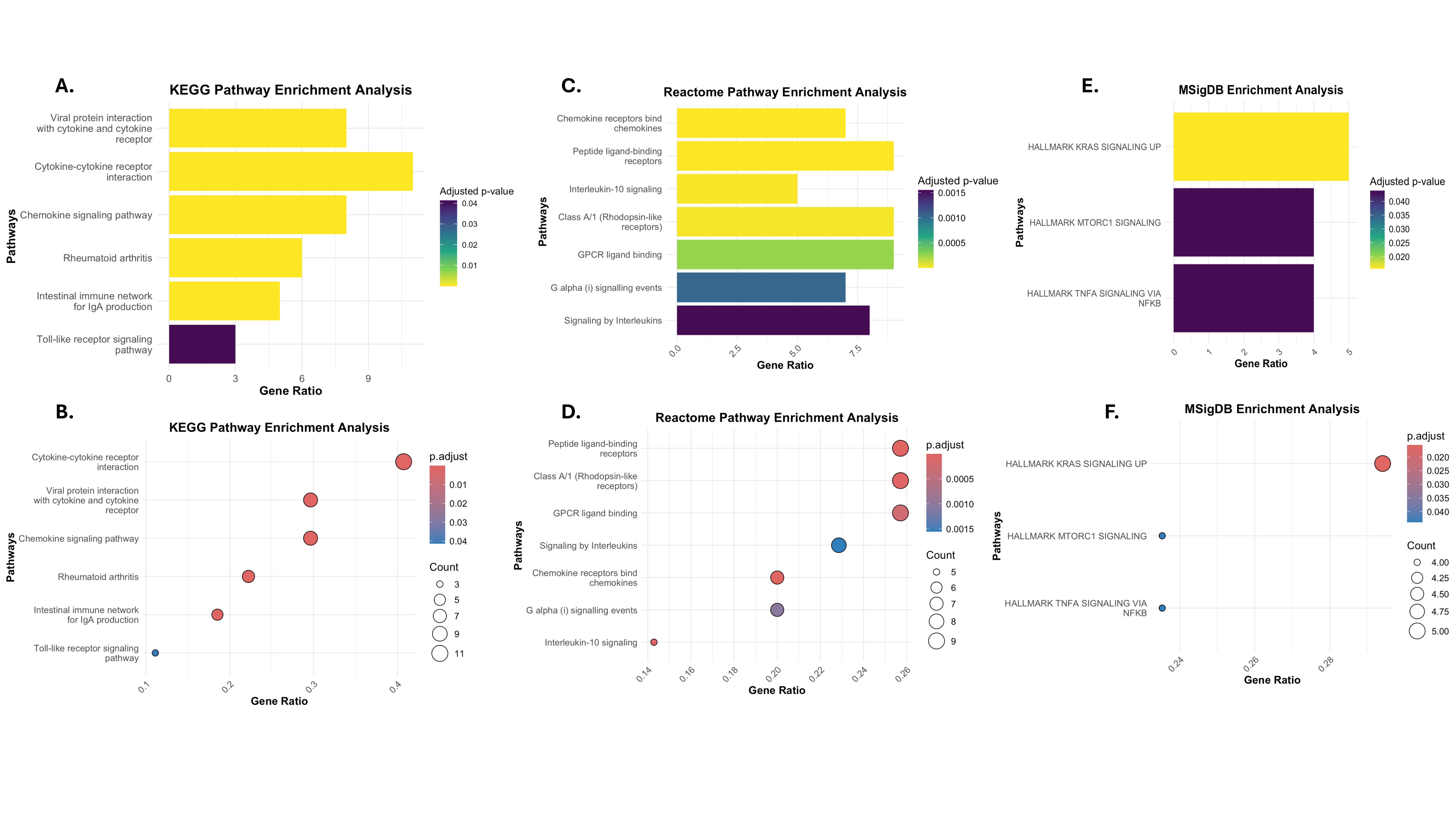

Supplement: sup_fig_1_bbaf241 [file sup_fig_1_bbaf241.jpeg]

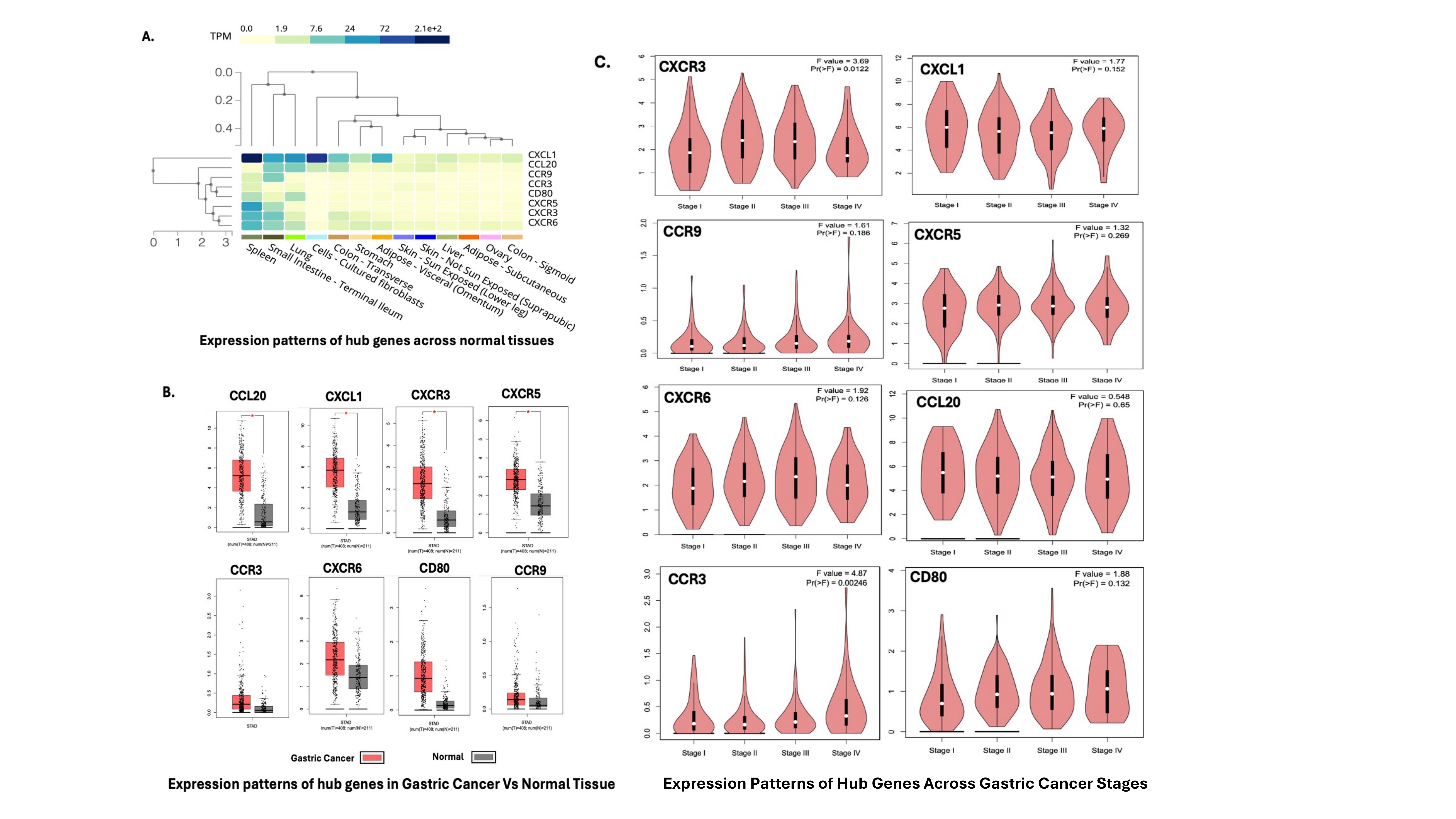

Supplement: sup_fig_2_bbaf241 [file sup_fig_2_bbaf241.jpeg]

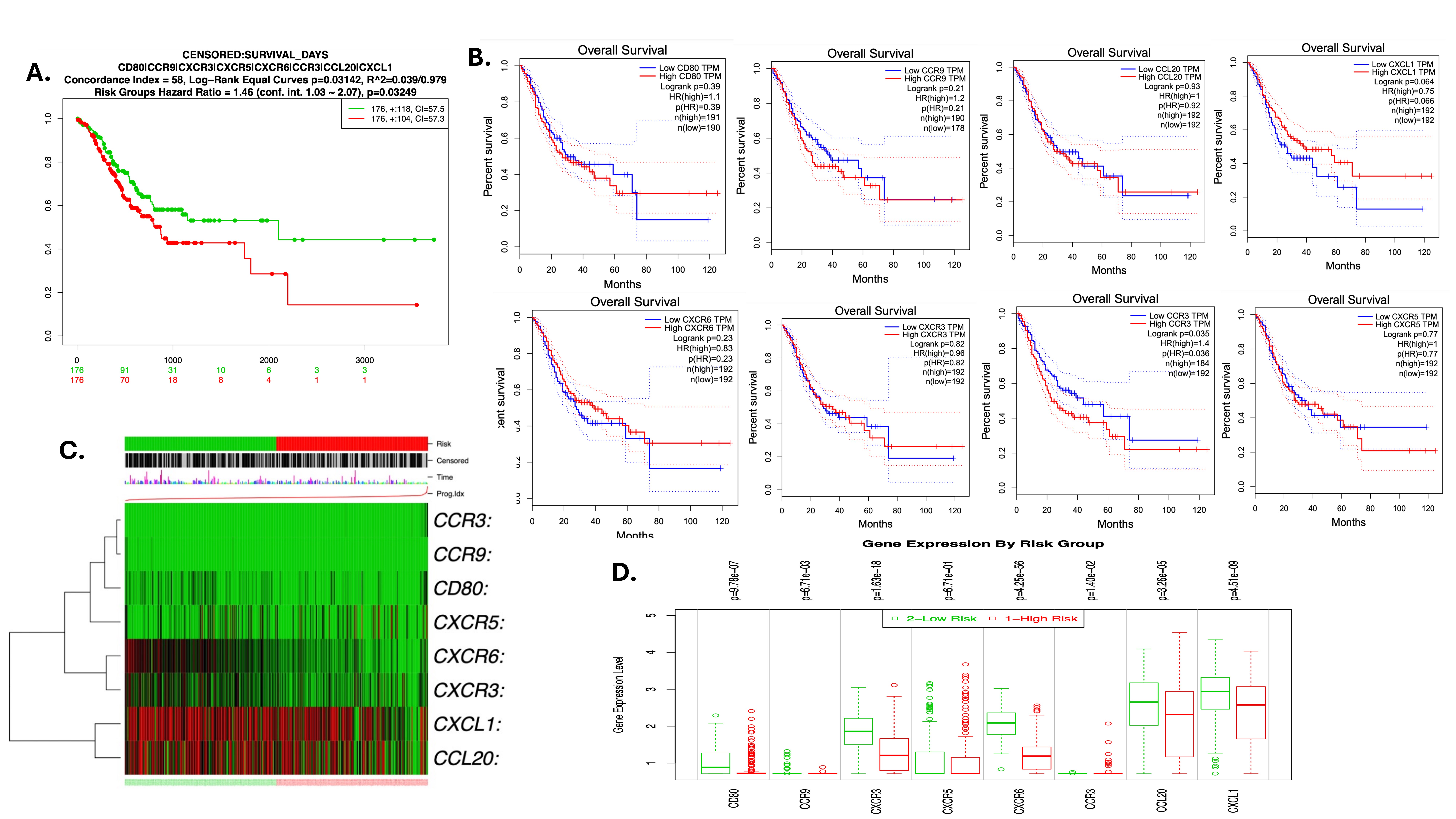

Supplement: sup_fig_3_bbaf241 [file sup_fig_3_bbaf241.jpeg]

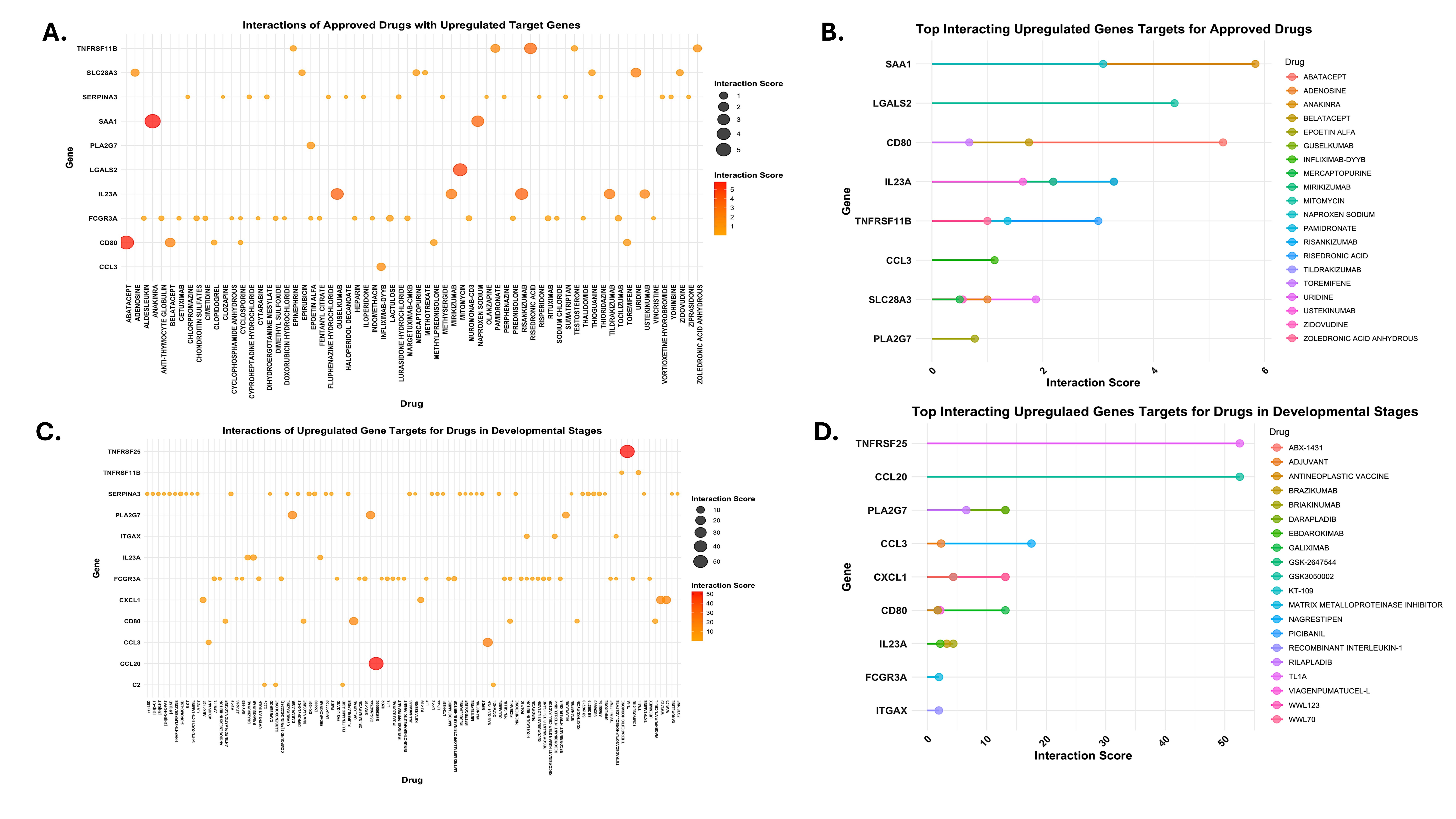

Supplement: sup_fig_4_bbaf241 [file sup_fig_4_bbaf241.jpeg]

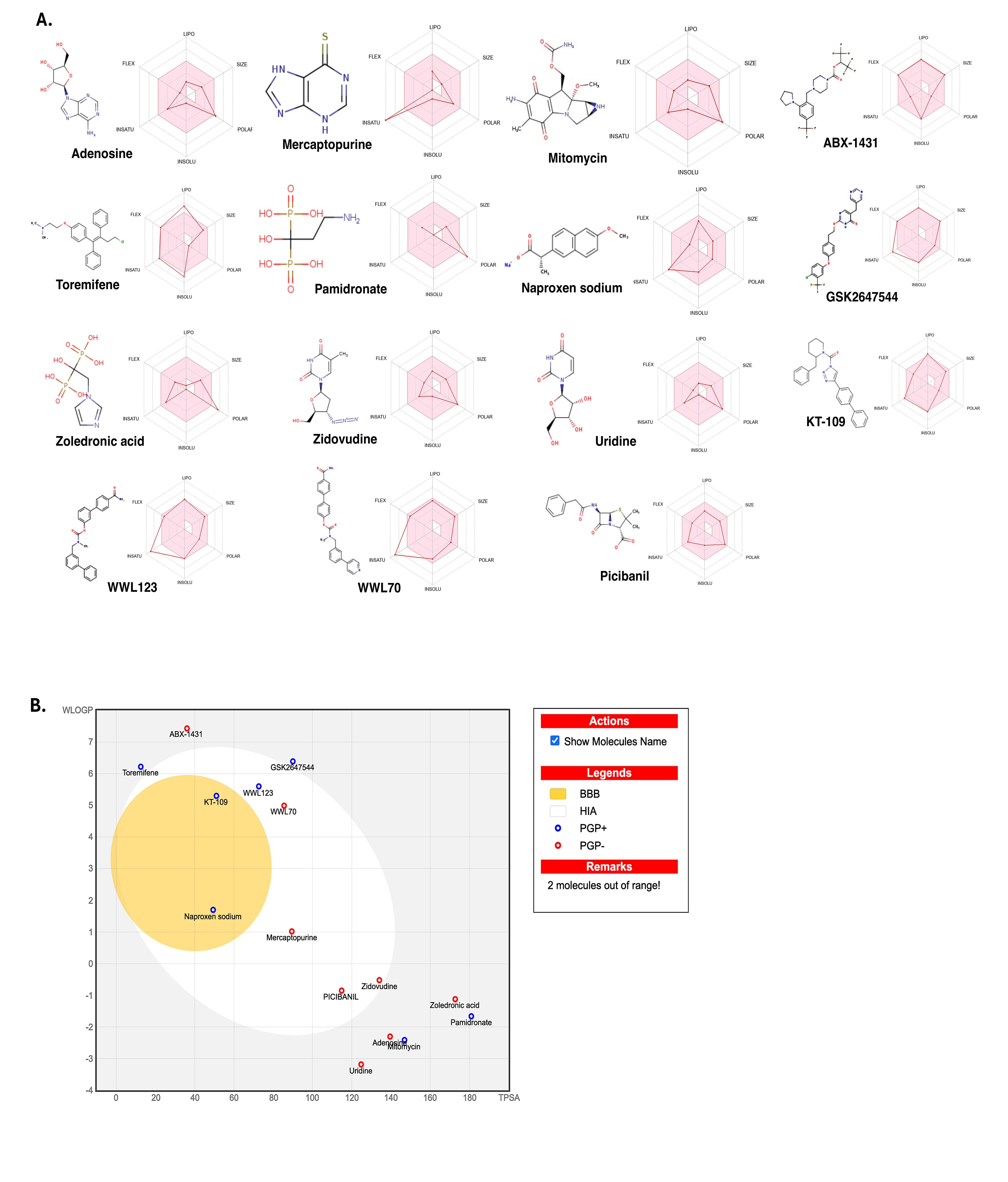

Supplement: sup_fig_5_bbaf241 [file sup_fig_5_bbaf241.jpeg]

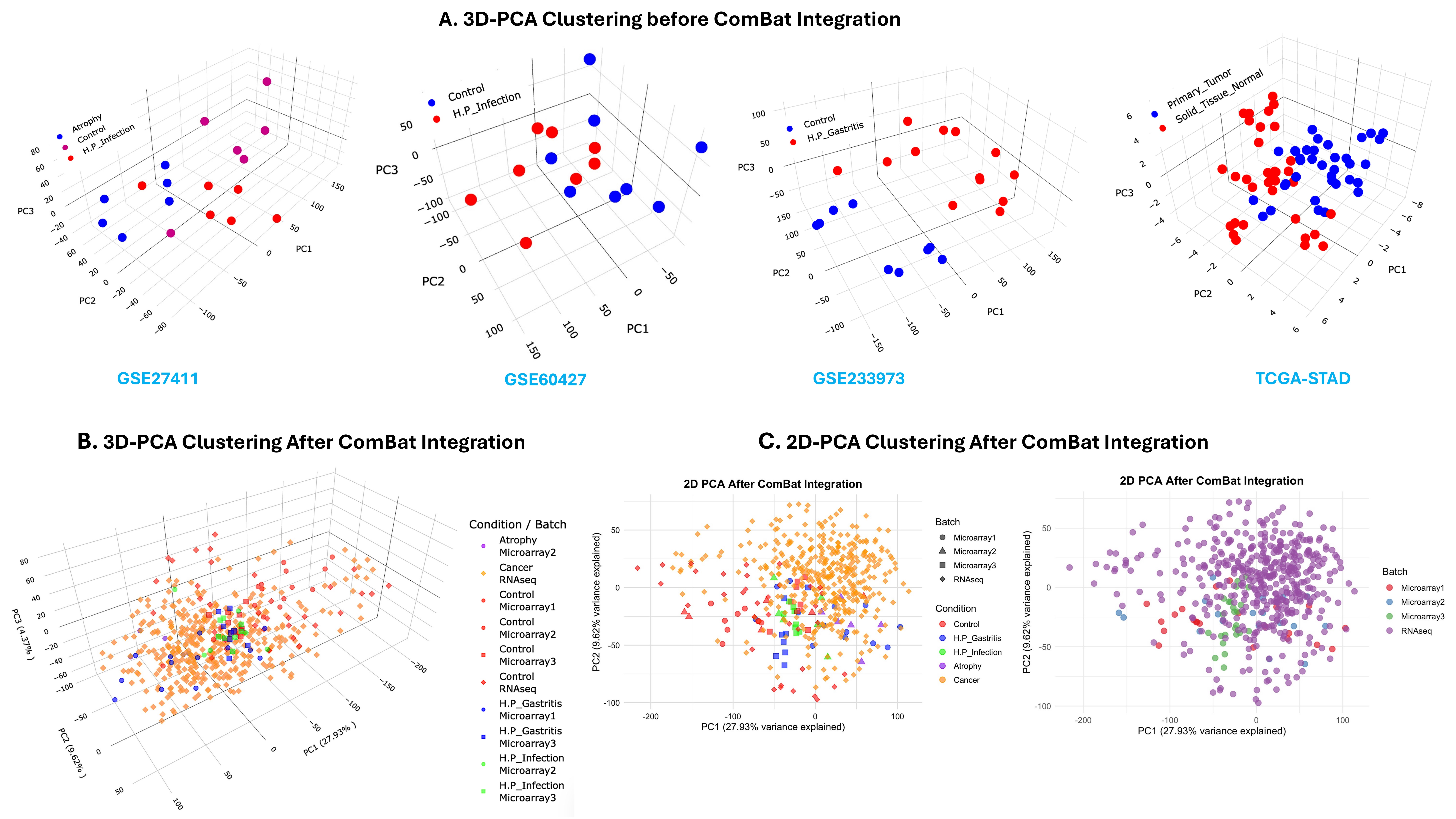

Supplement: sup_fig_6_bbaf241 [file sup_fig_6_bbaf241.jpeg]
