# Supplementary material for: Multi-omics analysis of Helicobacter pylori–associated gastric cancer identifies hub genes as a novel therapeutic biomarker
Source: Brief Bioinform. 2025 May 30;26(3):bbaf241. doi: 10.1093/bib/bbaf241 (PMC12123523; doi:10.1093/bib/bbaf241)
Supplement: Supplementary_information_H_bbaf241_Pylori_bbaf241 [file supplementary_information_h_bbaf241_pylori_bbaf241.docx]

Supplementary Information

**Supplementary Table. 1**. Complete dataset descriptions of *H.Pylori* Vs Control


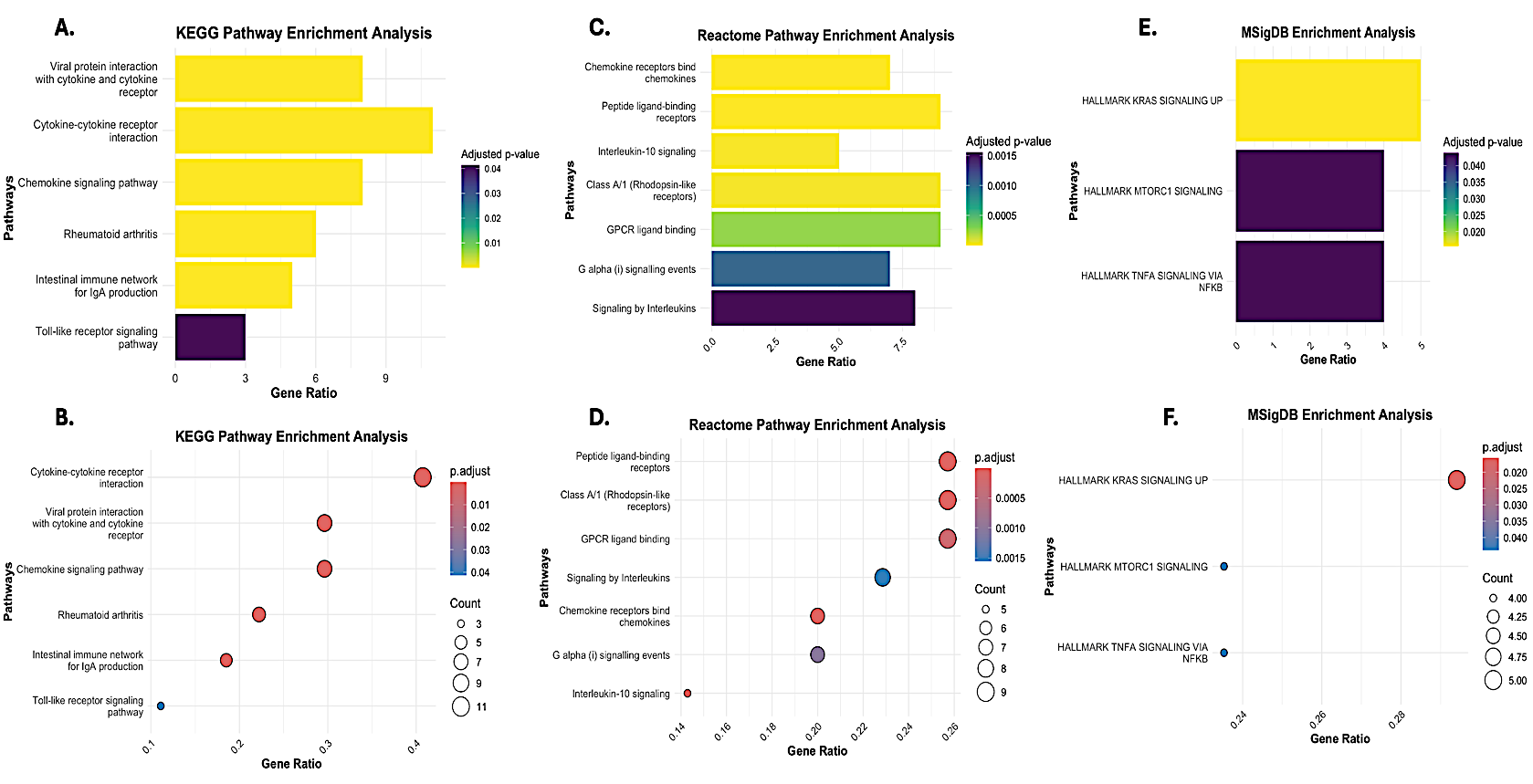


**Figure 1 Supplementary** illustrates pathway enrichment analysis highlighting key signaling mechanisms involved in ***H*. *pylori*-associated gastric cancer progression. A-B:** KEGG pathway enrichment identifies critical immune and inflammatory pathways, including **cytokine-cytokine receptor interaction, chemokine signaling,** and **Toll-like receptor signaling**, essential for immune cell recruitment and inflammation. **C-D:** Reactome enrichment reveals pathways such as **chemokine receptor binding, *GPCR* ligand binding, and interleukin-10 signaling, e**mphasizing immune modulation and signaling cascades. **E-F:** MSigDB analysis highlights hallmark pathways, including ***KRAS* signaling up, *mTORC1* signaling,** and ***TNFA* signaling via *NFκB*,** which are implicated in cell proliferation, survival, and chronic inflammation. These findings provide insights into the molecular pathways driving disease progression and highlight potential therapeutic targets.

| Dataset | Experiment type | Conditions Covered | | Total No. of Samples | Accession |
| --- | --- | --- | --- | --- | --- |
|  |  | control | Case: Infection, Gastritis, Atrophy, Tumor |  |  |
| GSE27411 | Expression profiling by array | 6 | Atrophy: 6  Infection: 6 | 18 | GEO |
| GSE60427 | Expression profiling by array | 8 | Gastritis: 8  Infection: 8 | 24 | GEO |
| GSE233973 | Expression profiling by array | 9 | Gastritis: 13 | 22 | GEO |
| TCGA-STAD | RNA-seq | 36 | Gastric Cancer: 412 | 448 | TCGA |


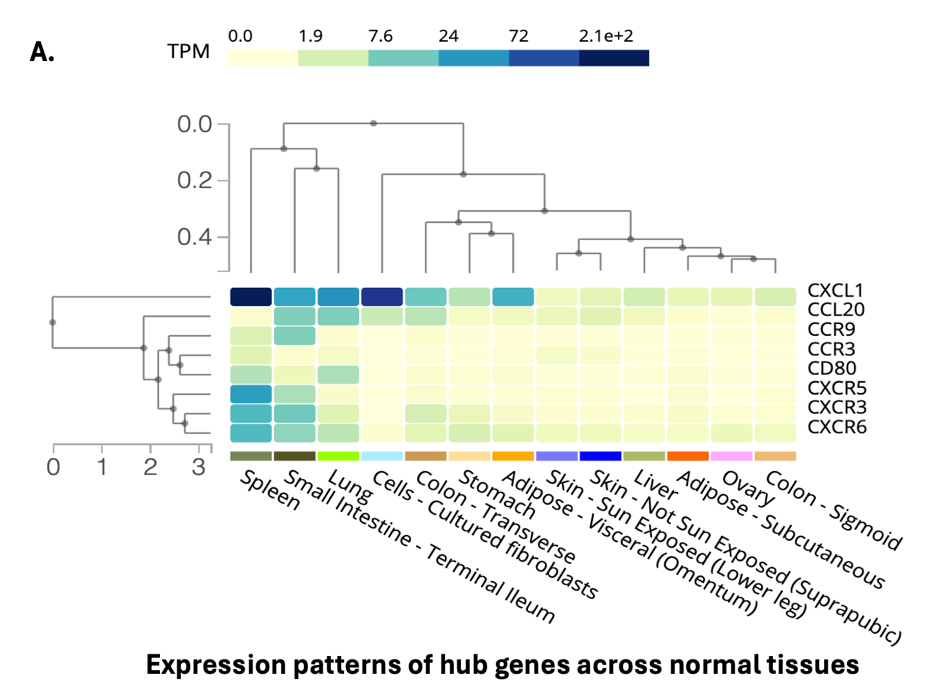

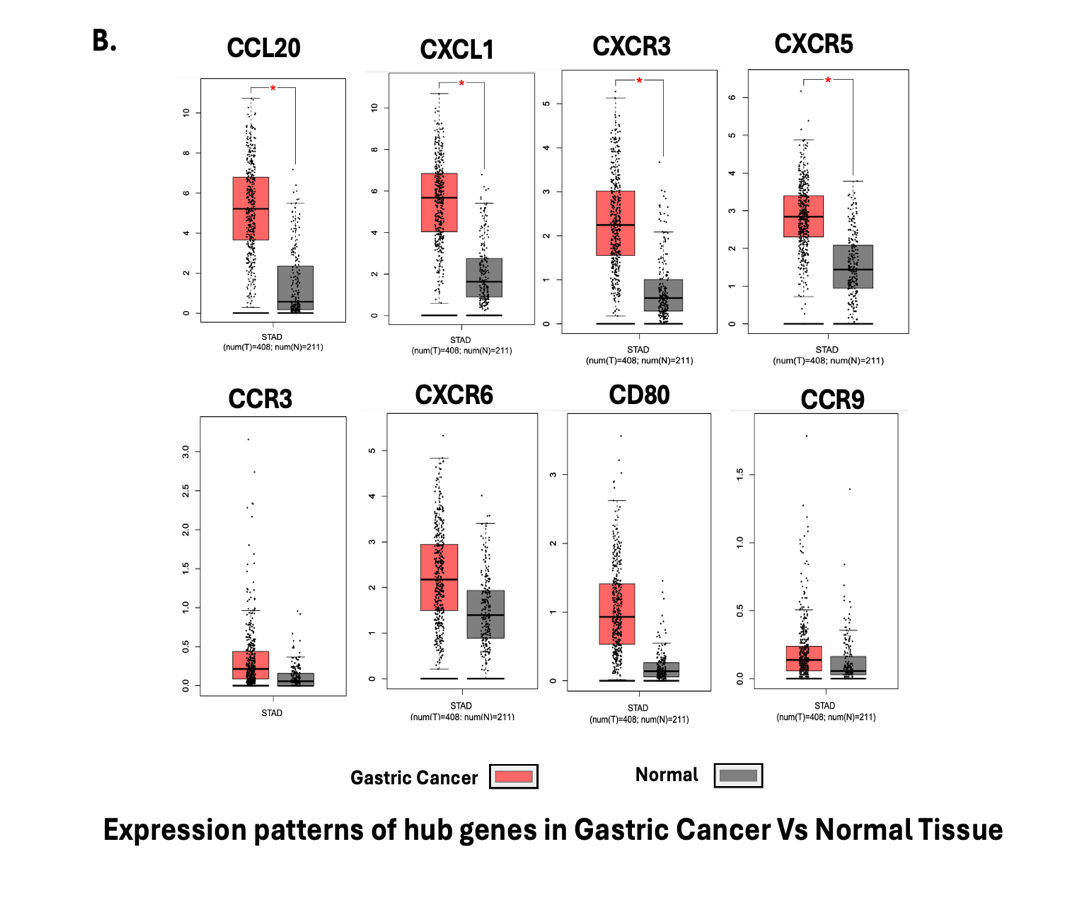

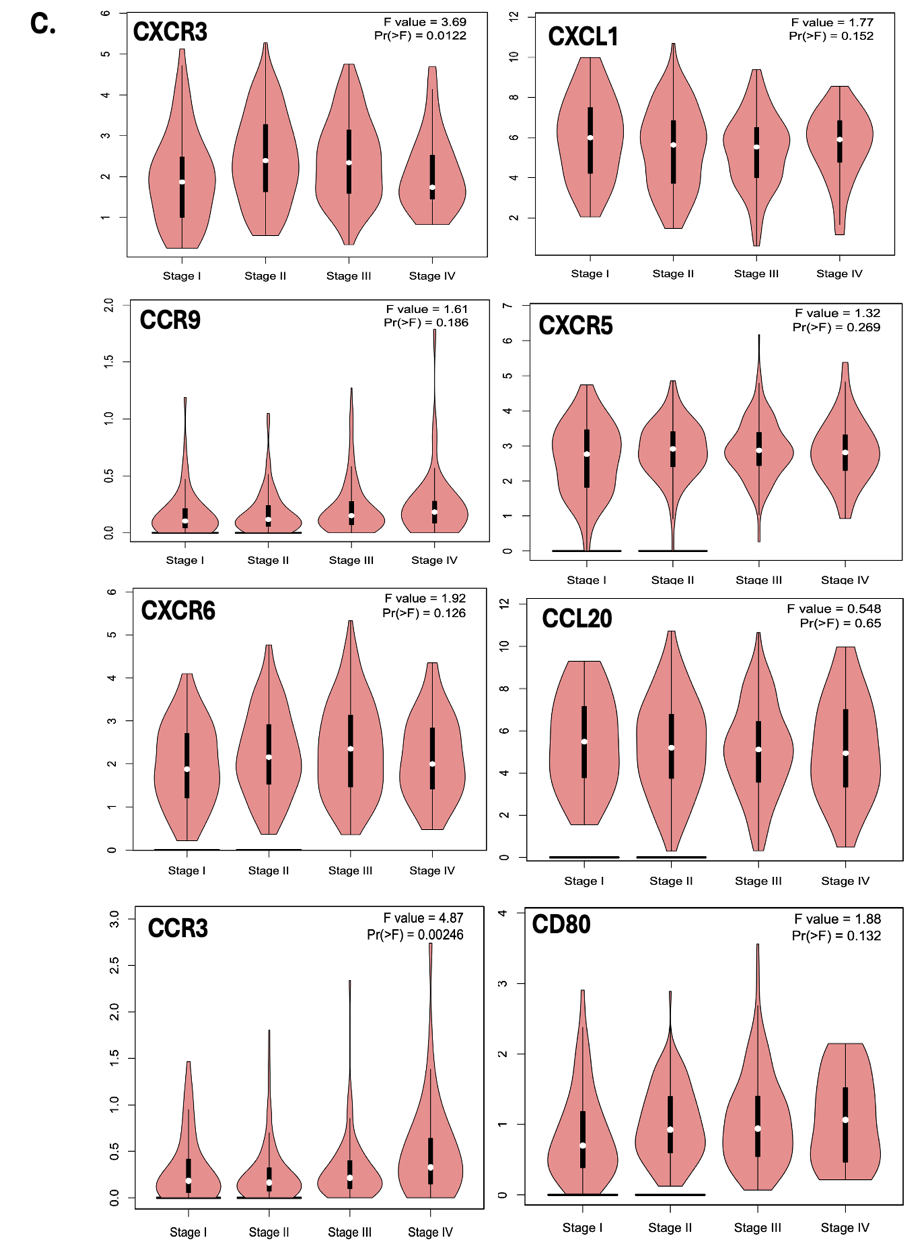


**Expression Patterns of Hub Genes Across Gastric Cancer Stages**

**Figure 2 Supplementary** demonstrates the expression patterns of hub genes across normal tissues, gastric cancer versus normal tissue, and different gastric cancer stages. **A.** Heatmap of transcript per million (TPM) values for hub genes **(*CXCL1, CCL20, CCR9, CCR3, CD80, CXCR5, CXCR3*, and *CXCR6*)** across normal tissues shows distinct tissue-specific expression, with notable clustering in immune and gastrointestinal tissues. **B.** Boxplots compare hub gene expression between gastric cancer and normal tissue, revealing significant upregulation of genes such as ***CCL20, CXCL1, CXCR3,* and *CXCR5*** in gastric cancer samples, highlighting their potential role in tumor progression. **C.** Violin plots illustrate the expression dynamics of hub genes across gastric cancer stages (I–IV), with genes such as ***CXCR3* and *CCR3*** showing stage-specific variations, reflecting their involvement in disease progression and malignancy. These findings provide insights into the biological significance of hub genes in gastric cancer development and progression.


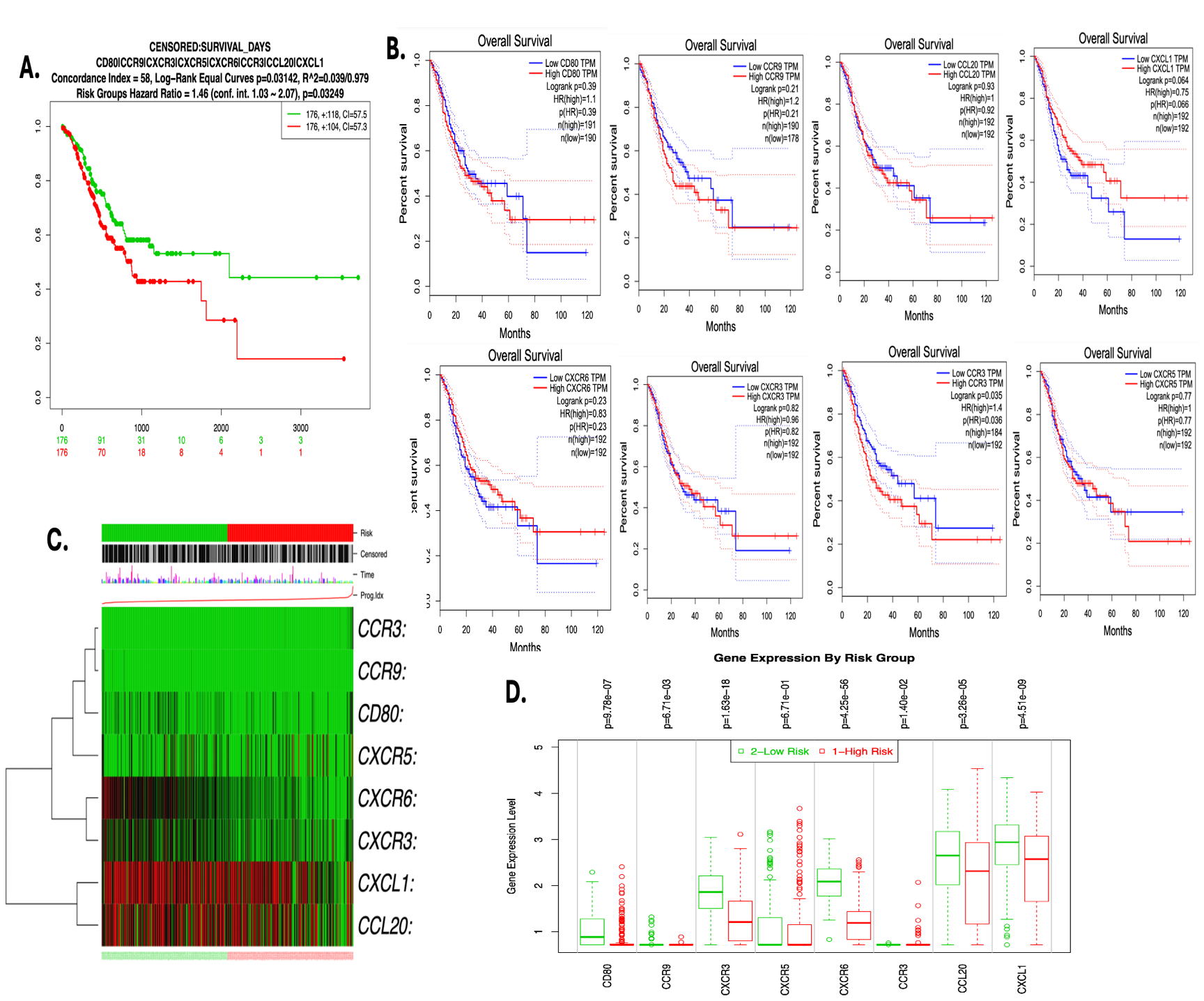


**Figure 3 Supplementary**. demonstrates survival analysis and biomarker validation for hub genes associated with ***H. pylori*-associated gastric cancer progression.** **A.** Kaplan-Meier survival curves stratify patients into high-risk and low-risk groups based on the expression of the combined hub genes **(*CCR3, CCR9, CD80, CXCR5, CXCR6, CXCR3, CXCL1, CCL20*),** showing significantly worse overall survival for high-risk patients (Log-Rank p = 0.032, Hazard Ratio = 1.46). **B.** Individual Kaplan-Meier survival plots for each hub gene reveal that high expression levels of ***CXCL1, CXCR5, CCR3*,** and ***CCL20*** are associated with poorer overall survival. **C.** Heatmap analysis highlights the expression of hub genes across high-risk and low-risk groups, with elevated expression in the high-risk cohort. **D.** Boxplots of gene expression levels by risk group demonstrate significantly higher expression of hub genes in high-risk patients. These results validate the prognostic significance of these hub genes and underscore their potential as biomarkers for predicting outcomes in ***H. pylori*-associated gastric cancer.**


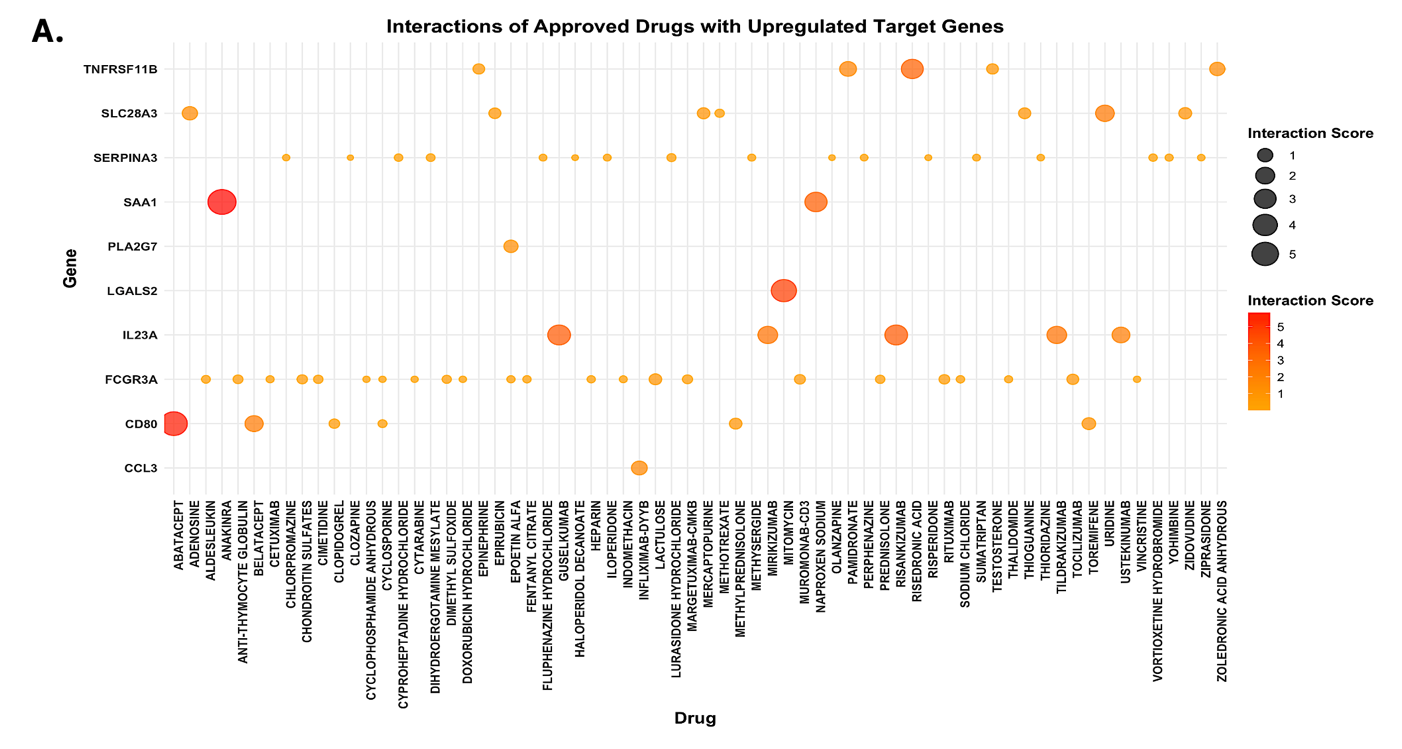

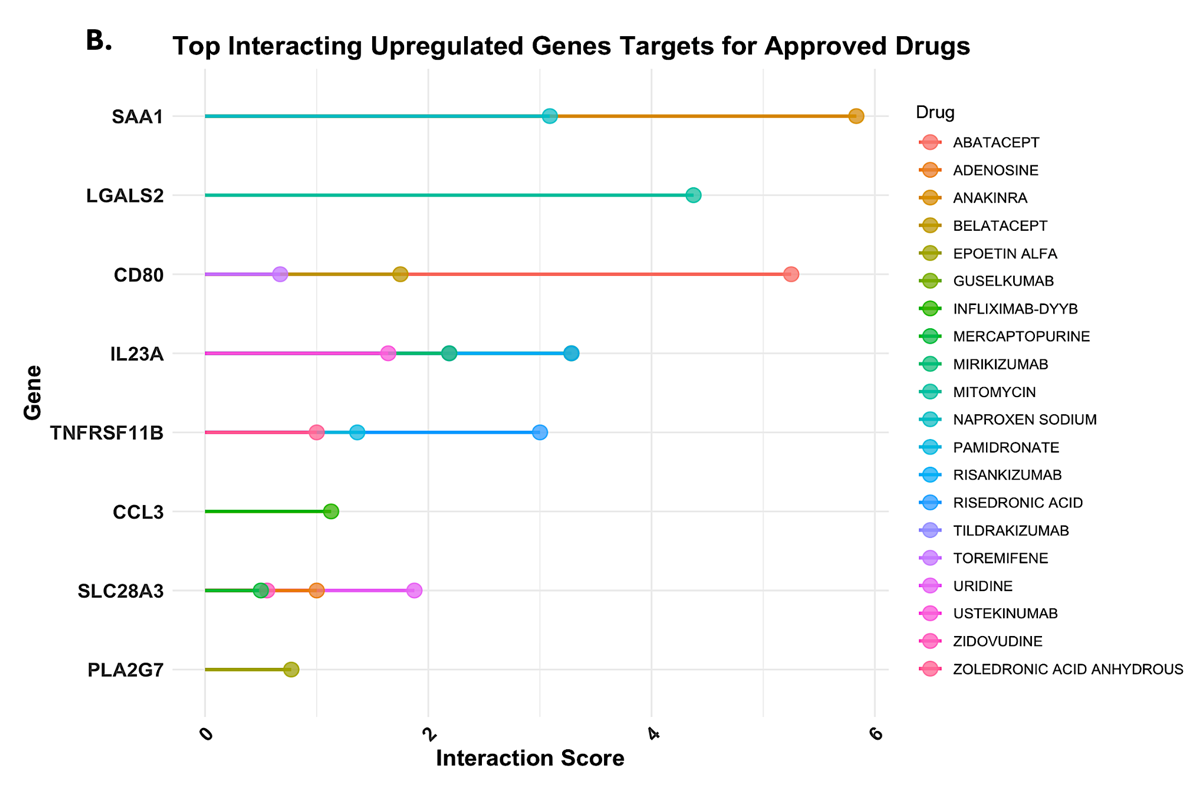


**Figure 4 Supplementary**. demonstrates the interaction of upregulated gene targets with approved drugs and drugs in developmental stages. **A.** Bubble plot shows interactions of approved drugs with upregulated gene targets, with ***CD80, CCL3, IL23A*, and *SAA1*** exhibiting high interaction scores, highlighting their relevance in approved therapeutic regimens. **B.** A lollipop plot of the top interacting genes for approved drugs reveals significant interactions for ***SAA1, CD80, CCL3*, and *TNFRSF11B***, associated with drugs like **Abatacept, Mercaptopurine, and Zoledronic Acid**, indicating their clinical relevance. **C.** Interactions of upregulated gene targets with drugs in development reveal potential therapeutic candidates, with genes such as ***CCL20, CXCL1, CD80*,** and ***IL23A*** showing high interaction scores. **D.** Detailed lollipop plot of the top interacting genes with developmental drugs highlights targets such as ***CCL20, CXCL1,* and *TNFRSF25***, linked to drugs like **Adjuvant, Antineoplastic Vaccine,** and **Relapladib**, offering promising avenues for novel therapies. These findings underscore the therapeutic potential of upregulated gene targets in ***H. pylori*-associated gastric cancer.**


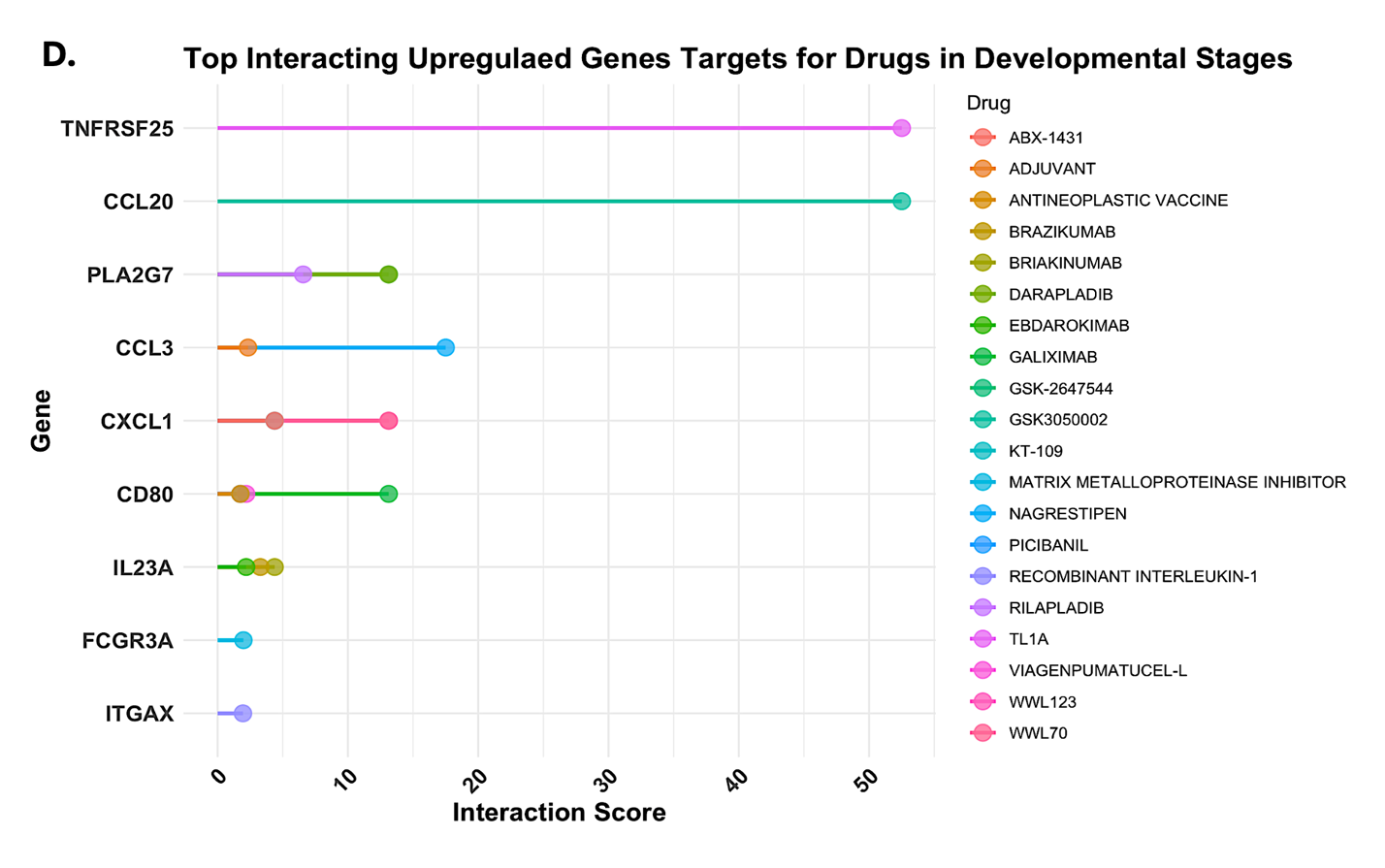

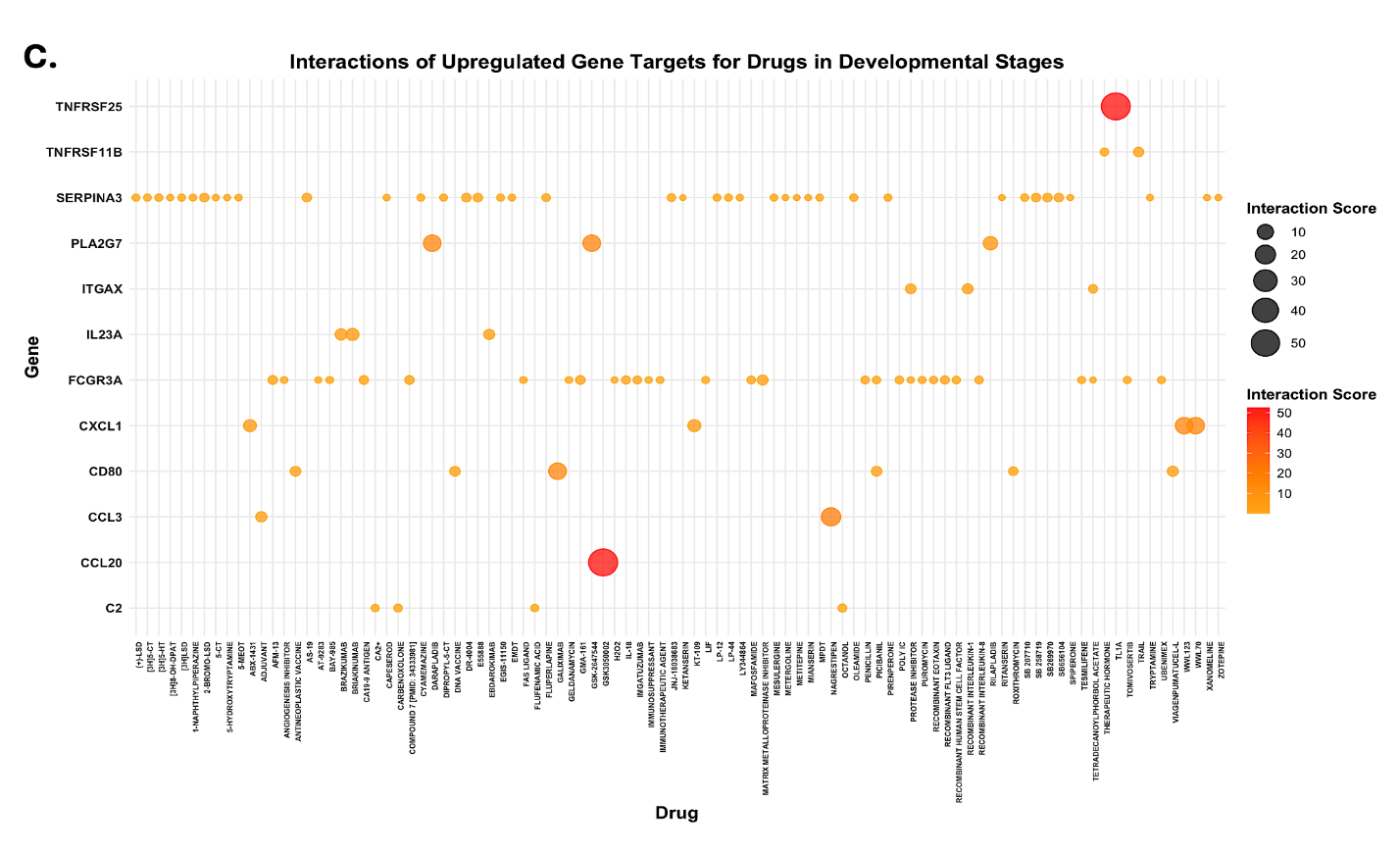

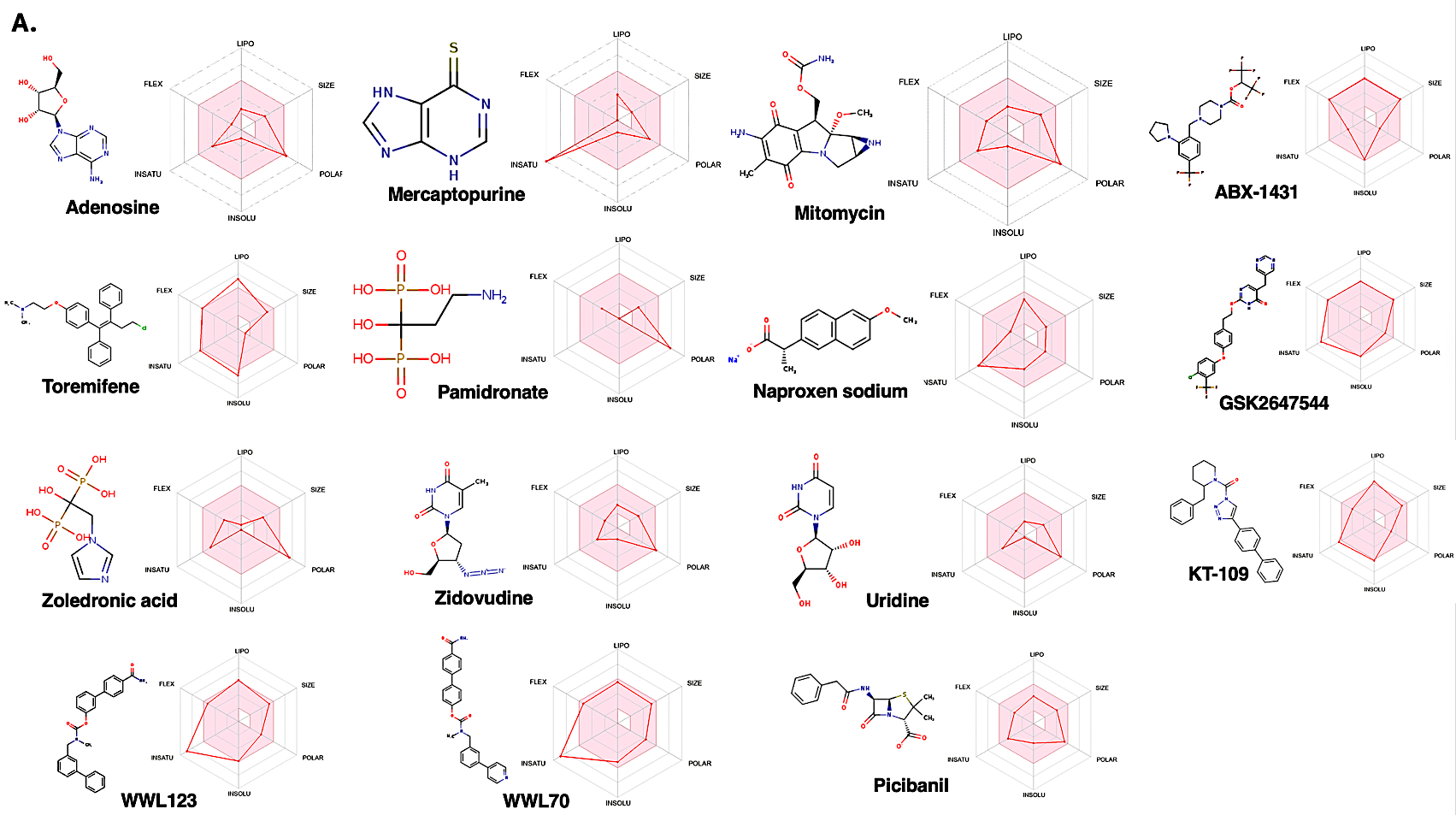

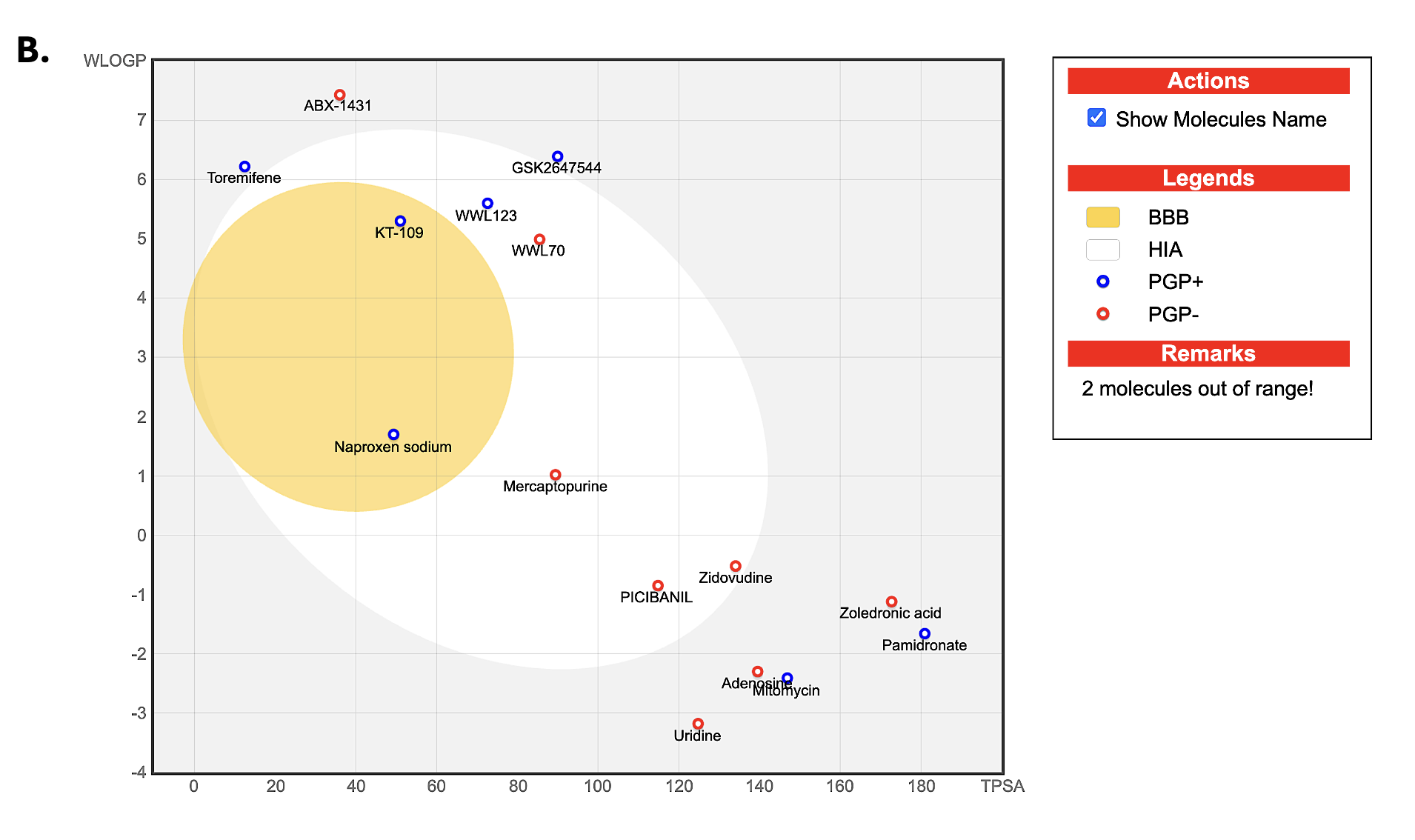


**Figure 5 Supplementary**. **In Silico Evaluation of Pharmacokinetics and Drug-Likeness of FDA-Approved and Developmental Drugs Targeting Upregulated Genes in H. pylori-Associated Gastric Cancer.** (A) Radar plots illustrating the physicochemical properties of a subset of FDA-approved drugs (Adenosine, Mercaptopurine, Mitomycin, Naproxen Sodium, Pamidronate, Toremifene, Zoledronic Acid, Uridine) and developmental drugs (ABX-1431, GSK-2879454, KT-109, Picibanil, WWL-123, WWL-70) identified through drug-gene interaction analysis. Each plot displays key parameters, including lipophilicity (LIPO), size (SIZE), polarity (POLAR), solubility (INSOLU), saturation (INSATU), and flexibility (FLEX), with red shaded areas indicating the optimal range for drug-likeness based on Lipinski’s Rule of Five and other pharmacokinetic criteria. (B) Scatter plot comparing the Topological Polar Surface Area (TPSA) and WLOGP (lipophilicity) values of the drugs, with the yellow shaded region representing the optimal range for good oral bioavailability (TPSA < 140 Å² and WLOGP between -0.4 and 5.6). Drugs are color-coded based on their predicted pharmacokinetic actions: yellow (BBB: blood-brain barrier permeability), white (HIA: human intestinal absorption), red (PGP+: P-glycoprotein substrate), and blue (PGP-: non-P-glycoprotein substrate). The analysis reveals that most drugs, such as Naproxen Sodium, Mitomycin, and GSK-2879454, exhibit favorable drug-likeness and bioavailability, supporting their potential as therapeutic candidates for targeting hub genes in H. pylori-associated gastric cancer, while Zoledronic Acid and Pamidronate show limitations in oral bioavailability.


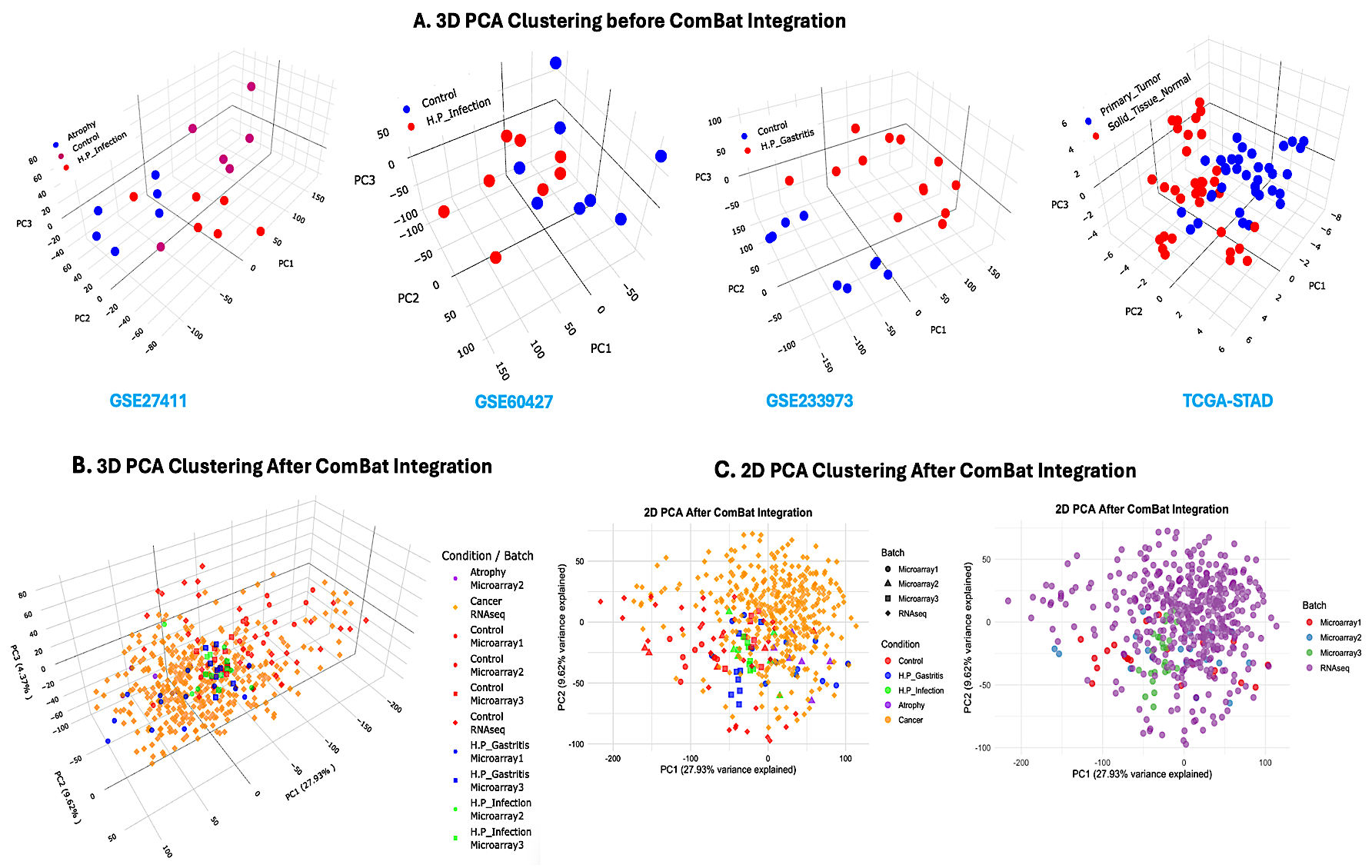


**Figure 6 Supplementary**. **PCA clustering visualization before and after ComBat integration.** (A) 3D-PCA clustering of individual datasets (GSE27411, GSE60427, GSE233973, and TCGA-STAD) before ComBat integration. Samples cluster primarily by platform and batch rather than by biological condition, indicating the presence of batch effects and platform-specific variability across microarray and RNA-seq datasets. (B) 3D PCA clustering after ComBat integration, demonstrating ComBat integration across datasets (GSE27411, GSE60427, GSE233973, TCGA-STAD) while preserving biological conditions (atrophy, H. pylori infection, gastritis, cancer). Colors represent different conditions, and shapes indicate batches (Microarray1, Microarray2, Microarray3, RNAseq). (C) 2D-PCA clustering after ComBat integration, further illustrating the removal of batch effects while maintaining biological variability. PC1 and PC2 explain 27.0% and 12.7% of the variance, respectively. Colors and shapes correspond to conditions and batches.
