# Supplementary material for: Multi-omics analysis of Helicobacter pylori–associated gastric cancer identifies hub genes as a novel therapeutic biomarker
Source: Brief Bioinform. 2025 May 30;26(3):bbaf241. doi: 10.1093/bib/bbaf241 (PMC12123523; doi:10.1093/bib/bbaf241)
Supplement: FinalHPyloriGraphs_bbaf241 [file finalhpylorigraphs_bbaf241.pdf]

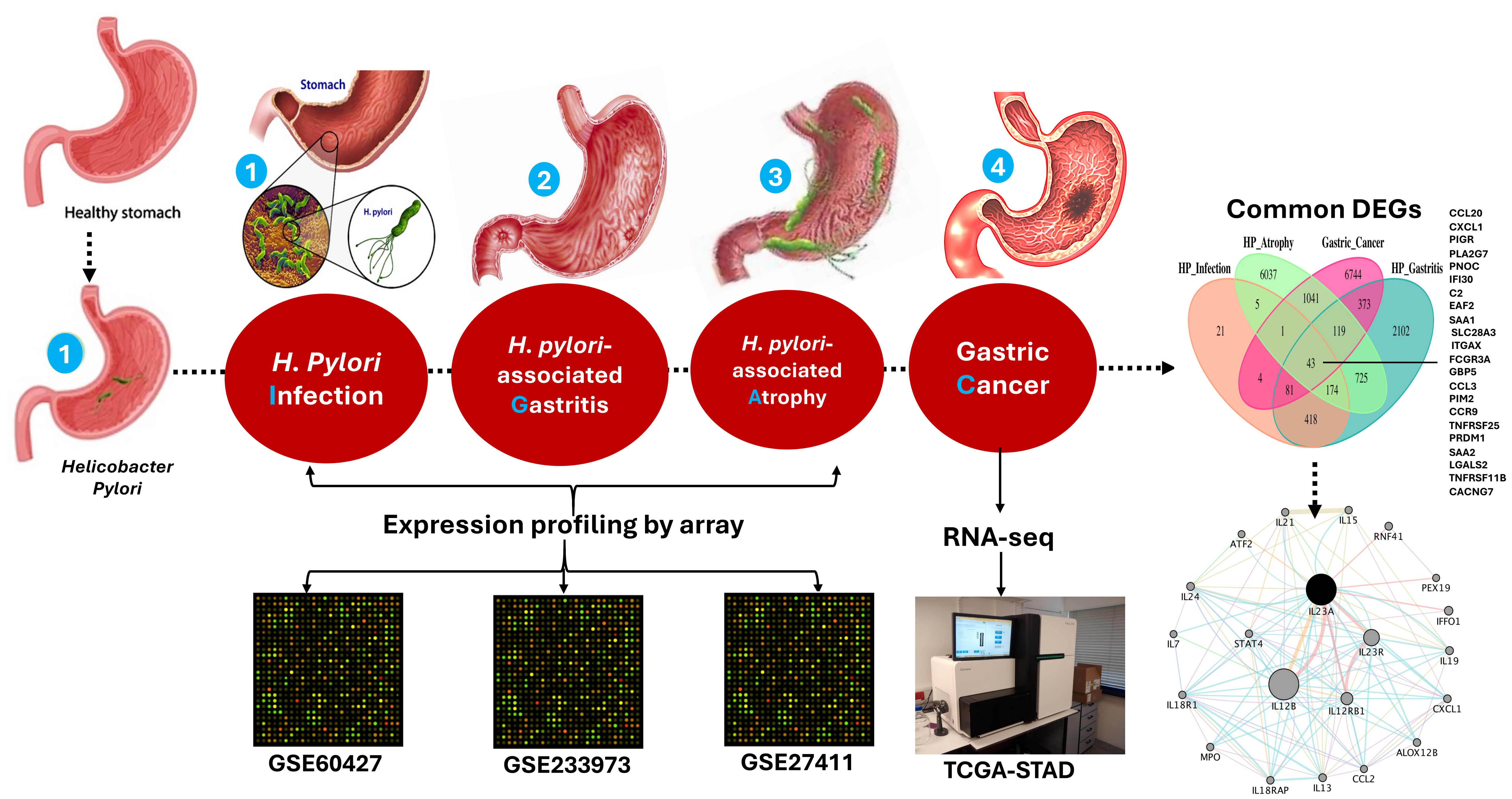

Healthy stomach
Helicobacter Pylori
Stomach
H. pylori
1
2
3
4
H. Pylori Infection
H. pylori-associated Gastritis
H. pylori-associated Atrophy
Gastric Cancer
Expression profiling by array
GSE60427
GSE233973
GSE27411
RNA-seq
TCGA-STAD
Common DEGs
HP_Infection
HP_Atrophy
Gastric_Cancer
HP_Gastritis
6037
6744
21
5
1041
373
2102
1
119
43
4
725
81
174
418
CCL20
CXCL1
PIGR
PLA2G7
PNOC
IFI30
C2
EAF2
SAA1
SLC28A3
ITGAX
FCGR3A
GBP5
CCL3
PIM2
CCR9
TNFRSF25
PRDM1
SAA2
LGALS2
TNFRSF11B
CACNG7
IL21
IL15
ATF2
RNF41
IL24
PEX19
IL23A
IFFO1
IL7
STAT4
IL23R
IL19
IL18R1
IL12B
IL12RB1
CXCL1
MPO
ALOX12B
IL18RAP
IL13
CCL2

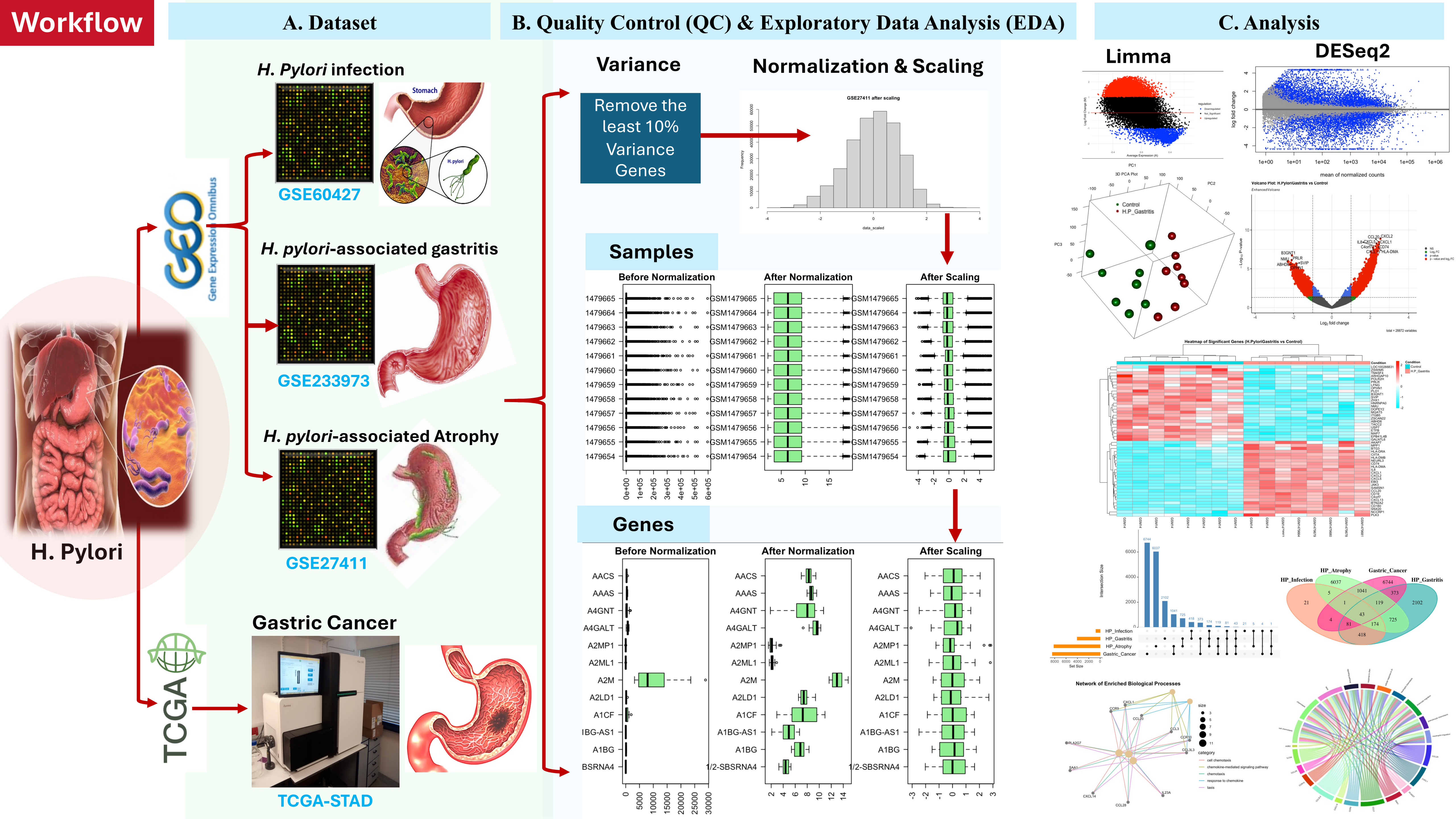

Workflow
A. Dataset
B. Quality Control (QC) & Exploratory Data Analysis (EDA)
C. Analysis
H. Pylori infection
GSE60427
H. pylori-associated gastritis
GSE233973
H. pylori-associated Atrophy
GSE27411
Gastric Cancer
TCGA-STAD
H. Pylori
Gene Expression Omnibus
TCGA
Variance
Remove the least 10% Variance Genes
Normalization & Scaling
GSE27411 after scaling
Samples
Before Normalization
After Normalization
After Scaling
Genes
Before Normalization
After Normalization
After Scaling
Limma
DESeq2
Heatmap of Significant Genes (H.PyloriGastritis vs Control)
Network of Enriched Biological Processes
HP_Infection
HP_Atrophy
Gastric_Cancer
HP_Gastritis

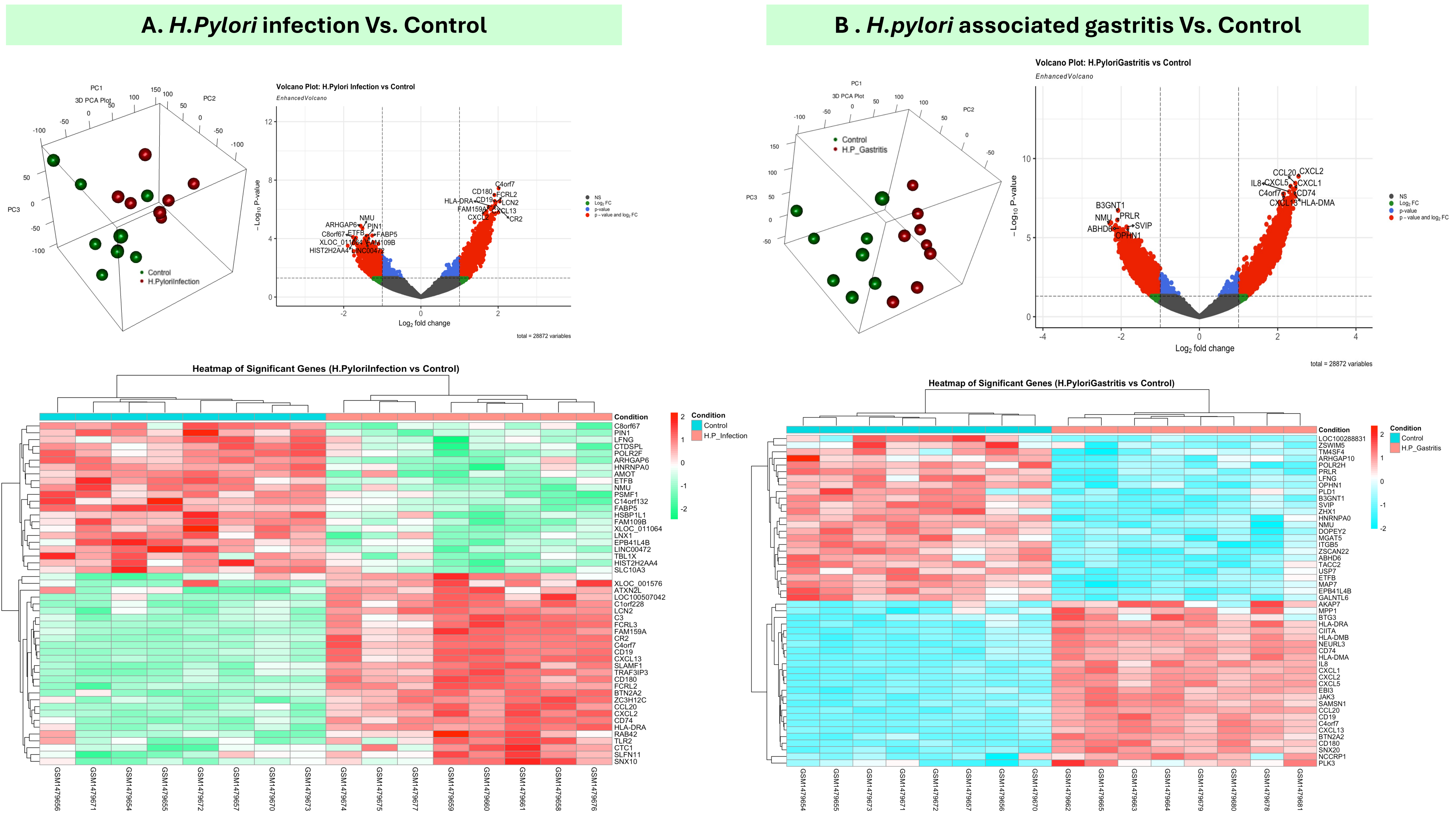
A. H.Pylori infection Vs. Control
B . H.pylori associated gastritis Vs. Control
3D PCA Plot
PC1
PC2
PC3
Control
H.PyloriInfection
H.P_Gastritis
Volcano Plot: H.Pylori Infection vs Control
EnhancedVolcano
Volcano Plot: H.PyloriGastritis vs Control
NS
Log2 FC
p-value
p – value and log2 FC
Log2 fold change
– Log10 P-value
total = 28872 variables
C4orf7
CD180
FCRL2
HLA-DRA
CD19
LCN2
FAM159A
CXCL13
CXCL2
CR2
NMU
ARHGAP6
PIN1
C8orf67
ETFB
FABP5
XLOC_011064
FAM109B
HIST2H2AA4
LINC00472
CCL20
CXCL2
IL8
CXCL5
CXCL1
C4orf7
CD74
CXCL13
HLA-DMA
B3GNT1
NMU
PRLR
SVIP
ABHD6
OPHN1
Heatmap of Significant Genes (H.PyloriInfection vs Control)
Condition
Control
H.P_Infection
C8orf67
PIN1
LFNG
CTDSPL
POLR2F
ARHGAP6
HNRNPA0
AMOT
ETFB
NMU
PSMF1
C14orf132
FABP5
HSBP1L1
FAM109B
XLOC_011064
LNX1
EPB41L4B
LINC00472
TBL1X
HIST2H2AA4
SLC10A3
XLOC_001576
ATXN2L
LOC100507042
C1orf228
LCN2
C3
FCRL3
FAM159A
CR2
C4orf7
CD19
CXCL13
SLAMF1
TRAF3IP3
CD180
FCRL2
BTN2A2
ZC3H12C
CCL20
CXCL2
CD74
HLA-DRA
RAB42
TLR2
CTC1
SLFN11
SNX10
GSM1479656
GSM1479671
GSM1479654
GSM1479655
GSM1479672
GSM1479657
GSM1479670
GSM1479673
GSM1479674
GSM1479675
GSM1479677
GSM1479659
GSM1479660
GSM1479661
GSM1479658
GSM1479676
Heatmap of Significant Genes (H.PyloriGastritis vs Control)
Condition
Control
H.P_Gastritis
LOC100288831
ZSWIM5
TM4SF4
ARHGAP10
POLR2H
PRLR
LFNG
OPHN1
PLD1
B3GNT1
SVIP
ZHX1
HNRNPA0
NMU
DOPEY2
MGAT5
ITGB5
ZSCAN22
ABHD6
TACC2
USP7
ETFB
MAP7
EPB41L4B
GALNTL6
AKAP7
MPP1
BTG3
HLA-DRA
CIITA
HLA-DMB
NEURL3
CD74
HLA-DMA
IL8
CXCL1
CXCL2
CXCL5
EBI3
JAK3
SAMSN1
CCL20
CD19
C4orf7
CXCL13
BTN2A2
CD180
SNX20
NCCRP1
PLK3
GSM1479654
GSM1479655
GSM1479673
GSM1479671
GSM1479672
GSM1479657
GSM1479656
GSM1479670
GSM1479662
GSM1479665
GSM1479663
GSM1479664
GSM1479679
GSM1479680
GSM1479678
GSM1479681

C. *H. pylori*-associated atrophy Vs. Control

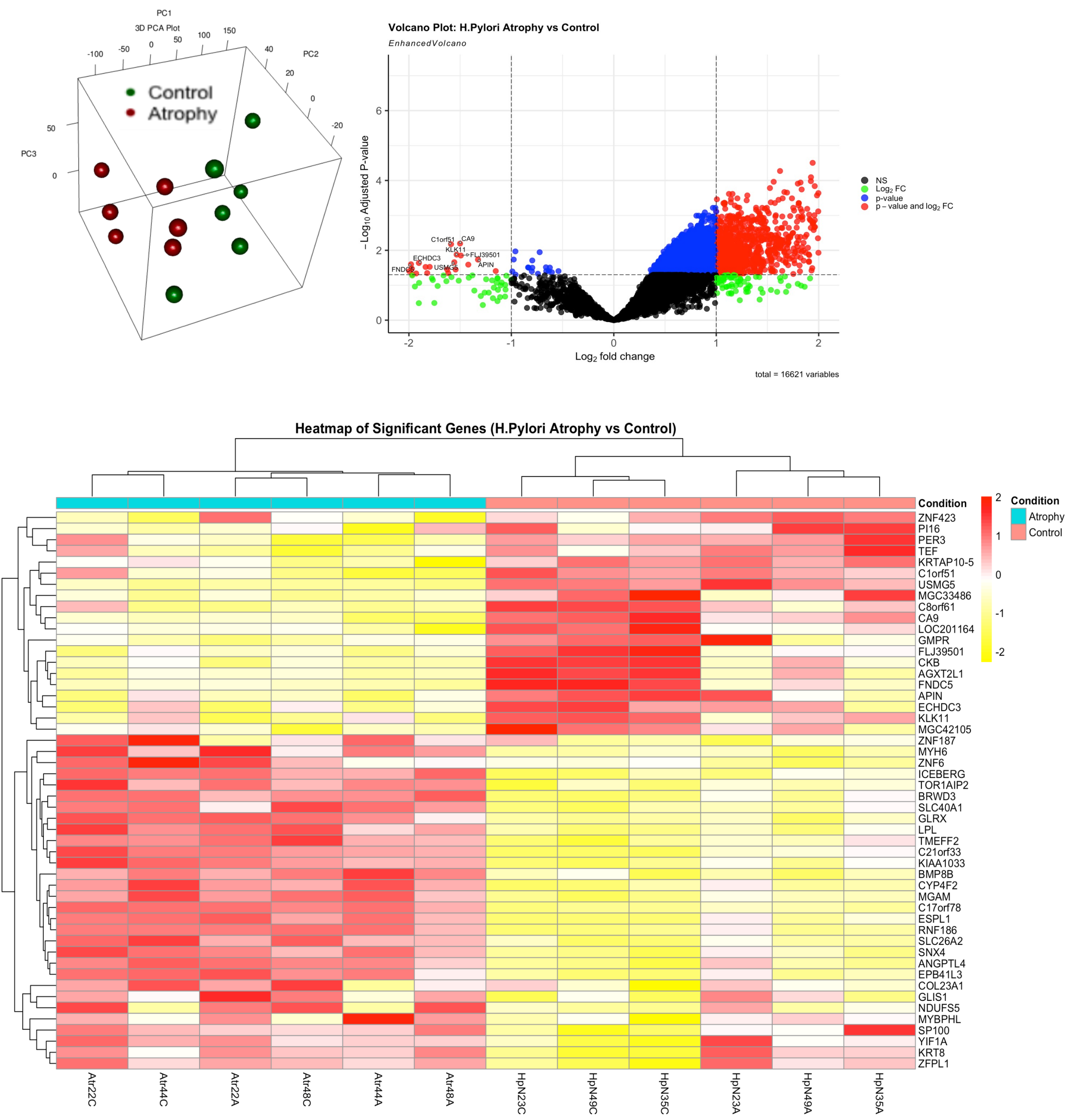

D. Gastric Cancer Vs. Control

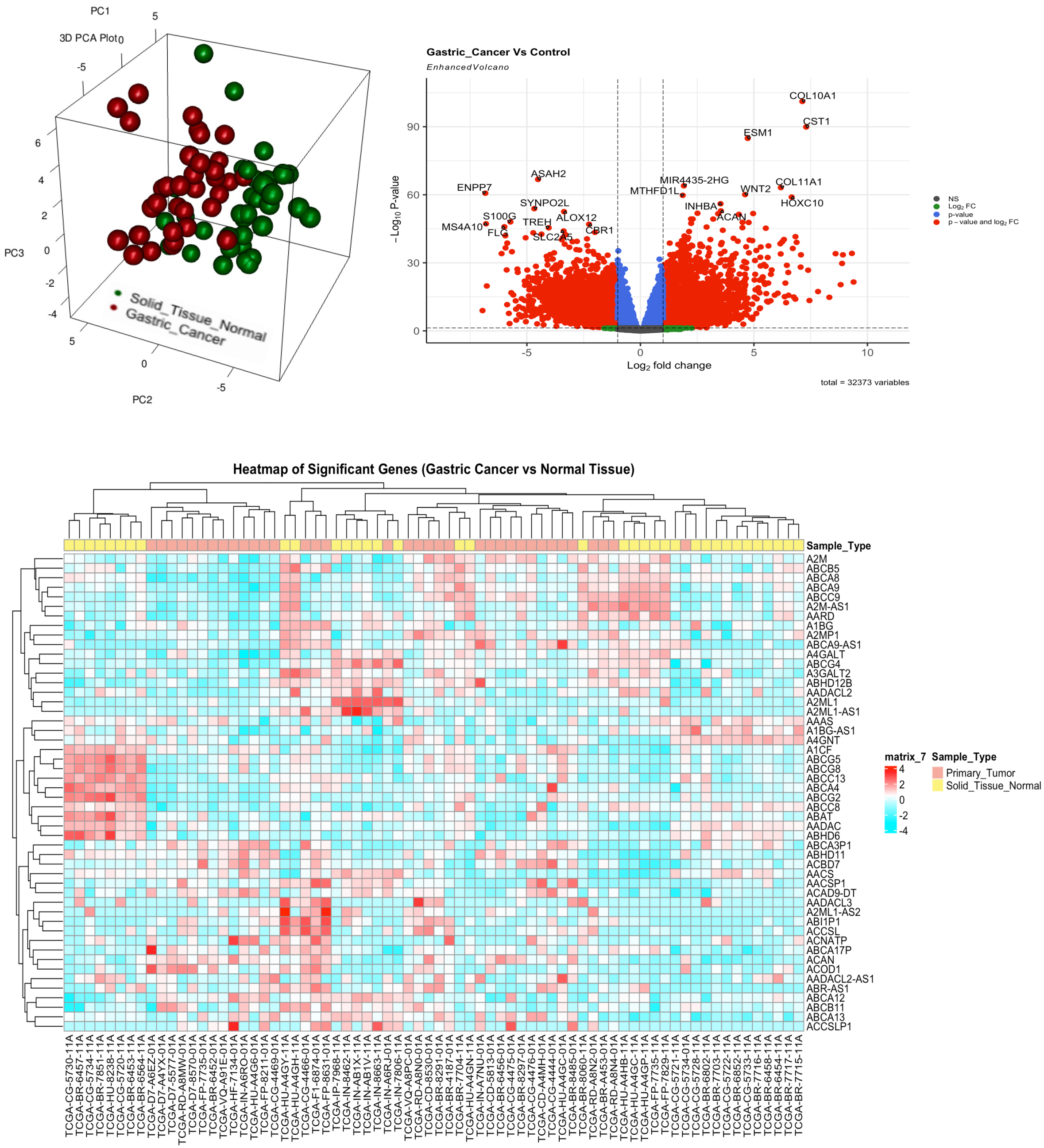

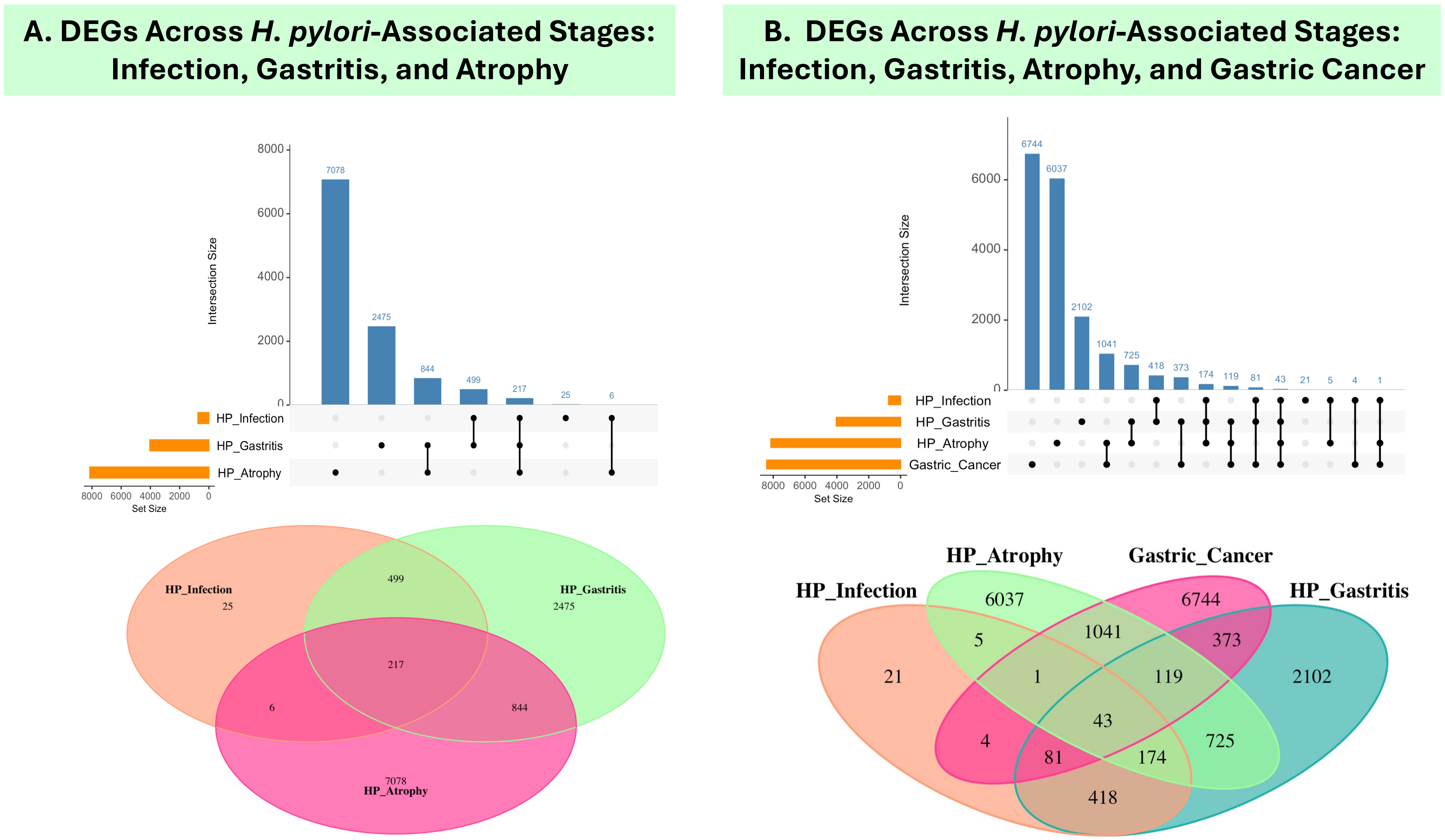
A. DEGs Across H. pylori-Associated Stages: Infection, Gastritis, and Atrophy
B. DEGs Across H. pylori-Associated Stages: Infection, Gastritis, Atrophy, and Gastric Cancer
Intersection Size
Set Size
HP_Infection
HP_Gastritis
HP_Atrophy
Gastric_Cancer
7078
2475
844
499
217
25
6
6744
6037
2102
1041
725
418
373
174
119
81
43
21
5
4
1

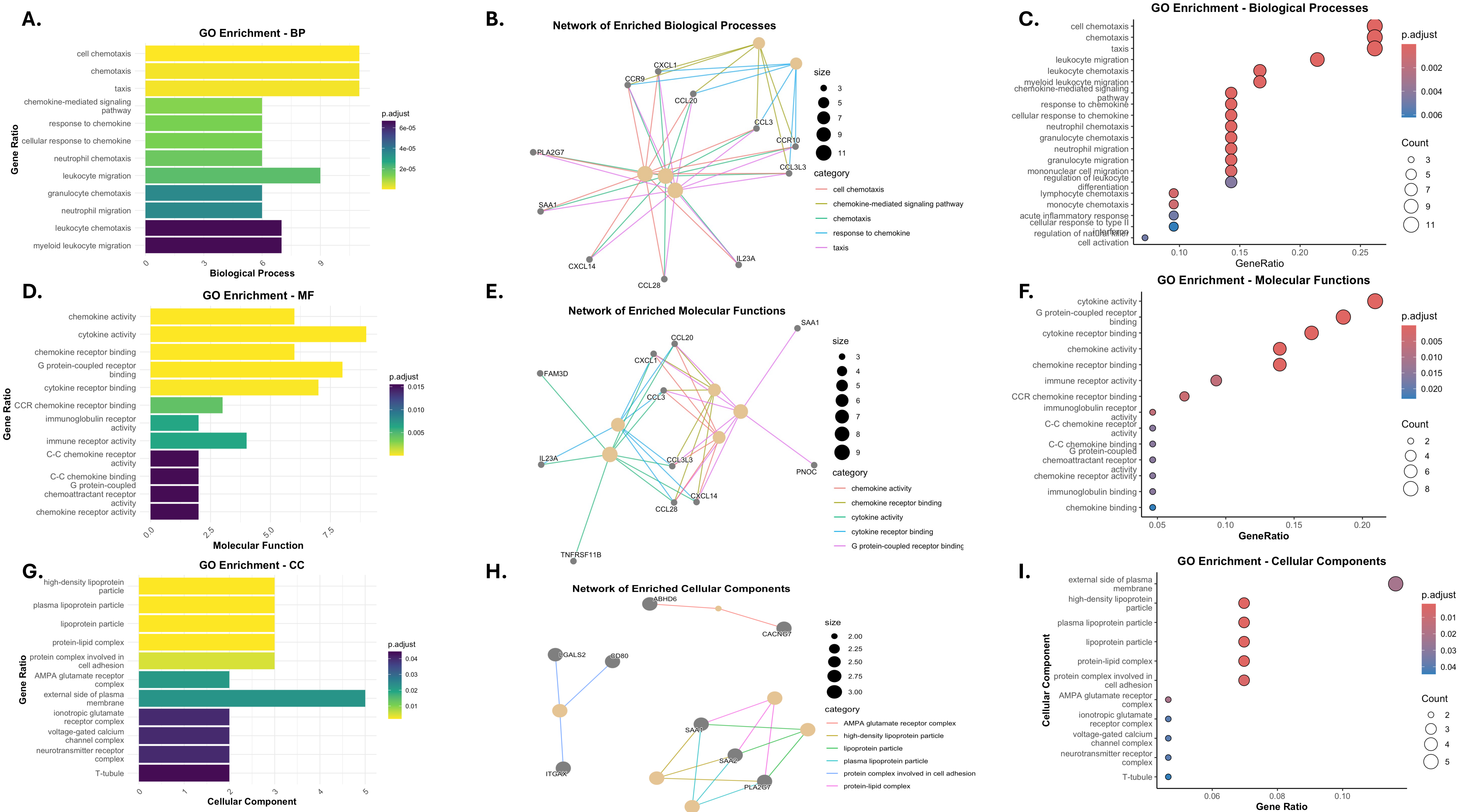
A.
GO Enrichment - BP
cell chemotaxis
chemotaxis
taxis
chemokine-mediated signaling pathway
response to chemokine
cellular response to chemokine
neutrophil chemotaxis
leukocyte migration
granulocyte chemotaxis
neutrophil migration
leukocyte chemotaxis
myeloid leukocyte migration
Gene Ratio
Biological Process
p.adjust
6e-05
4e-05
2e-05
B.
Network of Enriched Biological Processes
CXCL1
CCR9
CCL20
CCL3
CCR10
CCL3L3
PLA2G7
SAA1
CXCL14
CCL28
IL23A
size
3
5
7
9
11
category
cell chemotaxis
chemokine-mediated signaling pathway
chemotaxis
response to chemokine
taxis
C.
GO Enrichment - Biological Processes
cell chemotaxis
chemotaxis
taxis
leukocyte migration
leukocyte chemotaxis
myeloid leukocyte migration
chemokine-mediated signaling pathway
response to chemokine
cellular response to chemokine
neutrophil chemotaxis
granulocyte chemotaxis
neutrophil migration
granulocyte migration
mononuclear cell migration
regulation of leukocyte differentiation
lymphocyte chemotaxis
monocyte chemotaxis
acute inflammatory response
cellular response to type II interferon
regulation of natural killer cell activation
GeneRatio
p.adjust
0.002
0.004
0.006
Count
D.
GO Enrichment - MF
chemokine activity
cytokine activity
chemokine receptor binding
G protein-coupled receptor binding
cytokine receptor binding
CCR chemokine receptor binding
immunoglobulin receptor activity
immune receptor activity
C-C chemokine receptor activity
C-C chemokine binding
G protein-coupled chemoattractant receptor activity
chemokine receptor activity
Gene Ratio
Molecular Function
p.adjust
0.015
0.010
0.005
E.
Network of Enriched Molecular Functions
SAA1
CCL20
CXCL1
FAM3D
CCL3
IL23A
CCL3L3
PNOC
CXCL14
CCL28
TNFRSF11B
size
category
chemokine activity
chemokine receptor binding
cytokine activity
cytokine receptor binding
G protein-coupled receptor binding
F.
GO Enrichment - Molecular Functions
cytokine activity
G protein-coupled receptor binding
cytokine receptor binding
chemokine activity
chemokine receptor binding
immune receptor activity
CCR chemokine receptor binding
immunoglobulin receptor activity
C-C chemokine receptor activity
C-C chemokine binding
G protein-coupled chemoattractant receptor activity
chemokine receptor activity
immunoglobulin binding
chemokine binding
GeneRatio
p.adjust
0.005
0.010
0.015
0.020
Count
G.
GO Enrichment - CC
high-density lipoprotein particle
plasma lipoprotein particle
lipoprotein particle
protein-lipid complex
protein complex involved in cell adhesion
AMPA glutamate receptor complex
external side of plasma membrane
ionotropic glutamate receptor complex
voltage-gated calcium channel complex
neurotransmitter receptor complex
T-tubule
Gene Ratio
Cellular Component
p.adjust
0.04
0.03
0.02
0.01
H.
Network of Enriched Cellular Components
ABHD6
CACNG7
LGALS2
CD80
ITGAX
SAA1
SAA2
PLA2G7
size
2.00
2.25
2.50
2.75
3.00
category
AMPA glutamate receptor complex
high-density lipoprotein particle
lipoprotein particle
plasma lipoprotein particle
protein complex involved in cell adhesion
protein-lipid complex
I.
GO Enrichment - Cellular Components
external side of plasma membrane
high-density lipoprotein particle
plasma lipoprotein particle
lipoprotein particle
protein-lipid complex
protein complex involved in cell adhesion
AMPA glutamate receptor complex
ionotropic glutamate receptor complex
voltage-gated calcium channel complex
neurotransmitter receptor complex
T-tubule
Cellular Component
Gene Ratio
p.adjust
0.01
0.02
0.03
0.04
Count

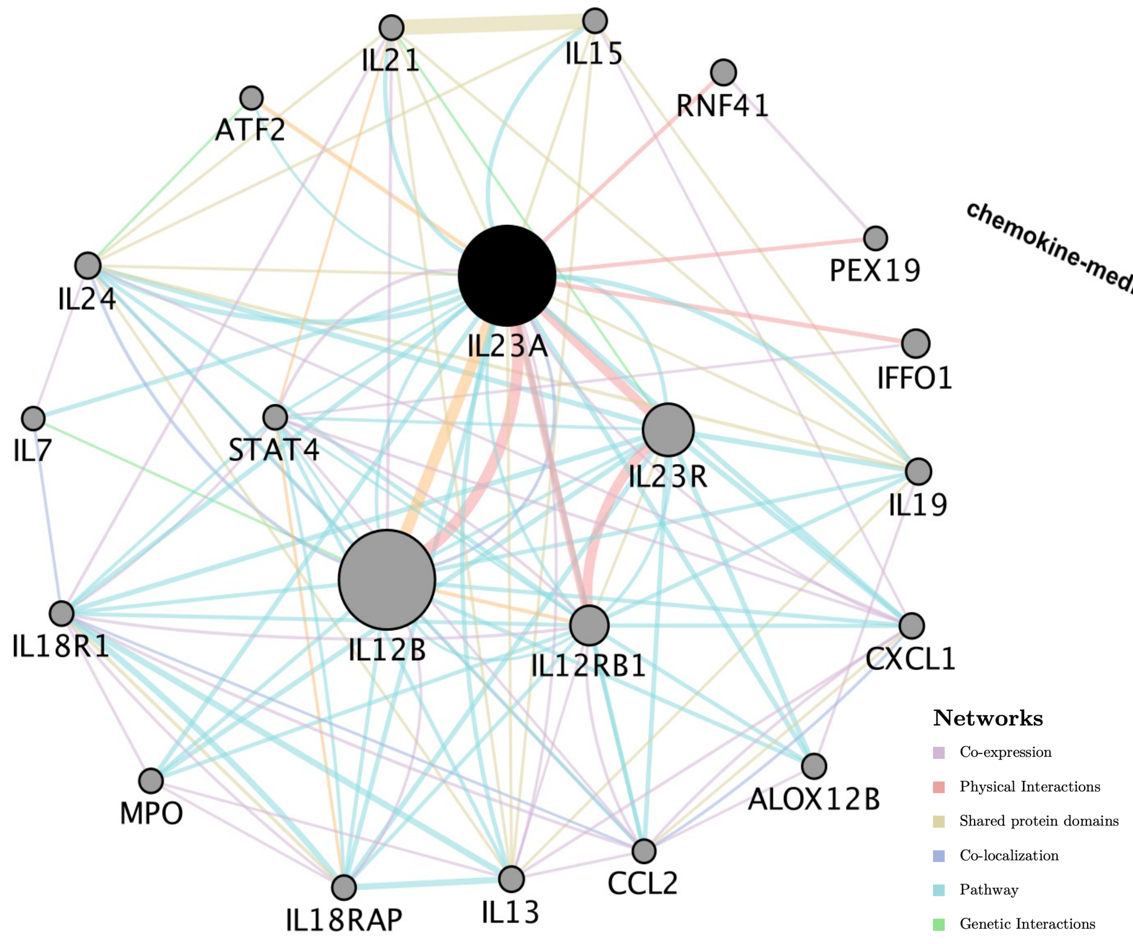

A. Network analysis for All DEGs across *H.Pylori* associated gastric cancer progression

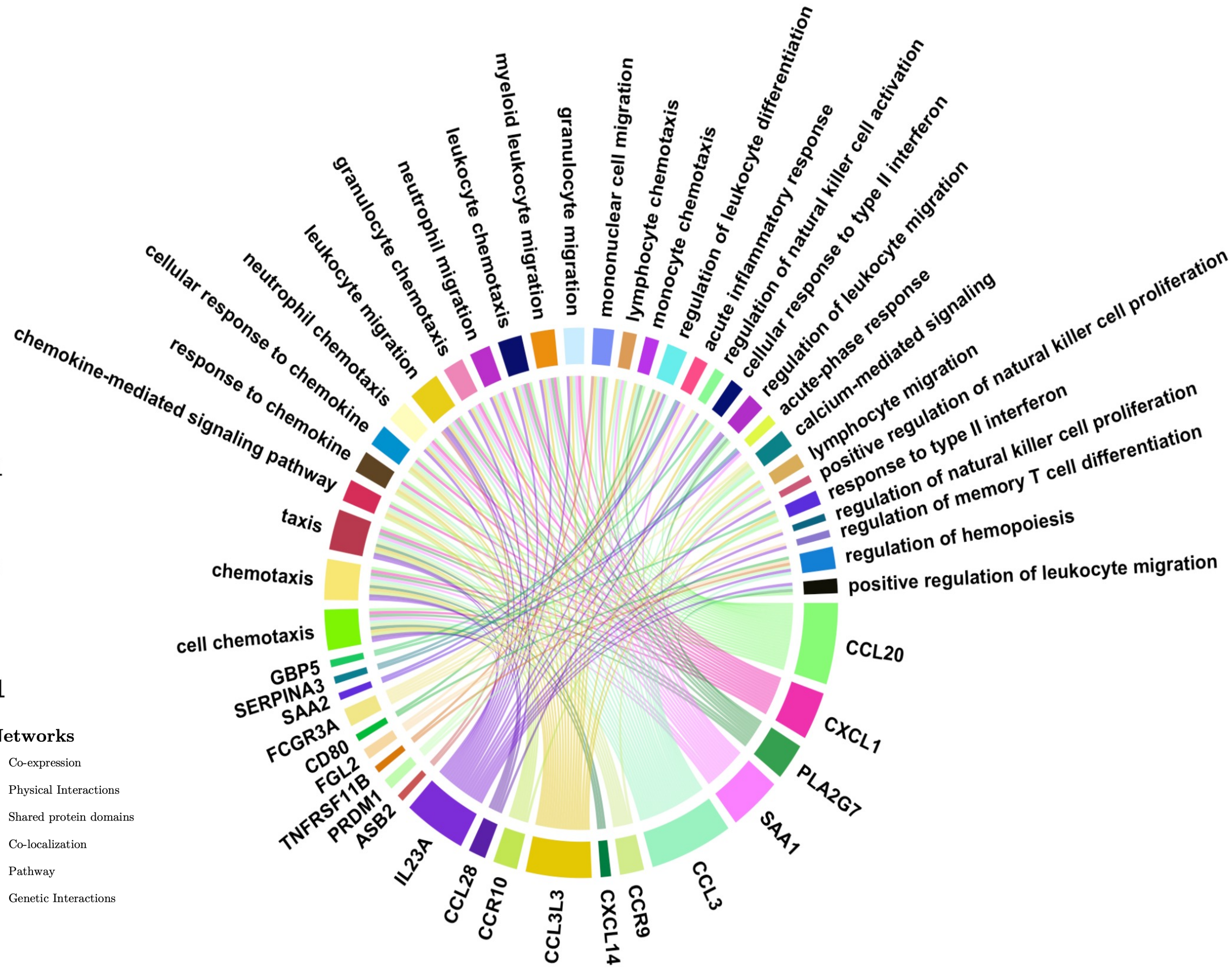

B. Chord Diagram of GO Terms and Gene Associations

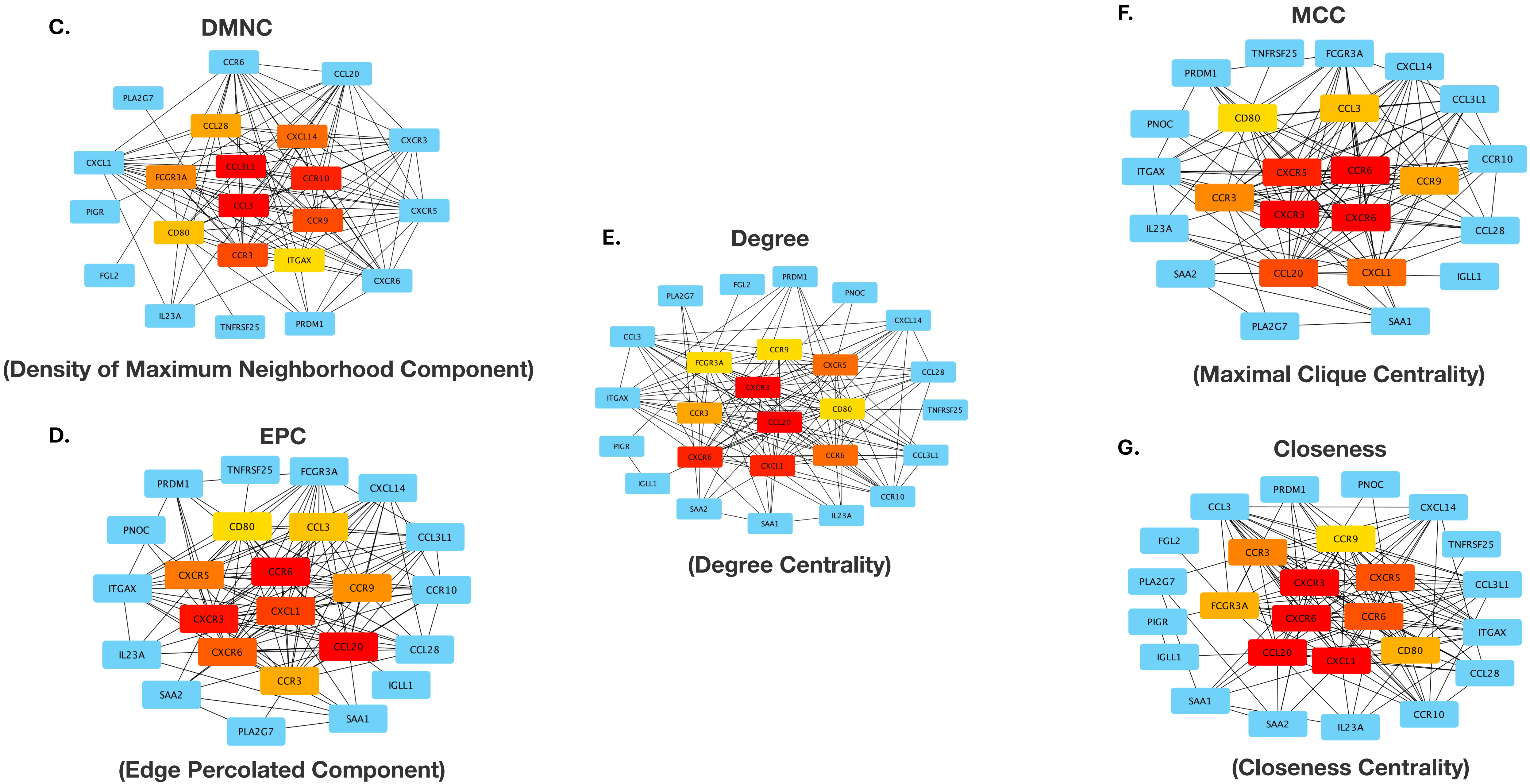
C.
DMNC
CCR6
CCL20
PLA2G7
CCL28
CXCL14
CXCR3
CXCL1
CCL3L1
FCGR3A
CCR10
PIGR
CCL3
CXCR5
CCR9
CD80
CCR3
ITGAX
FGL2
CXCR6
IL23A
TNFRSF25
PRDM1
(Density of Maximum Neighborhood Component)
D.
EPC
PRDM1
TNFRSF25
FCGR3A
CXCL14
PNOC
CD80
CCL3
CCL3L1
CXCR5
CCR6
CCR9
ITGAX
CCR10
CXCL1
CXCR3
IL23A
CXCR6
CCL20
CCL28
CCR3
IGLL1
SAA2
SAA1
PLA2G7
(Edge Percolated Component)
E.
Degree
PRDM1
FGL2
PLA2G7
PNOC
CXCL14
CCL3
CCR9
FCGR3A
CXCR5
CCL28
CXCR3
ITGAX
CD80
TNFRSF25
CCR3
CCL20
PIGR
CXCR6
CCR6
CCL3L1
CXCL1
IGLL1
CCR10
SAA2
IL23A
SAA1
(Degree Centrality)
F.
MCC
TNFRSF25
FCGR3A
PRDM1
CXCL14
CCL3L1
CCL3
CD80
PNOC
CCR10
ITGAX
CXCR5
CCR6
CCR9
CCR3
IL23A
CXCR3
CXCR6
CCL28
SAA2
CCL20
CXCL1
IGLL1
SAA1
PLA2G7
(Maximal Clique Centrality)
G.
Closeness
PRDM1
PNOC
CCL3
CXCL14
FGL2
CCR9
TNFRSF25
CCR3
CXCR3
CXCR5
PLA2G7
CCL3L1
FCGR3A
PIGR
CXCR6
CCR6
ITGAX
IGLL1
CCL20
CD80
CXCL1
CCL28
SAA1
SAA2
IL23A
CCR10
(Closeness Centrality)

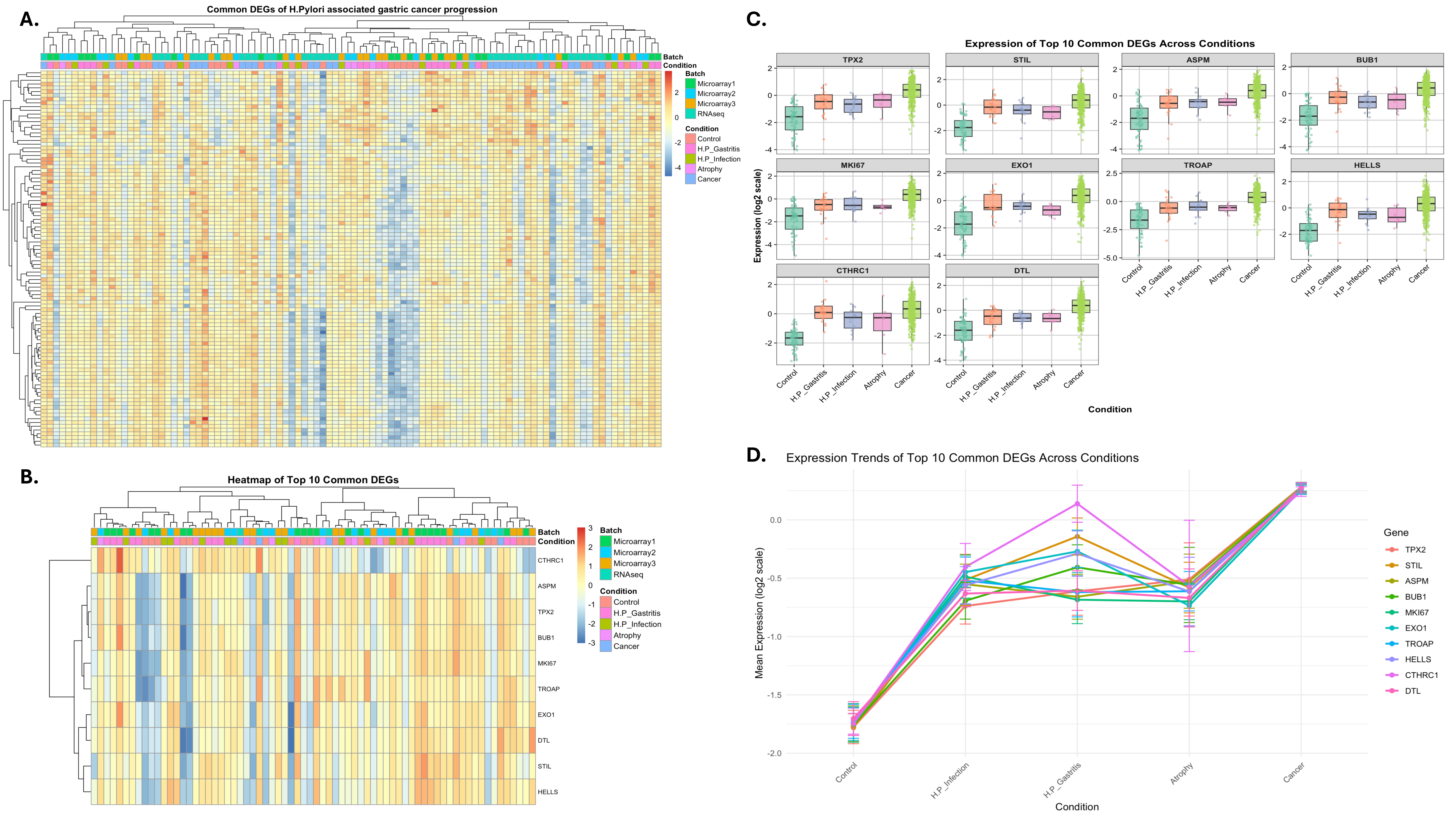
A.
Common DEGs of H.Pylori associated gastric cancer progression
Batch
Condition
Batch
Microarray1
Microarray2
Microarray3
RNAseq
Condition
Control
H.P_Gastritis
H.P_Infection
Atrophy
Cancer
B.
Heatmap of Top 10 Common DEGs
Batch
Condition
CTHRC1
ASPM
TPX2
BUB1
MKI67
TROAP
EXO1
DTL
STIL
HELLS
C.
Expression of Top 10 Common DEGs Across Conditions
TPX2
STIL
ASPM
BUB1
MKI67
EXO1
TROAP
HELLS
CTHRC1
DTL
Expression (log2 scale)
Control
H.P_Gastritis
H.P_Infection
Atrophy
Cancer
Condition
D.
Expression Trends of Top 10 Common DEGs Across Conditions
Mean Expression (log2 scale)
Control
H.P_Infection
H.P_Gastritis
Atrophy
Cancer
Condition
Gene
TPX2
STIL
ASPM
BUB1
MKI67
EXO1
TROAP
HELLS
CTHRC1
DTL

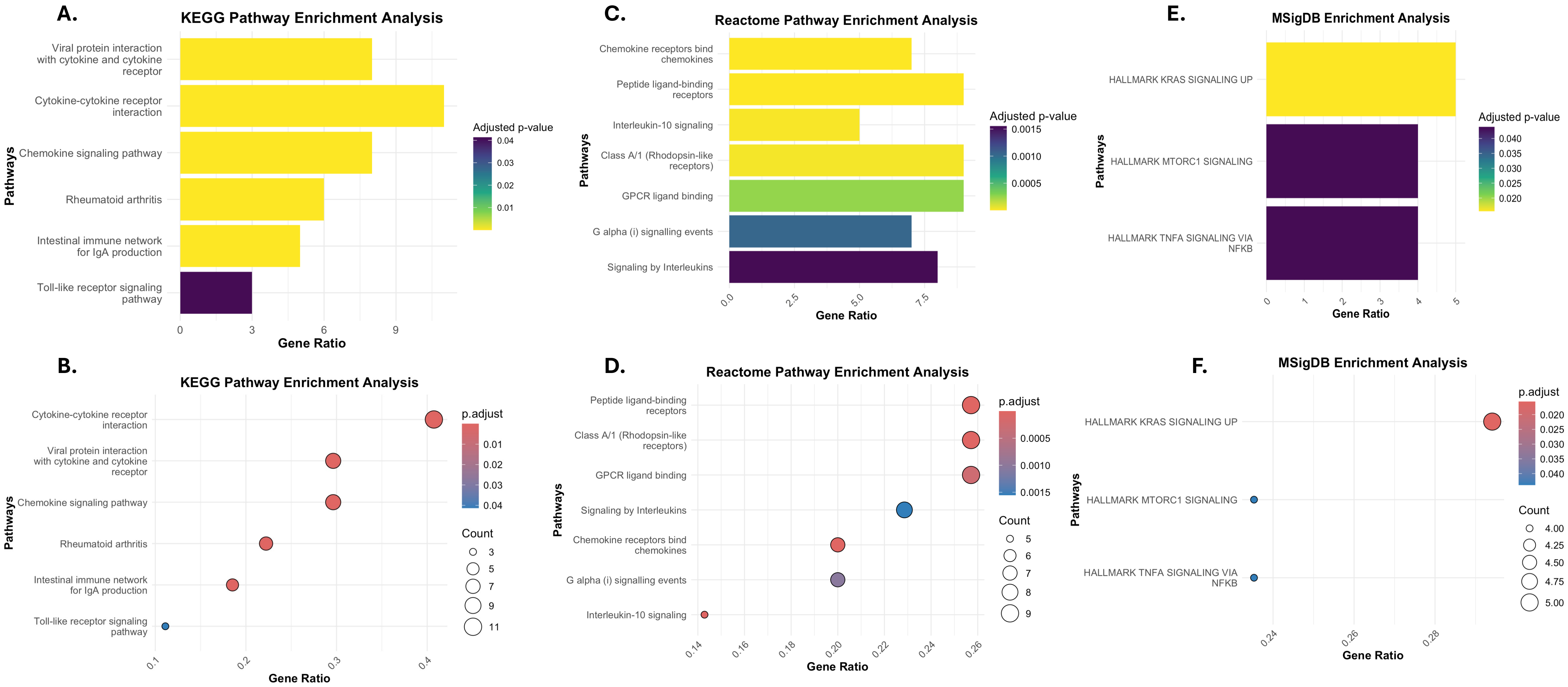
A.
KEGG Pathway Enrichment Analysis
Viral protein interaction with cytokine and cytokine receptor
Cytokine-cytokine receptor interaction
Chemokine signaling pathway
Rheumatoid arthritis
Intestinal immune network for IgA production
Toll-like receptor signaling pathway
Pathways
Gene Ratio
Adjusted p-value
C.
Reactome Pathway Enrichment Analysis
Chemokine receptors bind chemokines
Peptide ligand-binding receptors
Interleukin-10 signaling
Class A/1 (Rhodopsin-like receptors)
GPCR ligand binding
G alpha (i) signalling events
Signaling by Interleukins
Pathways
Gene Ratio
Adjusted p-value
E.
MSigDB Enrichment Analysis
HALLMARK KRAS SIGNALING UP
HALLMARK MTORC1 SIGNALING
HALLMARK TNFA SIGNALING VIA NFKB
Pathways
Gene Ratio
Adjusted p-value
B.
KEGG Pathway Enrichment Analysis
Cytokine-cytokine receptor interaction
Viral protein interaction with cytokine and cytokine receptor
Chemokine signaling pathway
Rheumatoid arthritis
Intestinal immune network for IgA production
Toll-like receptor signaling pathway
Pathways
Gene Ratio
p.adjust
Count
D.
Reactome Pathway Enrichment Analysis
Peptide ligand-binding receptors
Class A/1 (Rhodopsin-like receptors)
GPCR ligand binding
Signaling by Interleukins
Chemokine receptors bind chemokines
G alpha (i) signalling events
Interleukin-10 signaling
Pathways
Gene Ratio
p.adjust
Count
F.
MSigDB Enrichment Analysis
HALLMARK KRAS SIGNALING UP
HALLMARK MTORC1 SIGNALING
HALLMARK TNFA SIGNALING VIA NFKB
Pathways
Gene Ratio
p.adjust
Count

A.

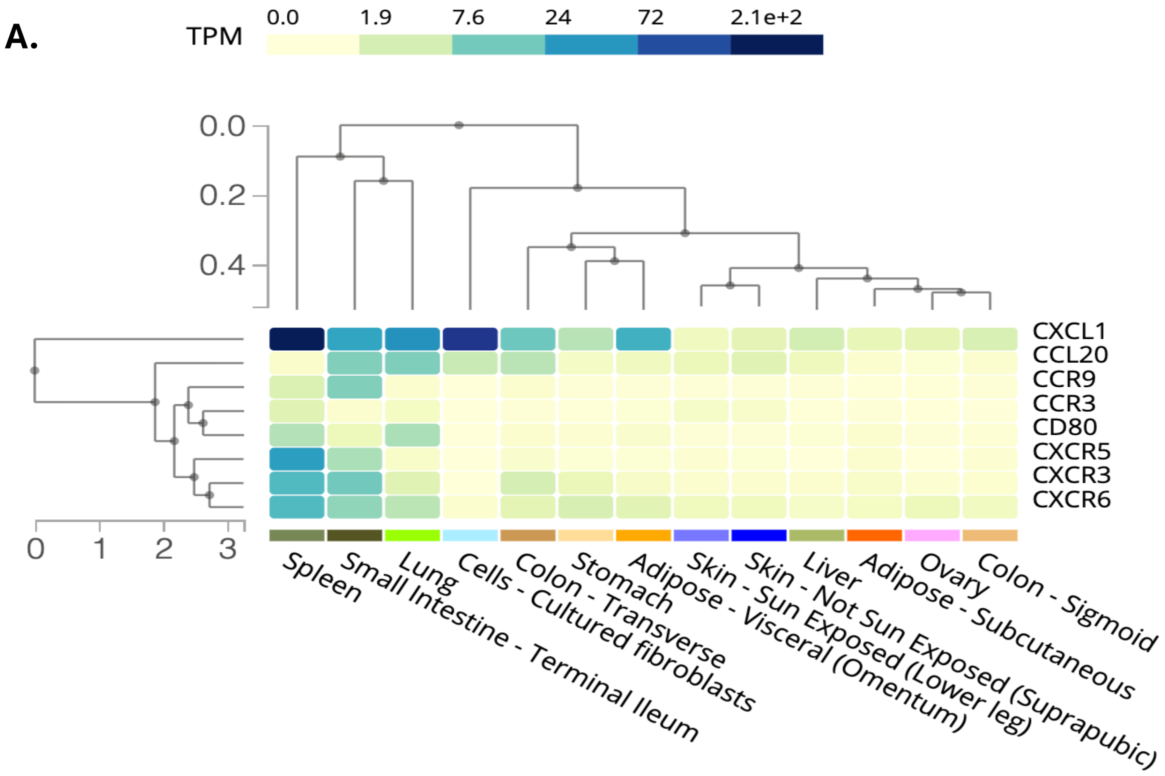

Expression patterns of hub genes across normal tissues

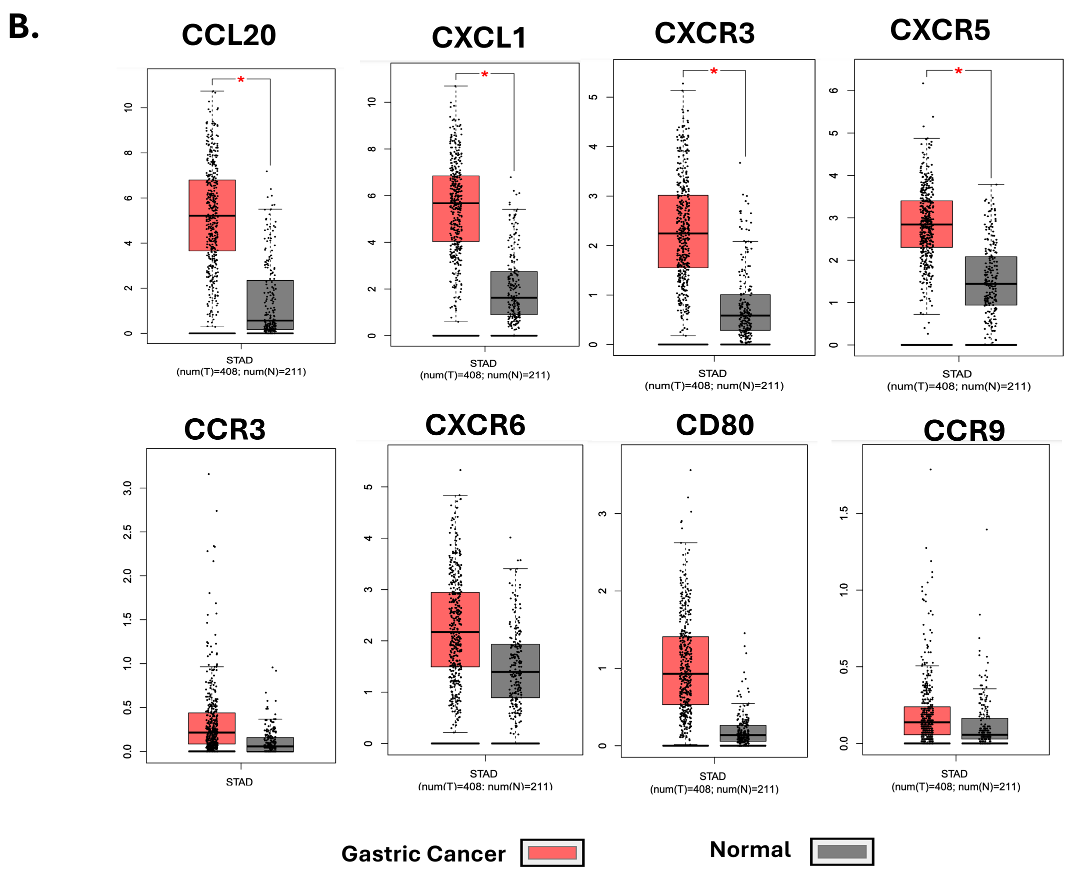

Expression patterns of hub genes in Gastric Cancer Vs Normal Tissue

C.

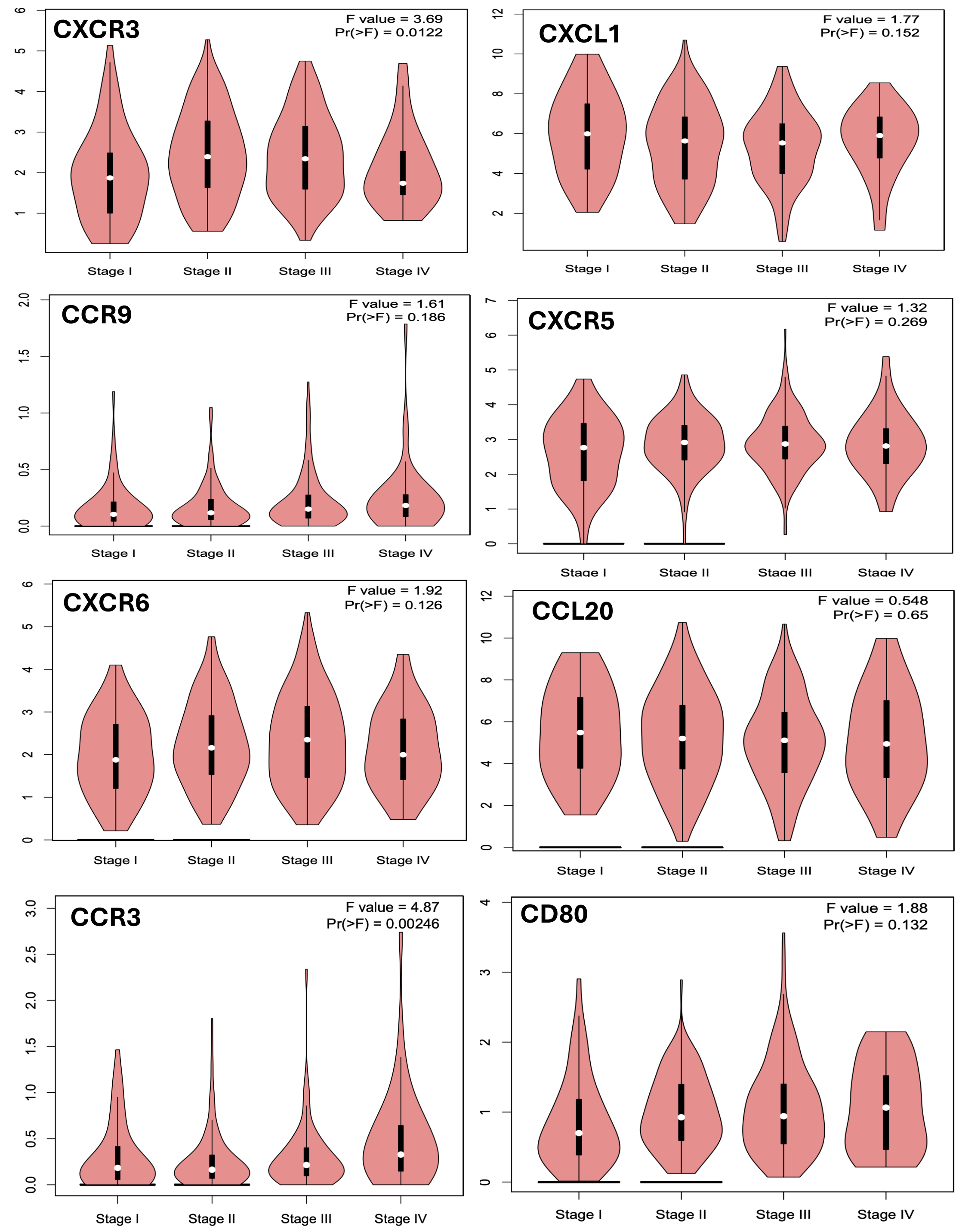

Expression Patterns of Hub Genes Across Gastric Cancer Stages

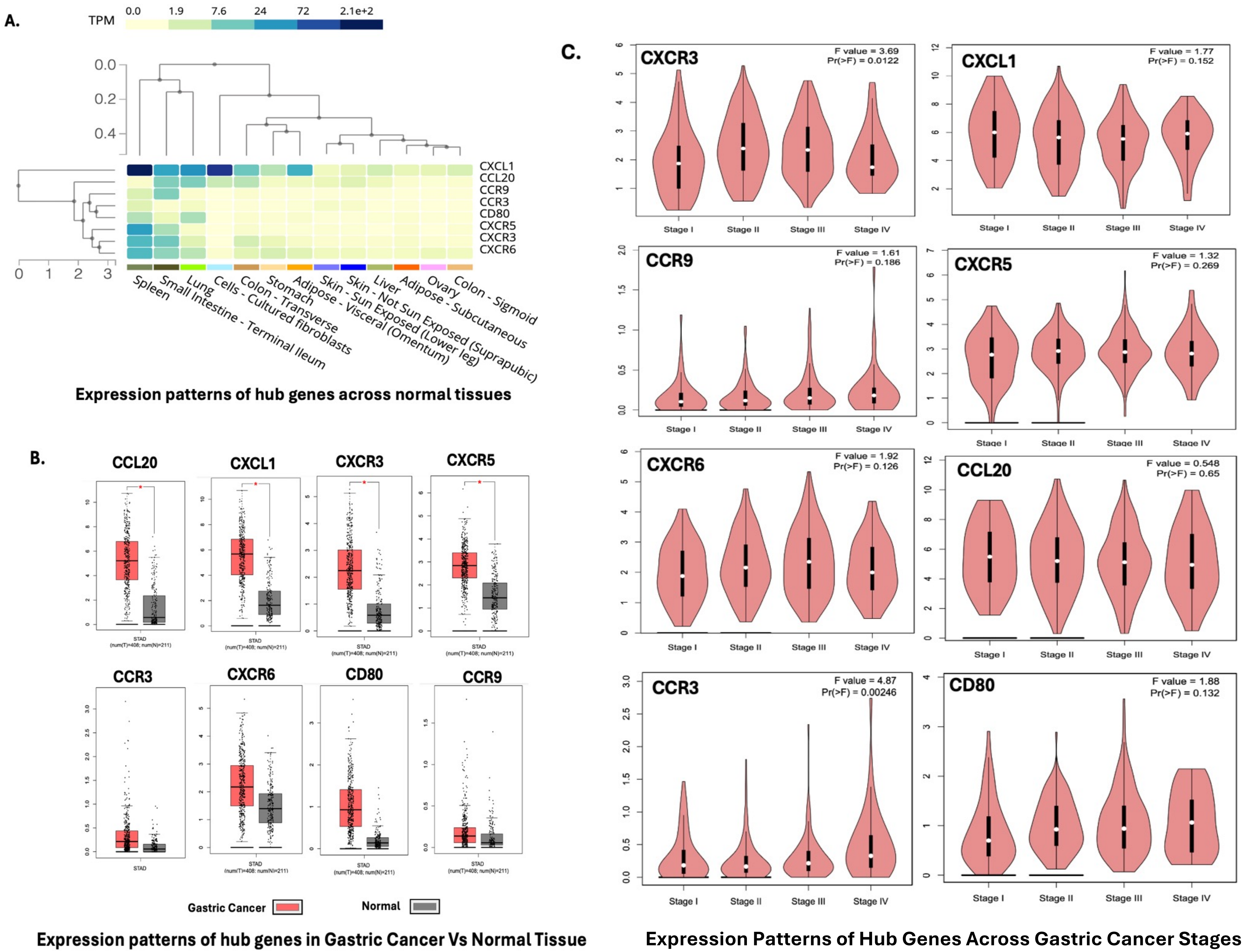
A.
TPM
0.0
1.9
7.6
24
72
2.1e+2
CXCL1
CCL20
CCR9
CCR3
CD80
CXCR5
CXCR3
CXCR6
Spleen
Small Intestine - Terminal Ileum
Lung
Cells - Cultured fibroblasts
Colon - Transverse
Stomach
Adipose - Visceral (Omentum)
Skin - Sun Exposed (Lower leg)
Skin - Not Sun Exposed (Suprapubic)
Liver
Adipose - Subcutaneous
Ovary
Colon - Sigmoid
Expression patterns of hub genes across normal tissues
B.
CCL20
CXCL1
CXCR3
CXCR5
CCR3
CXCR6
CD80
CCR9
Gastric Cancer
Normal
Expression patterns of hub genes in Gastric Cancer Vs Normal Tissue
C.
CXCR3
F value = 3.69
Pr(>F) = 0.0122
CXCL1
F value = 1.77
Pr(>F) = 0.152
CCR9
F value = 1.61
Pr(>F) = 0.186
CXCR5
F value = 1.32
Pr(>F) = 0.269
CXCR6
F value = 1.92
Pr(>F) = 0.126
CCL20
F value = 0.548
Pr(>F) = 0.65
CCR3
F value = 4.87
Pr(>F) = 0.00246
CD80
F value = 1.88
Pr(>F) = 0.132
Stage I
Stage II
Stage III
Stage IV
Expression Patterns of Hub Genes Across Gastric Cancer Stages

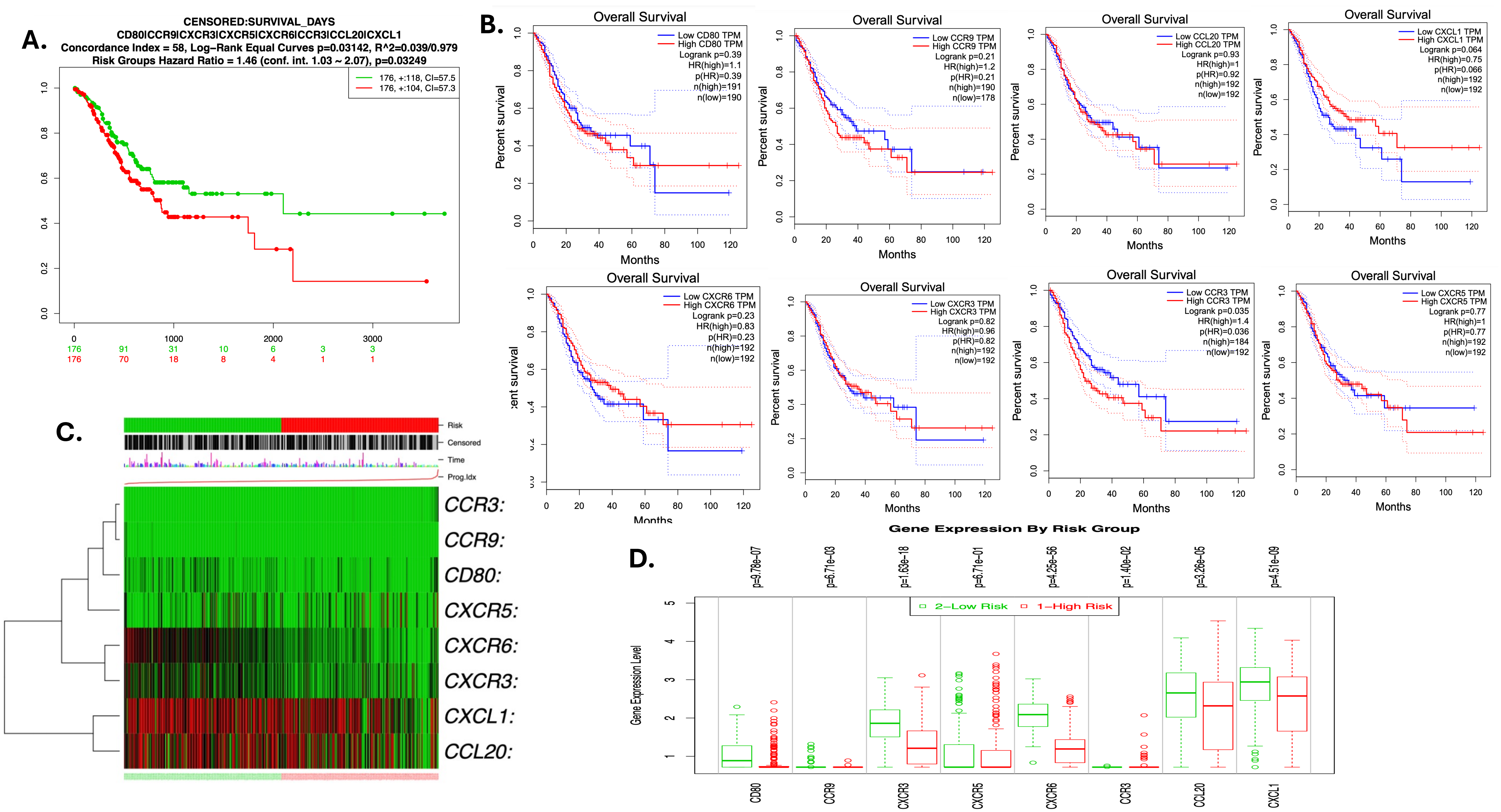

A.
CENSORED:SURVIVAL_DAYS
CD80|CCR9|CXCR3|CXCR5|CXCR6|CCR3|CCL20|CXCL1
Concordance Index = 58, Log−Rank Equal Curves p=0.03142, R^2=0.039/0.979
Risk Groups Hazard Ratio = 1.46 (conf. int. 1.03 ~ 2.07), p=0.03249
176, +:118, CI=57.5
176, +:104, CI=57.3
B.
Overall Survival
Low CD80 TPM
High CD80 TPM
Logrank p=0.39
HR(high)=1.1
p(HR)=0.39
n(high)=191
n(low)=190
Low CCR9 TPM
High CCR9 TPM
Logrank p=0.21
HR(high)=1.2
p(HR)=0.21
n(high)=190
n(low)=178
Low CCL20 TPM
High CCL20 TPM
Logrank p=0.93
HR(high)=1
p(HR)=0.92
n(high)=192
n(low)=192
Low CXCL1 TPM
High CXCL1 TPM
Logrank p=0.064
HR(high)=0.75
p(HR)=0.066
n(high)=192
n(low)=192
Low CXCR6 TPM
High CXCR6 TPM
Logrank p=0.23
HR(high)=0.83
p(HR)=0.23
n(high)=192
n(low)=192
Low CXCR3 TPM
High CXCR3 TPM
Logrank p=0.82
HR(high)=0.96
p(HR)=0.82
n(high)=192
n(low)=192
Low CCR3 TPM
High CCR3 TPM
Logrank p=0.035
HR(high)=1.4
p(HR)=0.036
n(high)=184
n(low)=192
Low CXCR5 TPM
High CXCR5 TPM
Logrank p=0.77
HR(high)=1
p(HR)=0.77
n(high)=192
n(low)=192
Percent survival
Months
C.
Risk
Censored
Time
Prog.Idx
CCR3:
CCR9:
CD80:
CXCR5:
CXCR6:
CXCR3:
CXCL1:
CCL20:
D.
Gene Expression By Risk Group
p=9.78e-07
p=6.71e-03
p=1.63e-18
p=6.71e-01
p=4.25e-56
p=1.40e-02
p=3.26e-05
p=4.51e-09
2−Low Risk
1−High Risk
Gene Expression Level
CD80
CCR9
CXCR3
CXCR5
CXCR6
CCR3
CCL20
CXCL1

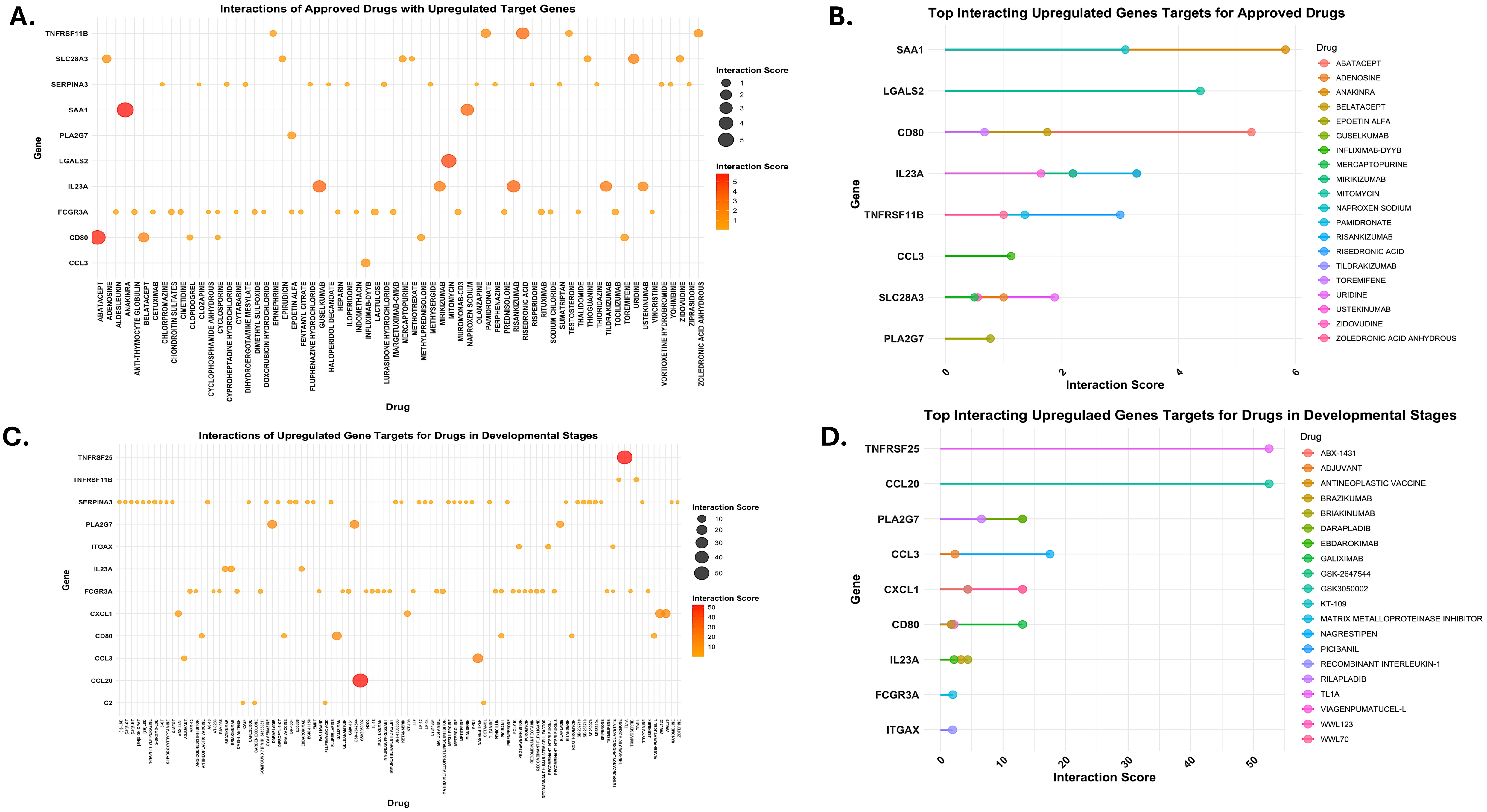
A.
Interactions of Approved Drugs with Upregulated Target Genes
TNFRSF11B
SLC28A3
SERPINA3
SAA1
PLA2G7
LGALS2
IL23A
FCGR3A
CD80
CCL3
Gene
ABATACEPT
ADENOSINE
ALDESLEUKIN
ANAKINRA
ANTI-THYMOCYTE GLOBULIN
BELATACEPT
CETUXIMAB
CHLORPROMAZINE
CHONDROITIN SULFATES
CIMETIDINE
CLOPIDOGREL
CLOZAPINE
CYCLOPHOSPHAMIDE ANHYDROUS
CYCLOSPORINE
CYPROHEPTADINE HYDROCHLORIDE
CYTARABINE
DIHYDROERGOTAMINE MESYLATE
DIMETHYL SULFOXIDE
DOXORUBICIN HYDROCHLORIDE
EPINEPHRINE
EPIRUBICIN
EPOETIN ALFA
FENTANYL CITRATE
FLUPHENAZINE HYDROCHLORIDE
GUSELKUMAB
HALOPERIDOL DECANOATE
HEPARIN
ILOPERIDONE
INDOMETHACIN
INFLIXIMAB-DYYB
LACTULOSE
LURASIDONE HYDROCHLORIDE
MARGETUXIMAB-CMKB
MERCAPTOPURINE
METHOTREXATE
METHYLPREDNISOLONE
METHYSERGIDE
MIRIKIZUMAB
MITOMYCIN
MUROMONAB-CD3
NAPROXEN SODIUM
OLANZAPINE
PAMIDRONATE
PERPHENAZINE
PREDNISOLONE
RISANKIZUMAB
RISEDRONIC ACID
RISPERIDONE
RITUXIMAB
SODIUM CHLORIDE
SUMATRIPTAN
TESTOSTERONE
THALIDOMIDE
THIOGUANINE
THIORIDAZINE
TILDRAKIZUMAB
TOCILIZUMAB
TOREMIFENE
URIDINE
USTEKINUMAB
VINCRISTINE
VORTIOXETINE HYDROBROMIDE
YOHIMBINE
ZIDOVUDINE
ZIPRASIDONE
ZOLEDRONIC ACID ANHYDROUS
Drug
Interaction Score
1
2
3
4
5
Interaction Score
5
4
3
2
1
B.
Top Interacting Upregulated Genes Targets for Approved Drugs
SAA1
LGALS2
CD80
IL23A
TNFRSF11B
CCL3
SLC28A3
PLA2G7
Gene
0
2
4
6
Interaction Score
Drug
ABATACEPT
ADENOSINE
ANAKINRA
BELATACEPT
EPOETIN ALFA
GUSELKUMAB
INFLIXIMAB-DYYB
MERCAPTOPURINE
MIRIKIZUMAB
MITOMYCIN
NAPROXEN SODIUM
PAMIDRONATE
RISANKIZUMAB
RISEDRONIC ACID
TILDRAKIZUMAB
TOREMIFENE
URIDINE
USTEKINUMAB
ZIDOVUDINE
ZOLEDRONIC ACID ANHYDROUS
C.
Interactions of Upregulated Gene Targets for Drugs in Developmental Stages
TNFRSF25
TNFRSF11B
SERPINA3
PLA2G7
ITGAX
IL23A
FCGR3A
CXCL1
CD80
CCL3
CCL20
C2
Gene
Drug
Interaction Score
10
20
30
40
50
Interaction Score
50
40
30
20
10
D.
Top Interacting Upregulaed Genes Targets for Drugs in Developmental Stages
TNFRSF25
CCL20
PLA2G7
CCL3
CXCL1
CD80
IL23A
FCGR3A
ITGAX
Gene
0
10
20
30
40
50
Interaction Score
Drug
ABX-1431
ADJUVANT
ANTINEOPLASTIC VACCINE
BRAZIKUMAB
BRIAKINUMAB
DARAPLADIB
EBDAROKIMAB
GALIXIMAB
GSK-2647544
GSK3050002
KT-109
MATRIX METALLOPROTEINASE INHIBITOR
NAGRESTIPEN
PICIBANIL
RECOMBINANT INTERLEUKIN-1
RILAPLADIB
TL1A
VIAGENPUMATUCEL-L
WWL123
WWL70

A.

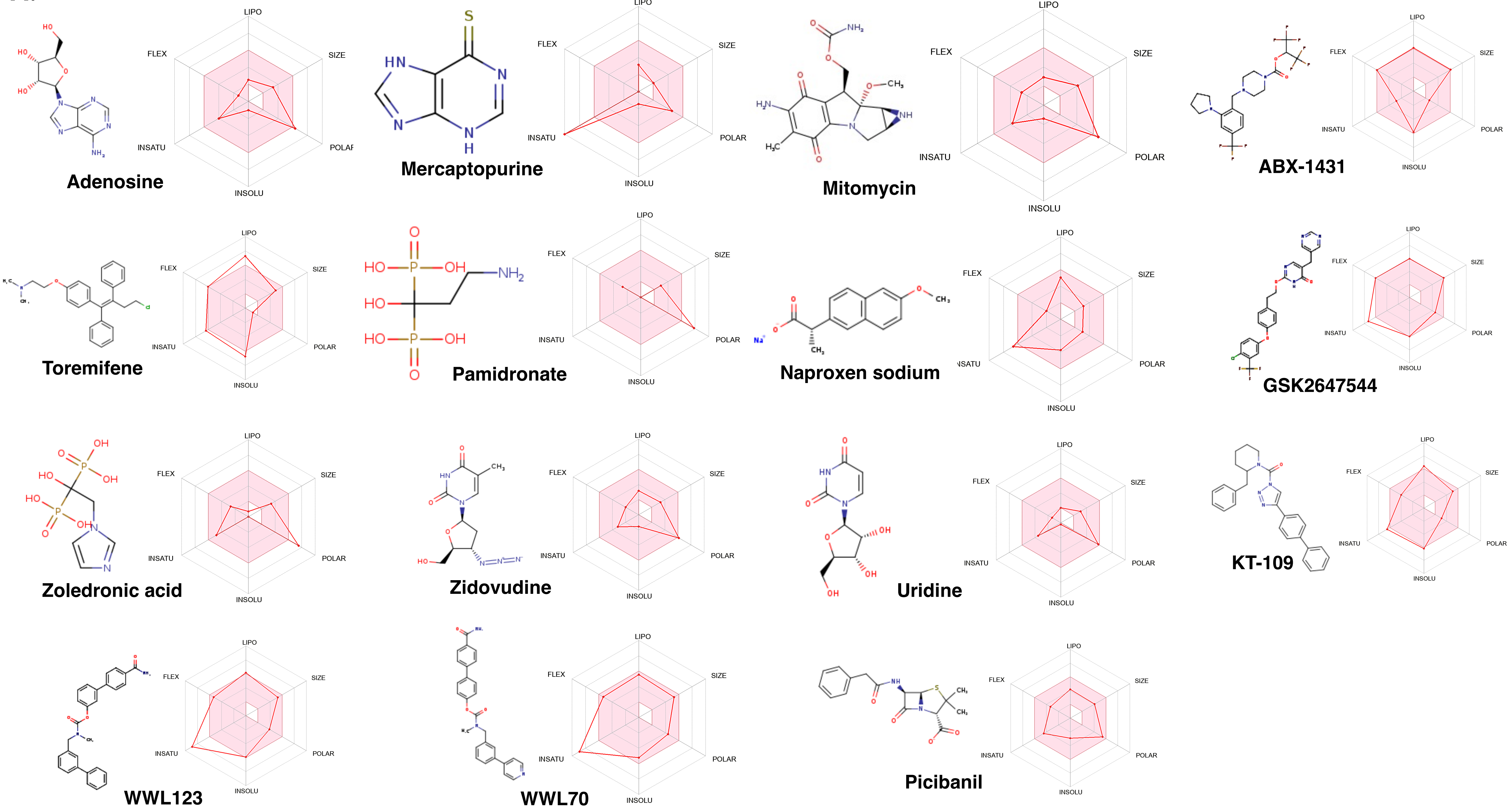
Adenosine
Mercaptopurine
Mitomycin
ABX-1431
Toremifene
Pamidronate
Naproxen sodium
GSK2647544
Zoledronic acid
Zidovudine
Uridine
KT-109
WWL123
WWL70
Picibanil
LIPO
SIZE
POLAR
INSOLU
INSATU
FLEX

B.

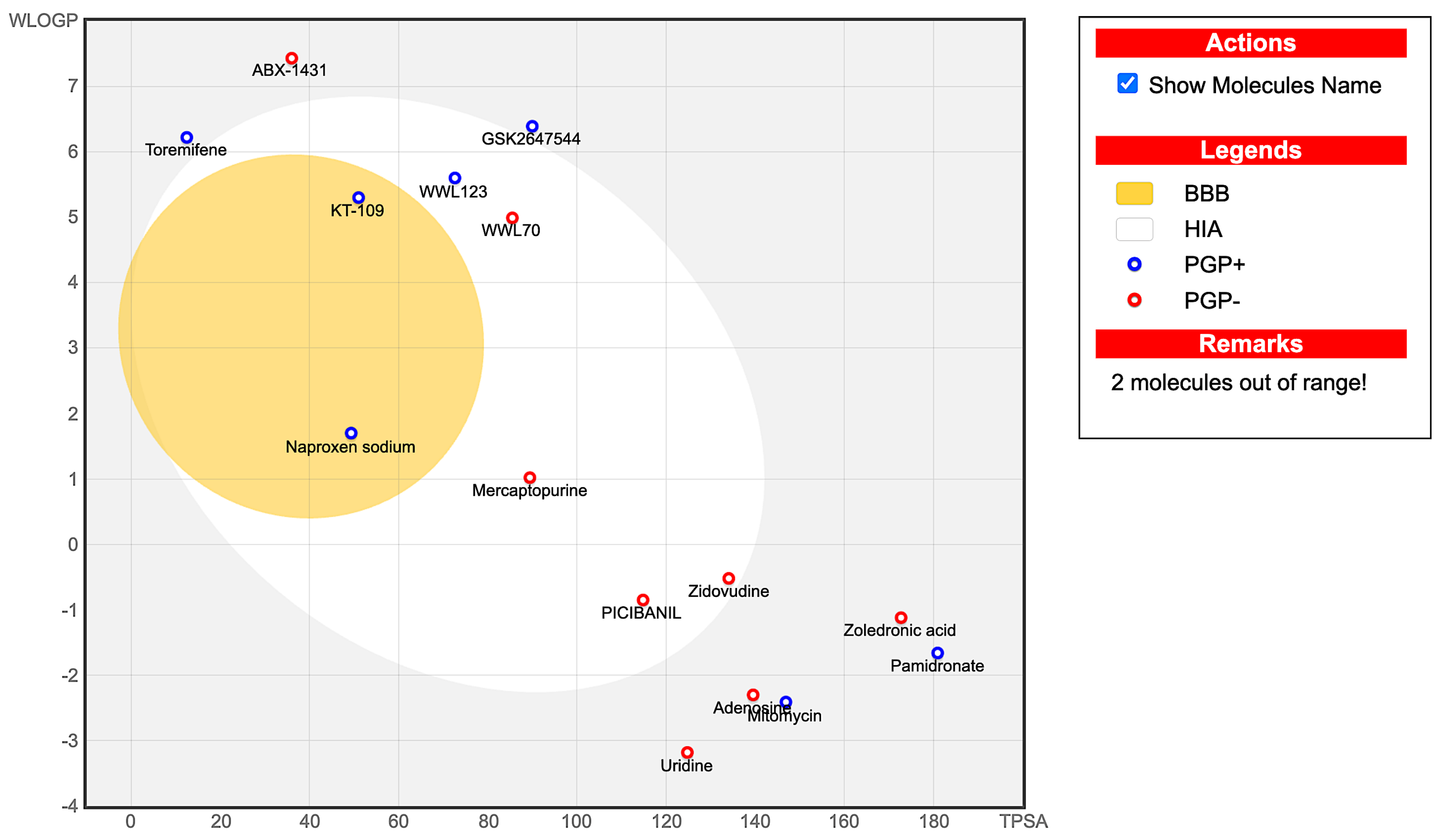
WLOGP
TPSA
ABX-1431
Toremifene
GSK2647544
WWL123
KT-109
WWL70
Naproxen sodium
Mercaptopurine
Zidovudine
PICIBANIL
Zoledronic acid
Pamidronate
Adenosine
Mitomycin
Uridine
Actions
Show Molecules Name
Legends
BBB
HIA
PGP+
PGP-
Remarks
2 molecules out of range!

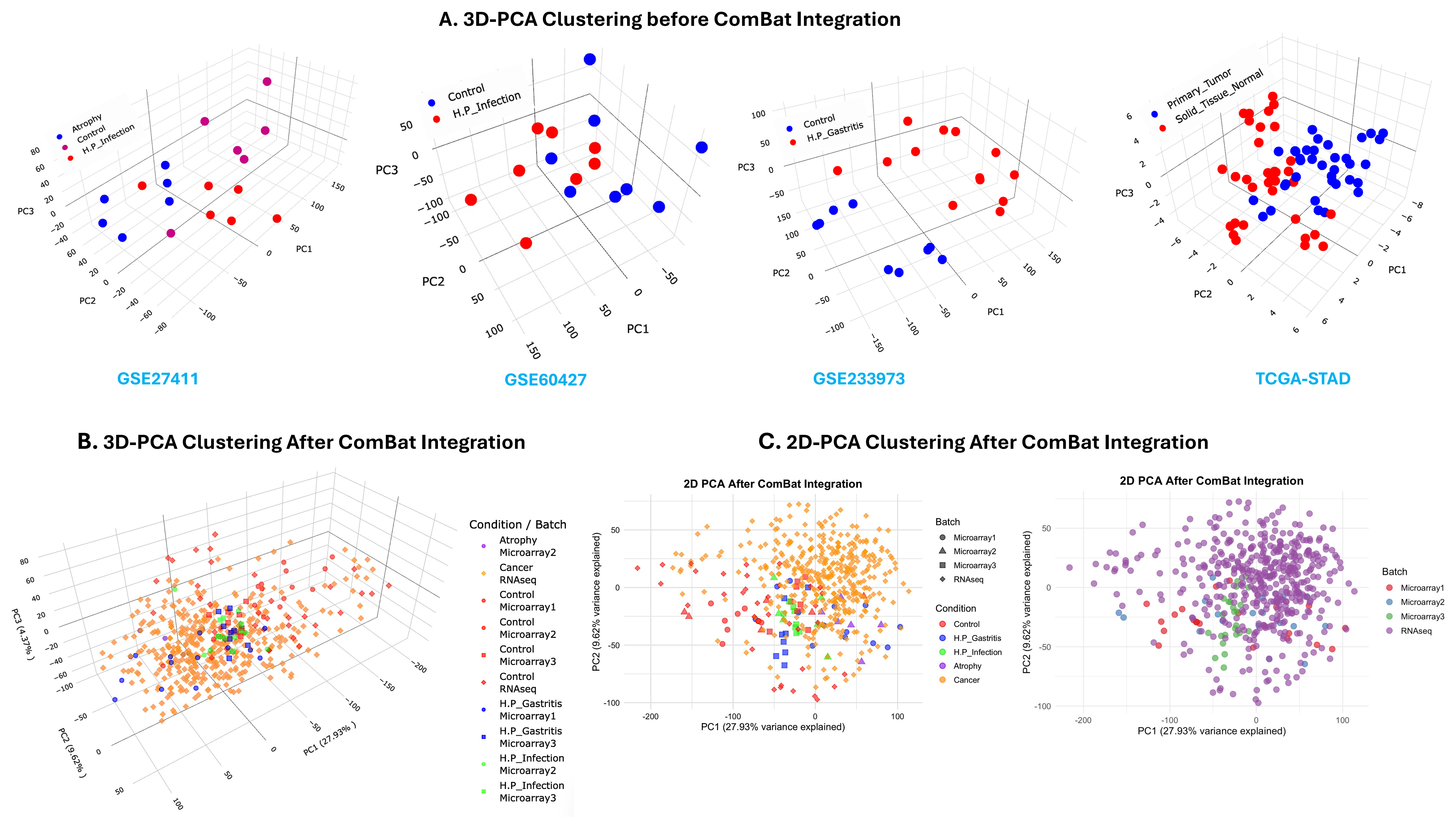
A. 3D-PCA Clustering before ComBat Integration
Atrophy
Control
H.P_Infection
PC1
PC2
PC3
GSE27411
Control
H.P_Infection
PC1
PC2
PC3
GSE60427
Control
H.P_Gastritis
PC1
PC2
PC3
GSE233973
Primary_Tumor
Solid_Tissue_Normal
PC1
PC2
PC3
TCGA-STAD
B. 3D-PCA Clustering After ComBat Integration
PC1 (27.93%)
PC2 (9.62%)
PC3 (4.37%)
Condition / Batch
Atrophy Microarray2
Cancer RNAseq
Control Microarray1
Control Microarray2
Control Microarray3
Control RNAseq
H.P_Gastritis Microarray1
H.P_Gastritis Microarray3
H.P_Infection Microarray2
H.P_Infection Microarray3
C. 2D-PCA Clustering After ComBat Integration
2D PCA After ComBat Integration
PC1 (27.93% variance explained)
PC2 (9.62% variance explained)
Batch
Microarray1
Microarray2
Microarray3
RNAseq
Condition
Control
H.P_Gastritis
H.P_Infection
Atrophy
Cancer
2D PCA After ComBat Integration
PC1 (27.93% variance explained)
PC2 (9.62% variance explained)
Batch
Microarray1
Microarray2
Microarray3
RNAseq
